# Caffeoylquinic Acids and Flavonoids of Fringed Sagewort (*Artemisia frigida* Willd.): HPLC-DAD-ESI-QQQ-MS Profile, HPLC-DAD Quantification, in Vitro Digestion Stability, and Antioxidant Capacity

**DOI:** 10.3390/antiox8080307

**Published:** 2019-08-14

**Authors:** Daniil N. Olennikov, Nina I. Kashchenko, Nadezhda K. Chirikova, Aina G. Vasil’eva, Aydan I. Gadimli, Javanshir I. Isaev, Cecile Vennos

**Affiliations:** 1 Laboratory of Medical and Biological Research, Institute of General and Experimental Biology, Siberian Division, Russian Academy of Science, 6 Sakh’yanovoy Street, Ulan-Ude 670047, Russia; 2 Department of Biochemistry and Biotechnology, North-Eastern Federal University, 58 Belinsky Street, Yakutsk 677027, Russia; 3 Department of Pharmacognosy, Azerbaijan Medical University, Anvar Gasimzade Street 14, Baku AZ1022, Azerbaijan; 4 Regulatory and Medical Scientific Affairs, Padma AG, 1 Underfeldstrasse, CH-8340 Hinwil, Switzerland

**Keywords:** *Artemisia frigida*, Compositae (Asteraceae), caffeoylquinic acids, flavonoids, HPLC, mass spectrometry, chemotaxonamy, antioxidant capacity, ORAC

## Abstract

Fringed sagewort (*Artemisia frigida* Willd., Compositae family) is a well-known medicinal plant in Asian medical systems. Fifty-nine hydroxycinnamates and flavonoids have been found in *A. frigida* herbs of Siberian origin by high-performance liquid chromatography with diode array and electrospray triple quadrupole mass detection (HPLC-DAD-ESI-QQQ-MS). Their structures were determined after mass fragmentation analysis as caffeoylquinic acids, flavone *O*-/*C*-glycosides, flavones, and flavonol aglycones. Most of the discovered components were described in *A. frigida* for the first time. It was shown that flavonoids with different types of substitution have chemotaxonomic significance for species of *Artemisia* subsection Frigidae (section Absinthium). After HPLC-DAD quantification of 16 major phenolics in 21 Siberian populations of *A. frigida* and subsequent principal component analysis, we found substantial variation in the selected compounds, suggesting the existence of two geographical groups of *A. frigida*. The antioxidant activity of *A. frigida* herbal tea was determined using 2,2-diphenyl-1-picrylhydrazyl free radical (DPPH^•^) and hydrophilic/lipophilic oxygen radical absorbance capacity (ORAC) assays and DPPH^•^-HPLC profiling, revealing it to be high. The effect of digestive media on the phenolic profile and antioxidant capacity of *A. frigida* herbal tea was assessed under simulated gastrointestinal digestion. We found a minor reduction in caffeoylquinic acid content and ORAC values, but remaining levels were satisfactory for antioxidant protection. These results suggest that *A. frigida* and its food derivate herbal tea could be recommended as new plant antioxidants rich in phenolics.

## 1. Introduction

Traditional healing treatments refer to the collective knowledge, skills and practices that are based on the theories, values and personal experiences developed and used by indigenous people of different cultures to improve health, avoid and reduce diseases and their spread, or as complete cures of both physical and mental health conditions [1,2]. The investigation of traditional healing procedures in unique regions such as Siberia is of particular interest due to the large variety of ethnic groups. The territory of Siberia extends eastwards from the Ural Mountains to the watershed between the Pacific and Arctic drainage basins. The local residents mostly passed traditional healing knowledge on orally from one generation of healers to the next. The study of the use of local flora of a particular region or culture by native people is termed ethnobotany [3]. The native populations of different regions of Siberia (yakuts, buryats, tuvans, soyots) have used the plants in their environs for different purposes since ancient times.

**Figure 1:**
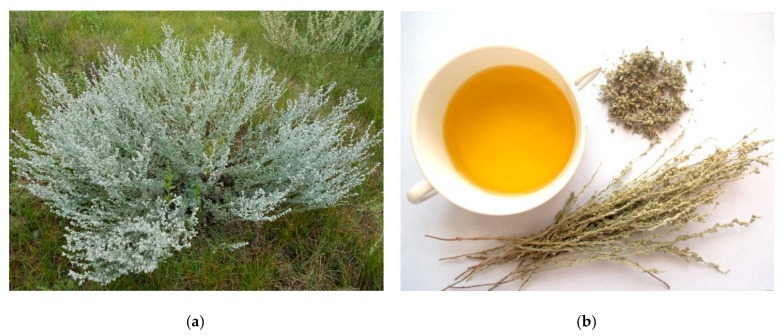
(**a**) Artemisia frigida plants in their natural habitat (Selenginskii Region, Buryatia Republic, Eastern Siberia); (**b**) dried herb, herbal powder and herbal tea of A. frigida.

*Artemisia frigida* Willd. (fringed sagewort) is one of the plant species most used by nomadic people for its therapeutic properties (Figure 1). As a member of the Compositae family, it is widespread in the steppe regions of Siberia and Mongolia, as well as on the prairies of North America [4]. There are reports of both external and internal uses of *A. frigida* herbs by nomads [5,6,7,8,9], as well as the addition of *A. frigida* to cold baths with water taken from mineral hot and cold springs known as “arshans” [7]. Cold spring water is heated in barrels using hot stones, then *A. frigida* herbs are added. This treatment is believed to prevent colds. The application of fringed sagebrush as an herbal tea is of the greatest interest. Herbal tea from aerial parts of *A. frigida* is referred to as *sagaan aya tea*. Sagaan aya tea is used to treat many diseases, in particular those caused by oxidative stress—a process that can trigger cell damage [10]. In particular, during surveys of nomadic populations, we found that sagaan aya tea was used to treat a number of diseases in the pathogenesis of which oxidative stress is thought to be involved, such as hypertension, diabetes, cardiovascular diseases, etc. (supplementary materials Table S1).

Chemical investigations of *Artemisia* species often focus on artemisinin and other sesquiterpenes, ignoring phenolic compounds despite their well-known potent antioxidant properties [11]. According to literature data, a chemical study of phenolic compounds was carried out on *A. frigida* growing in North America [12,13], China [14,15,16,17], Inner Mongolia [18,19] and Russia [20]. As a result, only 37 substances, such as flavonoids of the flavone, flavonol and biflavone groups; coumarins and phenylpropanoids, were isolated and characterized (Table S2). Derivatives of luteolin, 6-hydroxyluteolin and 3′,5′-demethoxytricin in the form of aglycones and glycosides are the main structural types of flavones in *A. frigida*. Rare *O*-glucuronides and 2′′-*O*-glucuronyl-glucuronides were detected only in raw materials collected in China and Inner Mongolia [17,18,19]. In specimens of Russian origin, only *C*-glycosides of apigenin were found [20], and luteolin-7-*O*-glucoside (cynaroside) was revealed in North American samples [12,13]. The only biflavone glycoside (8-*O*-8′′′-biluteolin-7,7′′′-*O*-glucuronide) was detected in the aerial part of *A. frigida* from the vicinity of the Tongliao district (China) [18]. North American samples of *A. frigida* were characterized by the ability to accumulate derivatives of luteolin and heptahydroxyflavone, whereas the glycosides of tricetin were found in specimens from China and Mongolia. Caffeoylquinic acids are widely distributed in species of the genus *Artemisia* [21], but their presence in *A. frigida* has not been established. 

Thus, we decided to characterize caffeoylquinic acids and flavonoids of *A. frigida* using high-performance liquid chromatography (HPLC) with both diode array detection (DAD) and electrospray ionization triple quadrupole mass spectrometry (ESI-QQQ-MS) to compare the cumulative content of flavonoids and caffeoylquinic acids in the herb of the Siberian populations of fringed sagewort, according to habitat, by HPLC-DAD quantification. At the second stage of research, based on the potential bioactivity of phenolic compounds, we investigated the digestive stability of caffeoylquinic acids and flavonoids from *A. frigida* herbal tea. Sagaan aya tea was processed through simulated gastric and small intestinal digestion, mimicking the physicochemical and biochemical changes that occur in the upper gastrointestinal tract. Finally, because antioxidant properties of *A. frigida* herbal tea may be an important means of protecting the gastrointestinal tract itself, the total antioxidant capacity of non-treated herbal tea and herbal tea after gastric and intestinal phases from sagaan aya tea was also assessed.

## 2. Materials and Methods

### 2.1. Plant Materials and Chemicals

The information about samples of herb of *A. frigida* is listed in Table 1. The species were authenticated by Prof. T.A. Aseeva (IGEB SB RAS, Ulan-Ude, Russia). Plant material was dried and powdered before analysis.

The chemicals were purchased from ChemFaces (Wuhan, Hubei, PRC)—1-caffeoylquinic acid (Cat. No. CFN99121, ≥98%), cirsilineol (Cat. No. CFN90418, ≥98%), cirsimaritin (Cat. No. CFN97126, ≥98%), chrysoeriol (Cat. No. CFN98785, ≥98%), eupatorin (Cat. No. CFN97418, ≥98%), genkwanin (Cat. No. CFN98670, ≥98%), hispidulin (Cat. No. CFN99491, ≥98%), jaceosidine (Cat. No. CFN90386, ≥98%), luteolin-4′-*O*-glucoside (Cat. No. CFN93776, ≥98%), thermopsoside (Cat. No. CFN93021, ≥98%), vicenin-2 (Cat. No. CFN92031, ≥98%), velutin (Cat. No. CFN98290, ≥98%); ChemNorm Co. LTD (Wuhan, China)—chrysoeriol-4′-O-glucoside (Cat. No. TBZ2828, ≥98%); Extrasynthese (Lyon, France)—apigenin-7-*O*-glucoside (Cat. No. 1004 S, ≥99%), cynaroside (Cat. No. 1126 S, ≥98%), orientin (Cat. No. 1054 S, ≥99%); Sigma-Aldrich (St. Louis, MO, USA)—acacetin (Cat. No. 49975, ≥95%), acetic acid (Cat. No. 695092, ≥99.7%), acetone (Cat. No. 179124, ≥99.5%), acetonitrile for HPLC (Cat. No 34851, ≥99.9%), aluminum chloride hydrate (Cat. No. 229393), ammonium chloride (Cat. No. 213330, ≥99.5%), apigenin (Cat. No. 42251, ≥99%), 2,2′-azobis(2-methylpropionamidine) dihydrochloride (Cat. No. 440914, ≥97%), bile extract porcine (Cat. No. B8631), bovine serum albuminum (Cat. No. 05470, ≥98%), 4-*O*-caffeoylquinic acid (Cat. No. 65969, ≥98%), 5-*O*-caffeoylquinic acid (Cat. No. 94419, ≥98%), 1,3-di-*O*-caffeoylquinic acid (Cat. No. D8196, ≥98%), 1,5-dicaffeoylquinic acid (Cat. No. 16917, ≥98%), 3,4-di ≥90%), 3,5-di-*O*-caffeoylquinic-*O*-caffeoylquinic acid (Cat. No. SMB00224, acid (Cat. No. SMB00131, ≥95%), 4,5-di-*O*-caffeoylquinic acid (Cat. No. SMB00221, ≥85%), calcium chloride (Cat. No. C8106), chloroform (Cat. No. C2432, ≥99.5%), cyanidin 3-*O*-glucoside chloride (Cat. No. PHL89616, ≥98%), cynaroside (Cat. No. 49968, ≥98%), dialysis tubing benzoylated (Cat. No. D7884, average flat width 32 mm (1.27 in.), 2,2-diphenyl-1-picrylhydrazyl (Cat. No. D9132), fluorescein (Cat. No. 46955), glucose (Cat. No. G8270, ≥99.5%), hexane (Cat. No. 208752, ≥95%), hydrochloric acid (Cat. No. 320331, 37%), isoschaftoside (Cat. No. PHL83324, ≥90%), isoorientin (Cat. No. 02187, ≥98%), isovitexin (Cat. No. 17804, ≥98%), lithium perchlorate (Cat. No. 431567, ≥99%), loganic acid (Cat. No. SMB00231, ≥95%), magnesium chloride (Cat. No. M8266, ≥98%), methanol (Cat. No. 322415, ≥99.8%), methyl-β-cyclodextrin (Cat. No. 332615), oxalic acid (Cat. No. 194131, ≥98%), pancreatin from porcine pancreas (Cat. No. P7545, 8 × USP specifications), penta-*O*-galloyl-β-d-glucose hydrate (Cat. No. G7548, ≥96%), pepsin from porcine gastric mucosa (Cat. No. P6887, 3200–4500 units/mg protein), perchloric acid 70% (Cat. No. 311421, ≥99%), phenol (Cat. No. 242322, 99%), potassium chloride (Cat. No. P9333, ≥99%), potassium phosphate monobasic (Cat. No. 795488, 99%), schaftoside (Cat. No. PHL83325, ≥95%), sodium bicarbonate (Cat. No. S6014, ≥99.7%), sodium chloride (Cat. No. S7653, 99.5%), sodium hydroxide (Cat. No. 221465, ≥97%), sodium phosphate monobasic (Cat. No. S8282, ≥99%), stachydrine chloride (Cat. No. PHL89799, ≥95%), sulfuric acid (Cat. No. 258105, ≥95%), tricin (Cat. No. PHL80987, ≥95%), trifluoroacetic acid (Cat. No. T6508, ≥99%), trolox (Cat. No. 238813, ≥97%), vitexin (Cat. No. 49513, ≥95%), umbelliferone (Cat. No. 54826, 98%). 6-Hydroxyluteolin-7-*O*-glucoside, nepitrin (nepetin-7-*O*-glucoside), diosmetin-7-*O*-glucoside, rhaunoside F (nepetin-3′-*O*-glucoside), rhaunoside C (6-hydroxyluteolin-4′-*O*-glucoside), and nepetin-4′-*O*-glucoside were isolated previously from *Rhaponticum uniflorum* [22], 1,3,5-tri-*O*-caffeoylquinic acid, 1,4,5-tri-*O*-caffeoylquinic acid, 3,4,5-tri-*O*-caffeoylquinic acid and luteolin-3′,4′-dimethyl ether were isolated from *Calendula officinalis* [23] and *Thymus baicalensis* [24].

Equipment used for UV-Vis spectrophotometry was SF-2000 UV-Vis-spectrophotometer (OKB Specter, St. Peterburg, Russia).

### 2.2. Total Extract and Herbal Tea Preparation

For preparation of the total extract of *A. frigida* herb the powdered sample (100 g) was extracted three times in a conical glass flask (2 L) with 70% methanol (1 L) with stirring and sonification for 60 min at 50 °C with ultrasound power of 100 W and the frequency 35 kHz. The resulted extracts were filtered through a cellulose filter, combined, evaporated in vacuo until dryness, and stored at 4 °C until further chemical composition analysis and bioactivity assays. The yield of total extract of *A. frigida* herb was 22.14 g, respectively. 

For the preparation of herbal tea, an accurately weighted *A. frigida* herb (1 g) was mixed with 100 mL of distilled water, and then heated on a heater plate and boiled for 10 min. The mixture was left to stand at room temperature for 15 min, and then filtered under reduced pressure in volumetric flasks (100 mL). The final volume was reduced to initial sign and filtered through a 0.22 μm PTFE syringe filter before analysis. 

### 2.3. High-Performance Liquid Chromatography with Diode Array Detection and Electrospray Ionization Triple Quadrupole Mass Spectrometric Detection (HPLC-DAD-ESI-QQQ-MS) Profiling Condition

Reversed-phase high-performance liquid chromatography with diode array detection and electrospray ionization triple quadrupole mass spectrometric detection (HPLC-DAD-ESI-QQQ-MS) procedure was used for phenolic compounds profiling. Experiments were performed on an LCMS 8050 liquid chromatograph coupled with diode-array-detector and triple-quadrupole electrospray ionization detector (Shimadzu, Columbia, MD, USA), using a GLC Mastro C18 column (150 × 2.1 mm, Ø 3 μm; Shimadzu, Kyoto, Japan), column temperature was 35 °C. Eluent A was 0.5% formic acid in water and eluent B was 0.5% formic acid in acetonitrile. The injection volume was 1 μL, and elution flow was 100 μL/min. Gradient program: 0.0–1.0 min 5–21% B, 1.0–2.0 min 21–38% B, 2.0–2.7 min 38–55% B, 2.7–3.5 min 55–61% B, 3.5–5.0 min 61–94% B. The DAD acquisitions were performed in the range of 200–600 nm and chromatograms were integrated at 330 nm. MS detection was performed in negative ESI mode using the parameters as follows: temperature levels of ESI interface, desolvation line and heat block were 300 °C, 250 °C and 400 °C, respectively. The flow levels of nebulizing gas (N_2_), heating gas (air) and collision-induced dissociation gas (Ar) were 3 L/min, 10 L/min and 0.3 mL/min, respectively. The MS and MS/MS spectra were both recorded in negative mode (−3 kV source voltage) by scanning in the range of *m*/*z* 100–1900 at the collision energy of 10–45 eV.

### 2.4. HPLC-DAD Quantification Condition

HPLC-DAD analysis was performed as described in Section 2.3 and chromatograms were recorded at 330 nm. To prepare the stock solutions of reference standards, 8 mg of 4-*O*-caffeoylquinic acid, 5-*O*-caffeoylquinic acid, 3,4-di-*O*-caffeoylquinic acid, 3,5-di-*O*-caffeoylquinic acid, 4,5-di-*O*-caffeoylquinic acid, 3,4,5-tri-*O*-caffeoylquinic acid, vicenin-2, isoorientin, cynaroside, apigenin, hispidulin, jaseosidine, luteolin-3′,4′-dimethyl ester, eupatorin, acacetin, and cirsimaritin were accurately weighed and individually dissolved in methanol in volumetric flasks (1 mL). The external standard calibration curve was generated using eight data points, covering the concentration ranges 1–500 µg/mL. The calibration curves were created by plotting the concentration levels versus the peak area. All the analyses were carried out in triplicate and the data were expressed as mean value ± standard deviation (SD). For preparation of sample solution, an accurately weighted powdered plant (40 mg) was placed in an Eppendorf tube, 1 mL of 60% ethanol was added, and the mixture was weighted. Then the sample was extracted in an ultrasonic bath for 30 min at 50 °C. After cooling, the tube weight was reduced to initial sign, and the resultant extract was filtered through a 0.22 μm PTFE syringe filter before injection into the HPLC system for analysis. 

### 2.5. Validation Analysis

The linearity of HPLC-DAD quantification method was studied by injecting five concentrations (1–500 µg/mL) of the 16 reference standards (4-*O*-caffeoylquinic acid, 5-*O*-caffeoylquinic acid, 3,4-di-*O*-caffeoylquinic acid, 3,5-di-*O*-caffeoylquinic acid, 4,5-di-*O*-caffeoylquinic acid, 3,4,5-tri-*O*-caffeoylquinic acid, vicenin-2, isoorientin, cynaroside, apigenin, hispidulin, jaseosidine, luteolin-3′,4′-dimethyl ester, eupatorin, acacetin, cirsimaritin). Results from each analysis were averaged and subjected to regression analysis. Limits of detection (LOD) and quantification (LOQ) were determined using the following equations: LOD = (3.3 × *S*_YX_)/*a*; LOQ = (10 × *S*_YX_)/*a*, where *S*_YX_ is a standard deviation of the response (Y intercept) and *a* is a slope of calibration curve. Scopoletin-7-*O*-neohesperidoside (100 µg/mL) spiked in reference standards mixture was used as internal standard. Intra- and inter-day variations, which are presented in terms of percent relative standard deviation (%RSD) of the analyte’s peak area and variability assessed the precision of the HPLC-DAD quantification. For the intra-day variability test, the mixture solution containing 16 reference standards was analysed for five replicates within one day (50 µg/mL), while inter-day assay was analysed using the same concentration for intra-day precision on four different days (interval of 1 day). The repeatability test of the sample was performed on 7-fold experiments of the mixture solution contain 16 reference standards (100 µg/mL). The stability test was performed with one sample solution, which was stored at room temperature and analysed at regular intervals (0, 2, 4, 8, 12, 24 and 48 h.). For analysis of recovery data, the appropriate amounts of the powdered sample of 16 reference standards were weighted and spiked with a known amount of reference compound and then analysed five times.

### 2.6. Organoleptic Analysis and Crude Composition Analysis

Organoleptic parameters (colour, odour, taste) of *A. frigida* herbal tea was determined according to AHPA guidance on Organoleptic Analysis [25]. Extractives and ash were determined according to WHO recommendations [26]. The protein content was estimated by Bradford method using BSA as a reference substance [27]. The lipid content was determined by extracting a known volume of *A. frigida* herbal tea with chloroform–methanol mixture (4:1). Carbohydrate content was determined with spectrophotometric phenol–sulphuric acid method [28]. Free sugars, organic acids, amino acids and mineral content were determined using HPLC-UV assays described previously [29]. Macronutrients, free sugars, organic acids and amino acids content were expressed as mg per 100 mL of the beverage and mineral content as μg per 100 mL of the beverage.

### 2.7. General Phytochemical Analysis

Essential oil content was determined gravimetrically after hydrodistillation in Clevenger apparatus [30]. Spectrophotometric assays were used to determine total content of flavonoids (as cynaroside equivalents) [31], caffeoylquinic acid (as 3-*O*-caffeoylquinic acid equivalents) [32], coumarins (as umbelliferone equivalents) [33], anthocyanidins (as cyanidin-3-*O*-glycoside equivalents) [23], tannins (as pentagalloylglucose equivalents) [34], iridoids (as loganic acid equivalents) [35], water-soluble polysaccharides (as glucose equivalents) [36], and alkaloids (as stachydrine equivalents) [37]. All quantitative phytochemical data were expressed as mg per 100 mL of the beverage. 

### 2.8. Antioxidant Activity Analysis

#### 2.8.1. DPPH^•^ Radical Scavenging Assay

The DPPH^•^ radical scavenging activity (DPPH^•^) was assessed as described earlier [38]. 500 μL of a DPPH^•^ methanol solution (freshly prepared, 100 μg/mL) was added to 500 μL of *A. frigida* herbal tea. After 15 min absorbance was measured at 520 nm. A 0.01% solution of trolox was used as a positive control (PC), and water was used as a negative control (NC). The ability to scavenge DPPH^•^ radicals was calculated using the following equation: Scavenging ability (%) = ((A_520_^NC^ − A_520_^PC^) − (A_520_^Sample^ − A_520_^PC^)/(A_520_^NC^ − A_520_^PC^)) × 100, where A_520_^NC^ is the absorbance of the negative control, A_520_^PC^ is the absorbance of the positive control, and A_520_^Sample^ is the absorbance of the sample solution. The IC_50_ value is the effective concentration at which DPPH^•^ radicals were scavenged by 50%. Values are expressed as mean obtained from five independent experiments.

#### 2.8.2. DPPH^•^-HPLC-DAD Procedure

The DPPH^•^-HPLC-DAD procedure was realized as described previously [27]. Briefly, a sample of *A. frigida* herbal tea (100 μL) was added to DPPH^•^ radical solution in methanol (250 μL, 20 mg/mL). The mixture was shaken for a few seconds and left to stand in the dark for 30 min at room temperature. Then, the sample was filtered through a 0.22 μm membrane filter. The untreated sample was prepared by adding a sample of *A. frigida* herbal tea (100 μL) to methanol (250 μL). HPLC analysis was performed as described in Section 2.3.

#### 2.8.3. Oxygen Radical Absorbance Capacity (ORAC) Assay

Lipophilic and hydrophilic oxygen radical absorbance capacity (L-ORAC, H-ORAC) were determined using fluorimetric microplate assay [39,40]. To prepare the sample, 100 mL of *A. frigida* herbal tea was extracted with 3 × 20 mL of hexane. The hexane fractions were combined and evaporated in vacuo (20 °C) to dryness. The residue was dissolved in 2.5 mL of acetone and then diluted with 7.5 mL of 7% randomly methylated β-cyclodextrin (RMCD; solution in acetone-water mixture 1:1, *v*/*v*) (L-ORAC sample). The residual *A. frigida* herbal tea after hexane treatment was extracted with 25 mL of acetone-water-acetic acid (70.0:29.5:0.5, *v*/*v*) by vortexing for 1 min and followed sonication for 5 min (40 °C). The sample was centrifuged at 4000× *g* for 15 min and organic supernatant was transferred to a volumetric flask (25 mL) and diluted to 25 mL with acetone (H-ORAC sample). An aliquot of L-ORAC or H-ORAC (40 μL) was to the 48-well plate, followed to 400 μL of fluorescein (0.3 mg/mL in 0.075 M phosphate buffer) and 150 μL of 2,2′-azobis(2-amidinopropane) (17.2 mg/mL in 0.075 M phosphate buffer), and readings of fluorimetric microplate reader (excitation wavelength 485 nm, emission wavelength 520 nm) were initiated immediately. The ORAC value was calculated using regression between trolox concentration (μM) and the net area under fluorescence decay curve. For the standard trolox assay, the dilution of trolox in 0.075 M phosphate buffer 6.25–50.00 μM was used. Data were expressed as trolox equivalents (μM) per 100 mL of the beverage. All the analyses were carried out in triplicate and the data were expressed as mean value ± standard deviation (SD).

### 2.9. Simulated Gastrointestinal Digestion Assay

The simulated gastrointestinal digestion was realized as described previously [41]. For the simulated gastric digestion phase, the sample of *A. frigida* herbal tea (25 mL) was incubated with freshly prepared simulated gastric fluid (25 mL, pH 2.0) in a 50 mL Erlenmeyer flask for 60 min at 37 °C in a shaking water bath (167 rpm). The gastric digestion phase was terminated by inactivating pepsin by raising the pH of the solution to 7.0 with the addition of 1 M NaOH. For the simulated intestinal digestion phase, after gastric digestion (pH 7.0) the whole sample was transferred to the dialysis bag used as the simulated small intestinal compartment. One mL of bile solution and 4 mL of simulated intestinal fluid were added to the dialysis bag, and digestion was continued for 4 h with continuous stirring. The dialysis bag was immersed in a vessel containing buffer solution (similar in composition to simulated intestinal fluid without pancreatin addiction, 1000 mL, pH 7.0) and maintained at 37 °C while mixing. This vessel was connected to a buffer feeding reservoir (at 37 °C) and a receiving flask. The buffer solution in which the dialysis bag was immersed was constantly replenished from the feeding reservoir at a transfer rate of 1.6 mL/min using a peristaltic pump. Samples (50 μL) were collected at 60 min of gastric digestion and at 240 min of intestinal digestion from retentate. HPLC samples were neutralized (if needed), and then freeze-dried. Samples were filtered through 0.2 μm syringe filters before injection into the HPLC system for analysis. HPLC-DAD conditions were similar to those in Section 2.3.

To prepare the simulated gastric fluid, 61.0 mL NaCl (200.0 g/L), 11.7 mL NaH_2_PO_4_ (88.8 g/L), 35.8 mL KCl (89.6 g/L), 70.0 mL CaCl_2_·2H_2_O (22.2 g/L), 39.0 mL NH_4_Cl (30.6 g/L), and 32.5 mL HCl (37%) were mixed in a volumetric flask and the total volume was adjusted to 250 mL by distilled water. Then the solution was supplemented by HCl up to pH 2.0 (solution I). The simulated gastric fluid was prepared before use by mixing pepsin (400 mg) with the 25 mL of solution I (stored at 4 °C). The simulated intestinal fluid was prepared after mixing 75.0 mL NaCl (200.0 g/L), 75.0 mL NaHCO_3_ (84.7 g/L), 19.0 mL KH_2_PO_4_ (8 g/L), 12.0 mL KCl (89.6 g/L), and 19.0 mL MgCl_2_ (5 g/L). The total volume was adjusted to 200 mL by distilled water (solution II). The simulated intestinal fluid was prepared before use by mixing pancreatin (40 mg) with 4 mL of solution II. The simulated bile solution consisted of bile (50 mg) dissolved in 10 mL of solution containing 2.93 mL NaCl (175.3 g/L), 6.65 mL NaHCO_3_ (84.7 g/L), 0.40 mL KCl (89.6 g/L), and 0.02 mL HCl (37%).

### 2.10. Statistical and Multivariative Analysis

Statistical analyses were performed using a one-way analysis of variance (ANOVA), and the significance of the mean difference was determined by Duncan’s multiple range test. Differences at *p* < 0.05 were considered statistically significant. The results are presented as mean values ± SD (standard deviations) of the three replicates. Advanced Grapher 2.2 (Alentum Software Inc., Ramat-Gan, Israel) was used to perform linear regression analysis and to generate graphs. Principal component analysis (PCA) based on a data matrix (16 markers × 21 samples) was performed using Graphs 2.0 utility for Microsoft Excel (Komi NTc URO RAN, Syktyvkar, Russia) to generate an overview for groups clustering.

## 3. Results and Discussion

### 3.1. Caffeoylquinic Acids and Flavonoids of Artemisia frigida Herb: HPLC-DAD-ESI-QQQ-MS Profile, Organ Distribution and Chemotaxonomy 

**Figure 2:**
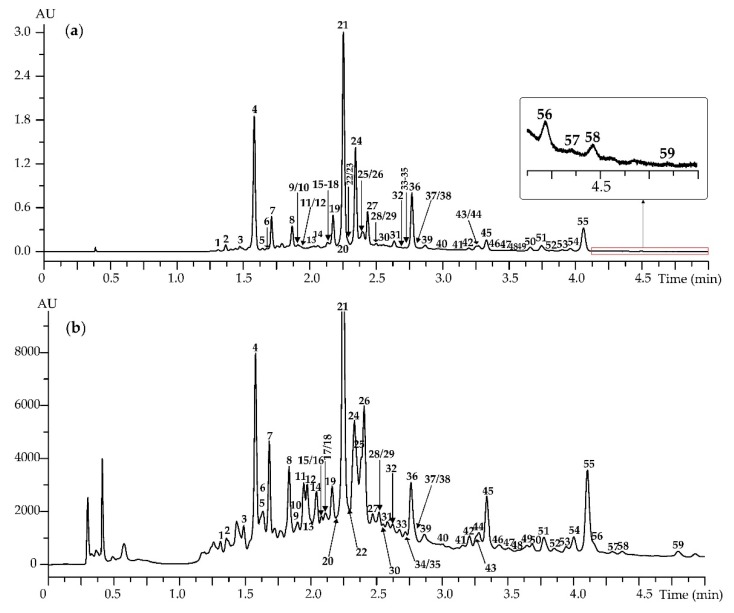
High-Performance Liquid Chromatography with (**a**) Diode Array Detection (HPLC-DAD) chromatogram (330 nm; on cut—enlarged fragment) and (**b**) High-Performance Liquid Chromatography with Electrospray Ionization Triple Quadrupole Mass Spectrometric Detection (HPLC-ESI-QQQ-MS) chromatogram (base peak chromatogram or BPC mode, negative ionization) of *A. frigida* herbal extract. Compounds are numbered as listed in Table 2.

The phenolic profile of *A. frigida* was investigated using HPLC with both diode array detection (DAD) and electrospray ionization triple quadrupole mass spectrometry (ESI-QQQ-MS) (Figure 2). Detected compounds were identified by their retention times (*t*_R_) and ultraviolet spectral (UV) and mass spectrometric data (Figure S1) by comparison with reference standards (Figure S2) and literature data. The method gave reproducible detection of 59 phenolics in *A. frigida* with various structures like caffeoylquinic acids, flavonoid glycosides and flavonoid aglycones (Table 2, Figure S3). Such a study of *A. frigida*, using HPLC-DAD-ESI-QQQ-MS, has not been realized previously. 

#### 3.1.1. Caffeoylquinic Acids

Twelve chromatographic peaks (1, 2, 4–6, 19, 21, 24, 26, 31, 32, 37) with a similar UV profile typical of caffeoyl derivatives (caffeoylquinic acids) were detected in *A. frigida*. The charge on deprotonated ions allowed them to be identified as mono-*O*-caffeoylquinic acids with *m*/*z* 353 (1, 2, 4, 5), di-*O*-caffeoylquinic acids with *m*/*z* 515 (6, 19, 21, 24, 26) and tri-*O*-caffeoylquinic acids with *m*/*z* 677 (31, 32, 37). Compared with reference standards, 1-*O*- (1), 4-*O*- (2), 5-*O*- (4), 3-*O*-caffeoylquinic acids (5), 1,3-di-*O*- (6), 3,4-di-*O*- (19), 3,5-di-*O*- (21), 4,5-di-*O*- (24), 1,5-di-*O*-caffeoylquinic acids (26), 1,3,5-tri-*O*- (31), 1,4,5-tri-*O*- (32) and 3,4,5-tri-*O*-caffeoylquinic acids (37) were found. Caffeoylquinic acids are widely distributed in species of the genus *Artemisia* [21], but their presence in *A. frigida* has been demonstrated for the first time here. 

#### 3.1.2. Flavonoid Glycosides

Flavonoid glycosides were identified as derivatives of apigenin (7, 9, 11, 13–15, 29), luteolin (8, 10, 17, 27), chrysoeriol (12, 22, 28, 33, 37, 39), 6-hydroxyluteolin (16, 30, 38), nepetin (18, 23, 34) and others (3, 20, 25, 35, 40).

Seven apigenin derivatives were detected, and six of them were identified as the *C*-glycosides vicenin-2 (7), isoschaftoside (9), schaftoside (11), vitexin (13) and isovitexin (15) and the *O*-glycoside cosmosiin (29). Compound 14 was a mixed *C*-hexoside-*O*-hexoside of apigenin. The negative mass spectrum demonstrated a deprotonated ion (M–H)^−^ with *m*/*z* 593, as well as a dehexosylated fragment with *m*/*z* 431, and the fragments caused the removal of particles with *m*/*z* 90 and 120, characteristic of flavone *C*-glycosides [46]. Known *C*-hexosides-*O*-hexosides of apigenin common to *Artemisia* species [21], such as saponarin (apigenin-6-*C*-glucoside-7-*O*-glucoside) and isosaponarin (apigenin-6-*C*-glucoside-4′-*O*-glucoside), showed different chromatographic mobilities, therefore, the structure of the compound was not determined completely. Flavones 7, 9 and 11 were previously identified in *A. frigida* [20]. The presence of 13–15 and 29 was shown for the first time in the species.

The identified luteolin derivatives were two *C*-glycosides, isoorientin (8) and orientin (10), and two *O*-glycosides, cynaroside (17) and luteolin-4′-*O*-glucoside (27), but only 17 was previously detected in this species [13]. According to mass spectrometric (*m*/*z* 461 (M–H)^−^, 299 (M–H–glucose) ^−^) and UV spectroscopic data, three isomeric *O*-hexosides of chrysoeriol were revealed in *A. frigida*, including chrysoeriol-7-*O*-glucoside (thermopsoside; 22), chrysoeriol-4′-*O*-glucoside (33), and unknown chrysoeriol-*O*-hexoside 39. Only 33 was previously found in Chinese samples of *A. frigida* [15,16]. 

Compound 39 had close to 33 spectral values but a greater retention time (*t*_R_ 2.875 min versus 2.714). On the UV spectrum, a hypochromic shift of band II (λ_max_, nm 267→263) was observed, which is typical for 5-*O*-glucosides of flavones [45].

For chrysoeriol, only three *O*-monoglucosides (5-*O*-, 7-*O*-, 4′-*O*-) can exist, and the position of two of them (22, 33) was already defined. Therefore, component 39 may be tentatively identified as chrysoeriol-5-*O*-glucoside. Compounds 12, 28 and 37 were also isomeric hexosides of chrysoeriol, however, they contained an additional fragment of the acetyl group, as evidenced by the mass spectrum pattern indicating the sequential removal of the acyl group with a molecular mass of 42 a.m.u. (*m*/*z* 503→461) and the hexose fragment (*m*/*z* 461→299). The existence of several *O*-acetyl-hexosides of chrysoeriol, including 7-*O*-(6′′-*O*-acetyl)glucoside from *Sideritis lanata* L. (Lamiaceae) [47], 4′-*O*-(2′′-*O*-acetyl)glucoside (abutilin A) from *Abutilon pakistanicum* Jafri and Ali (Malvaceae) [48] and 4′-*O*-(6′′-*O*-acetyl) glucoside from *Onobrychis viciifolia* Scop. (Leguminosae) [43], is known. None of these compounds have been previously found in *A. frigida* and the genus *Artemisia* generally.

Derivatives of 6-hydroxyluteolin 16, 30 and 38 had typical mass spectral patterns (*m*/*z* 463, 301). Compounds 16 and 38 were the known flavone-*O*-glycosides identified by comparison with a standard sample as widely distributed glycoside 6-hydroxyluteolin-7-*O*-glucoside [22] and rare rhaunoside C (6-hydroxyluteolin-4′-*O*-glucoside) previously found only in *Rhaponticum uniflorum* (L.) DC. (Asteraceae) [22]. Glycoside 30 was isomeric to 16 and gave the hypochromic shift of band I (λ_max_ 345→339) on the UV spectrum typical of ring B-glycosylated derivatives [45]. Because of the known position of 6-hydroxyluteolin-4′-*O*-glucoside (38), there is only the possible substitution of 30, a previously unreported 3′-*O*-glucoside that can be considered as a new compound. 

A similar identification of nepetin derivatives revealed the presence of nepitrin (nepetin-7-*O*-glucoside; 18) and its two ring B isomers nepetin-3′-*O*-glucoside or rhaunoside F (23) and nepetin-4′-*O*-glucoside (34). The latter flavone was first isolated from *Cirsium oligophyllum* (Franch. and Sav.) Matsum (Asteraceae) [49] can be found in other Asteraceous species, but the glycoside 23 was discovered earlier only in *Rhaponticum uniflorum* [22]. 

Flavone glycoside 3, which gave a deprotonated ion with *m*/*z* 695, was the only flavonoid that showed the loss of hexuronic acid residues in its mass spectrum (*m*/*z* 695→519, 519→343). The fragmentation character and UV spectrum indicated that the most likely identity of the compound was pentahydroxyflavone trimethyl ester di-*O*-hexuronide. The flavone with the closest structure found in *A. frigida* is 5,7-dihydroxy-3′,4′,5′-trimethoxyflavone-7-*O*-(2′′-*O*-glucuronyl)glucuronide or friginoside B [15,16]. Diosmetin-7-*O*-glucoside (20), identified after comparison of t_R_, UV, and ESI-MS data with the reference standard, was found in *A. frigida* for the first time. Three isomeric flavones 25, 35 and 40 were luteolin-methyl ester-*O*-hexoside due to their UV and ESI-MS patterns [14]. Their structures await further investigation. 

#### 3.1.3. Flavonoid Aglycones

Nineteen flavonoid aglycones (41–59) as flavone and flavonol derivatives were found, and 12 substances were identified according to their chromatographic behaviour and UV and mass spectra, in comparison with reference standards (Figure 2, Table 2). As a result, the known *A. frigida* aglycoses tricin (41), apigenin (42), hispidulin (43) [4], jaceosidine (45) [13,20], chrysoeriol (47) [13], luteolin-3′,4′-dimethyl ether (50) [4], and eupatorin (51) [15,16,18] were detected. Acacetin (54), cirsimaritin (55), cirsilineol (56), velutin (57) and genkwanin (59) were found for the first time in *A. frigida*.

Compounds 44 and 48 had similar mass spectral patterns with deprotonated ion *m*/*z* 359 and a series of fragments caused by the loss of three methoxyl groups with *m*/*z* 345, 331 and 317. The difference in retention times and absorption spectra allowed them to be tentatively identified as 5,7,3′-trihydroxy-6,4′,5′-trimethoxyflavone (44) and 5,7,4′-trihydroxy-6,3′,5′-trimethoxyflavone (48), previously detected in a North American population of *A. frigida* [12]. Compound 46 was characterized as a dimethoxylated heptahydroxyflavone, and its structure was determined as 5,7,3′,4′,5′-pentahydroxy-6,8-dimethoxyflavone based on UV and mass spectral data [17]. Previously, 46 was isolated from *A. frigida* only in the form of 7-*O*-glucuronide-4′-*O*-glucoside, named friginoside E [17], therefore, this aglycone was found in plants for the first time. Compound 49 has UV and mass spectra typical of dimethoxylated flavones (*m*/*z* 329, 315, 301) [15], which allowed its structure to be identified as 5,7,3′-trihydroxy-6,4′-dimethoxyflavone, the known flavone of *A. frigida* [15,16,18]. Flavone 5,7,3′,4′-tetrahydroxy-6,5′-dimethoxyflavone (52; *m*/*z* 345 (M–H)^−^) and flavonol quercetagetin-3,6,3′,4′-tetramethyl ether (53; *m*/*z* 373 (M–H)^−^) discovered in the chloroform fraction were also previously found in *A. frigida* [13]. Compound 58 gave a pseudo-molecular ion with *m*/*z* 313 and demethoxylated fragments with *m*/*z* 299 and 285. Taking into account the UV spectral data (λ_max_ 251, 275, 340 nm), 58 was identified as pillion (5,3′-dihydroxy-7,4′-dimethoxyflavone) [50] previously detected in *A. gmelinii* Weber ex Stechm (*A. iwayomogi* Kitam.) [51].

Finally, 59 phenolics were detected in *A. frigida*, of which 41 compounds were identified for the first time in plants. The presence of phenolic compounds in *A. frigida* was demonstrated earlier [12,13,14,15,16,17,18,19,20], but this report showed the greater diversity of caffeoylquinic acids, flavonoid glycosides and aglycones, which are possible bioactive compounds.

#### 3.1.4. Organ Distribution of Caffeoylquinic Acids and Flavonoids in *A. frigida* Plant

Analysing the HPLC chromatograms of total extracts of different *A. frigida* organs (leaves, flowers, stems and roots), we found the same profiles of leaf and flower extracts including compounds 1–59 (Figure S4). The profile of stem extract was sufficiently similar but contained only 23 compounds (2–4, 7–10, 19, 21, 24–27, 31, 32, 36, 43–45, 50, 51, 54, 55), and root extract was the poorest in phenolics, with 13 compounds (2–4, 6–8, 13, 14, 19, 21, 24, 27, 36). The same character of phenolic distribution was already reported for other *Artemisia* plants such as *A. annua* L. [52] and *A. campestris* subsp. *maritima* (DC.) Arcang. [53]. Although data on organ-specific distribution of caffeoylquinic acids and flavonoids in *Artemisia* plants has been limited, it can be argued that phenolic compounds accumulate in flowers and leaves and, to a lesser extent, in roots.

#### 3.1.5. Chemotaxonomic Significance of Flavonoids and Caffeoylquinic Acids in the Subsection Frigidae of *Artemisia* Genus

The *Artemisia* species of the Frigidae subsection (Absinthium section), including *A. frigida,* accumulate flavones of various structural types. Derivatives with 5,7,3′ and 5,7,3′,4′ substitutions (apigenin, luteolin, chrysoeriol) were found in *A. alpina* Pall. ex Willd. (*A. caucasica* Willd.) [54], *A. austriaca* Jacq. [55], *A. sericea* Weber ex Stechm. [21] and *A. xerophytica* Krasch. [56]. Flavones with an additional 6-hydroxy group (5,6,7,3′ or 5,6,7,3′,4′) detected in the form of di- and trimethoxy-substituted derivatives (eupatilin, eupafolin, jaceosidine, cirsimaritin, cirsilineol) were typical for *A. austriaca* [55,57] and *A. xerophytica* [56]. Glycosylation in the *O*-position at C-7 was the predominant form of aglycone binding to sugars in the Frigidae subsection, and 7-*O*-glucosides of apigenin (39) and luteolin (31) [21,54,55,56] were the most common glycosides. *C*-glycosides were previously detected only in *A. frigida* [20] and *A. sericea* [21], and caffeoylquinic acids were revealed only in *A. sericea* [21]. The known literature data and the data obtained in this study allowed us to conclude that the main chemical features of the flavone skeleton in the Frigidae subsection are two or three hydroxylated A rings at the 5,7- and/or 5,6,7-position and one or two hydroxylated B rings at the 4′- and/or 3′,4′-position. One, two or three methoxylated derivates of flavones at positions C-6, C-7, C-3′ and/or C-4′ are the usual components of the flavonoid profile in the Frigidae subsection. 

Flavonol aglycones and glycosides common to other sections of the genus Artemisia [58] are trace or non-detectable components in the Absinthium section. The chemosystematic significance of caffeoylquinic acids is currently not obvious, since they are probably obligatory components of the genus *Artemisia* generally [21].

### 3.2. Variation of Phenolics in A. frigida Herb: HPLC Quantification and Principal Component Analysis Data of Twenty One Siberian Populations

The known HPLC-DAD method of caffeoylquinic acid and flavonoid separation of *Artemisia* components [21] was used for quantitative analysis of *A. frigida* herbs. Sixteen non-trace compounds were selected as quantifiable markers, including six caffeoylquinic acids (4-*O*-; 5-*O*-; 3,4-di-*O*-; 3,5-di-*O*-; 4,5-di-*O*-; 3,4,5-tri-*O*-caffeoylquinic acid), three flavone glycosides (vicenin-2; isoorientin; cynaroside), and seven flavonoid aglycones (apigenin; hispidulin; jaceosidin; luteolin-3′,4′-dimethyl ester; eupatorin; acacetin; cirsimaritin) (Figure S5). Validation analysis of reference standards mixture demonstrated good linearity with correlation coefficient 0.9999 (Table S3, Figure S6). LOD (0.12–0.96 µg/mL) and LOQ (0.36–2.89 µg/mL) values were appropriate for quantitative analysis. Values of intra- and inter-day precision were 1.23–2.44% and 1.09–2.31%, respectively (Table S4). The repeatability of the method was 1.12–2.44% and the stability values varied from 1.44% to 2.37%.

**Figure 3:**
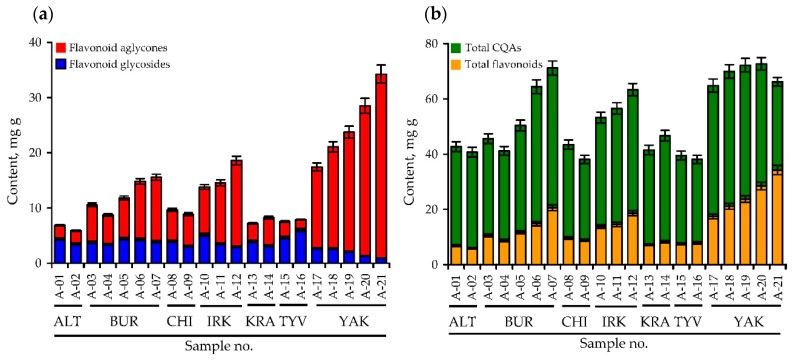
Histograms of the accumulative content of (**a**) flavonoid aglycones and flavonoid glycosides and (**b**) total flavonoids and caffeoylquinic acids (CQAs) in the herb of 21 samples of *A. frigida* (A-01–A-21). Siberian regions: ALT—Altai Krai, BUR—Buryatia Republic, CHI—Chita Oblast, IRK—Irkutsk Oblast, KRA—Krasnoyarskii Krai, TYV—Tyva Republic, YAK—Yakutia (Sakha) Republic.

Analysing the HPLC profiles of herbal extracts of *A. frigida* from the seven Siberian regions and 21 populations, we found that the variation in the total concentrations of phenolics, caffeoylquinic acids, flavonoid glycosides and flavonoid aglycones in *A. frigida* were 38.07–72.69 mg/g, 29.25–50.63 mg/g, 0.92–4.63 mg/g and 1.87–33.32 mg/g, respectively (Figure 3; Table S5). Two basic caffeoylquinic acids of *A. frigida* herbs, 5-*O*-caffeoylquinic acid and 3.5-di-*O*-caffeoylquinic acid, showed concentrations of 2.11–17.22 mg/g and 10.08–24.55 mg/g. The predominant flavonoid glycosides were vicenin-2 (0.10–2.39 mg/g) and isoorientin (0.70–3.54 mg/g), as well as jaceosidine (0.51–6.69 mg/g) and cirsimaritin (0.34–17.71 mg/g). Known data about phenolic levels in herbs of *A. frigida* refers only to flavonoid content and are available only for Chinese populations where the highest concentrations of /*O*-glycoside (12.97 mg/g), diosmetin (6.81 mg/g), desmethylcentaureidin (4.40 mg/g) and eupatorin (3.83 mg/g) were shown [15,16,19]. The concentrations of these flavonoids were low or undetectable in Siberian populations of *A. frigida*, possibly demonstrating the existence of various chemical races of *A. frigida* on the Asian territory.

**Figure 4:**
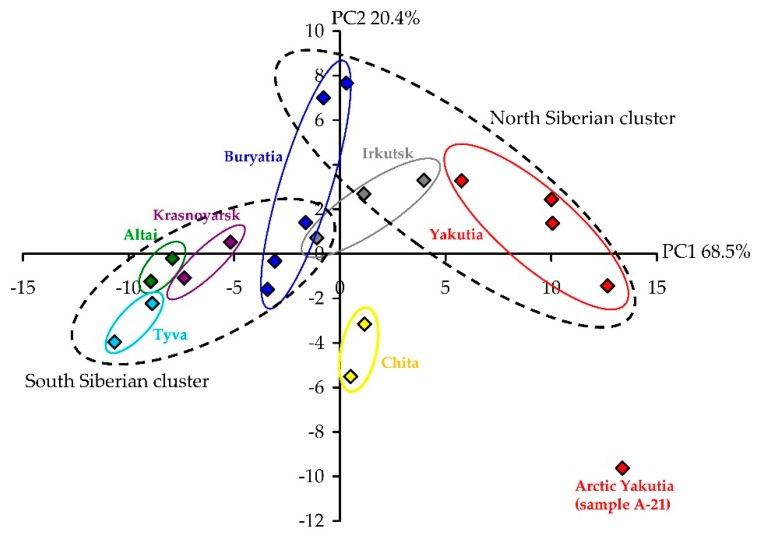
Results of principal component analysis (PCA) used the content of flavonoids and caffeoylquinic acids in the herb of 21 samples of *A. frigida*.

Moreover, western Siberian populations (Altai Krai, Krasnoyarsk Krai, Tyva Republic) with a moderate continental climate were less able to accumulate lipophilic flavone aglycones. Harsh continental climate populations of Eastern Siberia located in Baikal regions (Buryatia Republic, Irkutsk Oblast, Chita Oblast) contained intermediate levels of quantifiable components. The highest content of lipophilic flavonoid aglycones and the lowest content of monocaffeoylquinic acids flavone glycosides were in northern populations of *A. frigida* grown in the Yakutia Republic, which has a harsh continental and subarctic climate. These observations, illustrated well by the results of principal component analysis (PCA), allowed us to observe the formation of three distant clusters including populations of Altai Krai, Krasnoyarsk Krai, Tyva Republic (cluster I), Buryatia Republic, Irkutsk Oblast, Chita Oblast (cluster II) and Yakutia Republic (cluster III) (Figure 4). A possible reason for the differences between *A. frigida* populations could be the value of the climate continentality or extremeness. Due to the very large area of the Siberian territory, there are various ecological regions located there, resulting in variation in the chemical profiles of plants. Phenolic compounds are the early chemical markers reflecting plant relationships with the environment, and similar changes in the phenolic profile have already been demonstrated for some cultivated plants [59] and wild species [28,38,60,61,62]. These changes had to be taken into consideration due to their direct impact on bioactivity [63]. 

### 3.3. A. frigida Herbal Tea Phenolics: General Characteristics, HPLC Profile, in vitro Digestion Stability and Antioxidant Capacity

Tea made from *A. frigida* is an herbal beverage with a specific camphoraceous, eucaluptus-like, fresh and balsamic aroma, with a slightly bitter and pleasant taste. The concentrations of macronutrients, free sugars, organic acids, amino acids and minerals have valid values and were appropriate for herbal teas [27,36] (Table S6). The sensory evaluation showed that *A. frigida* herbal tea has good levels of colour, flavour and overall preferences, demonstrating its potential as a new herbal tea (Tables S7 and S8, Figure S7). The basic phytochemical components of *A. frigida* herbal tea were phenolic compounds like phenylpropanoids (42.18 mg/100 mL) and flavonoids (4.63 mg/100 mL) as well as water-soluble polysaccharides (24.11 mg/100 mL) (Table S9). Coumarins, anthocyanidins, tannins, iridoids, essential oils and alkaloids were the trace or undetectable components of *A. frigida* herbal tea.

The phenolic profile of *A. frigida* herbal tea included nineteen compounds predominantly hydrophilic in nature, such as caffeoylquinic acids (1, 2, 4, 19, 21, 24, 26, 21, 26), flavonoid glycosides (7–9, 13, 14, 27, 29) and flavonoid aglycones (45, 51, 55) (Figure S8). Most lipophilic components were not extracted due to the hydrophilic nature of tea extractant, water. For the same reason, the quantifiable compounds in sagaan aya tea were six caffeoylquinic acids (4-*O*-; 5-*O*-; 3,4-di-*O*-; 3,5-di-*O*-; 4,5-di-*O*-; 3,4,5-tri-*O*-caffeoylquinic acid) and two flavonoid glycosides (vicenin-2; isoorientin) (Table 3). 

The total content of caffeoylquinic acids and flavonoid glycosides in *A. frigida* herbal tea was 36.58 and 3.75 mg per 100 mL. Flavonoid aglycones were present in trace or undetectable concentrations. The two dominant phenolics of *A. frigida* herbal tea, 5-*O*-caffeoylquinic acid and 3,5-di-*O*-caffeoylquinic acid, were present at levels of 16.09 and 16.35 mg/100 mL of decoction, respectively. Together, these two compounds accounted for more than 88% of the total caffeoylquinic acid content and about 80% of the total phenolic content. A high caffeoylquinic acid content was previously found in decoctions of other *Artemisia* plants, such as *A. capillaris* Thunb., which had 15.72–27.69 mg/100 mL of 5-*O*-caffeoylquinic acid and 4.84–10.18 mg/100 mL of 3,5-di-*O*-caffeoylquinic acid [64], and *A. campestris* subsp. *maritima,* demonstrating a total caffeoylquinic acid content of 22.10 mg/100 mL [53]. At present, the known plant source of phenolic compounds in the everyday diet is coffee: the levels of total caffeoylquinic acids in soluble and instant coffee beverages range from 14.7 to 44.0 mg/100 mL [65]. We therefore conclude that *A. frigida* herbal tea solution can also be a good source of caffeoylquinic acids.

**Figure 5:**
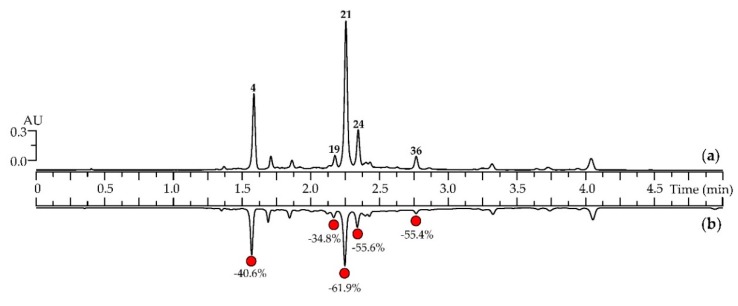
HPLC-DAD chromatograms (330 nm) of *A. frigida* herbal tea (**a**) before and (**b**) after prechromatographic reaction with DPPH^•^ radicals. Compounds are numbered as listed in Table 1. In (**b**) red circles show the compounds with the highest scavenging capacity, and numbers demonstrate the percentage peak area decrease compared with the peak area in (**a**).

Caffeoylquinic acids are known powerful antioxidants with a wide range of activity [66]. Plant sources of caffeoylquinic acids, such as various *Artemisia* plant extracts, always provide a high level of antioxidant protection [21]. In order to confirm the antioxidant potential of *A. frigida* herbal tea, we used an HPLC-based bioactivity profiling assay consisting of a prechromatographic reaction of the plant sample with 2,2-diphenyl-1-picrylhydrazyl free radical (DPPH^•^) (Figure 5). The resulting two-dimensional chromatogram demonstrated the high radical scavenging potency of the selected compounds as peaks of reduced area. 

Five components were the most active: 5-*O*-caffeoylquinic acid (peak 4), 3,4-di-*O*-caffeoylquinic acid (peak 19), 3,5-di-*O*-caffeoylquinic acid (peak 21), 4,5-di-*O*-caffeoylquinic acid (peak 24), and 3,4,5-tri-*O*-caffeoylquinic acid (peak 36), and the value of peak area reduction varied from 34.8% to 61.9%. Flavonoid glycosides and aglycones had little or no activity. The value of IC_50_ in the DPPH scavenging assay was 12.63 ± 0.51 μg/mL (versus 9.27 ± 0.27 μg/mL for Trolox as the reference compound), which confirmed the high antioxidant activity of *A. frigida* herbal tea. 

Of particular interest are the changes in phenolics that occur during the process of digestion carried out in different digestion media with various compositions, enzyme contents and pH levels [67]. Phenolic components of herbal beverages are generally considered to be stable in gastric and intestinal juices, but caffeoylquinic acids are known to be less resistant than flavonoid compounds [68]. Both classes of phytocomponents are known scavengers of free radicals [63], making it possible to use caffeoylquinic acids and flavonoids and their source, *Artemisia* plants, as prospective antioxidant agents [69]. There are no available data on the bioactivity of *A. frigida* and its preparations in connection with the digestive process transformation, so we studied the chemical and bioactivity changes that occurred in *A. frigida* herbal tea during in vitro digestion.

HPLC data on *A. frigida* herbal tea after gastric and intestinal media treatment demonstrated a 10.6% (32.67 mg/100 mL) and 35.2% (23.69 mg/100 mL) reduction of total caffeoylquinic acid content respectively, from initial levels (Table 3). Flavonoid glycosides were more stable, as their concentrations decreased by 1.6–10.7%. Despite this reduction, however, the total phenolic content in *A. frigida* herbal tea after the intestinal phase of digestion was still high (27.04 mg/100 mL).

**Figure 6:**
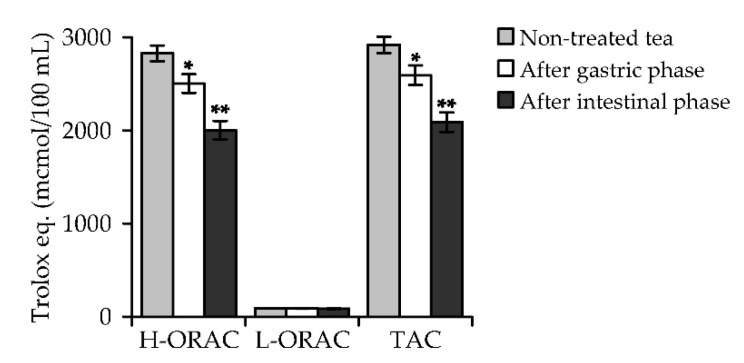
Capacity of hydrophilic (H-ORAC) and lipophilic (L-ORAC) antioxidants and total antioxidant capacity (TAC; as Trolox equivalents, μmol per 100 mL) of *A. frigida* herbal tea before and after in vitro treatment with simulated gastric and intestinal media. Results show mean ± SEM of four experiments performed in triplicate. * *p* < 0.01, ** *p* < 0.001 relative to the non-treated sample.

The total antioxidant capacity (TAC) of non-treated *A. frigida* herbal tea calculated as the sum of the capacities of hydrophilic (H-ORAC) and lipophilic (L-ORAC) antioxidants was 2918.77 μmol Trolox equivalents (TE) per 100 mL of beverage (Figure 6). The capacity of hydrophilic antioxidants (2826.14 μmol TE/100 mL) was higher than that of lipophilic antioxidants (92.63 μmol TE/100 mL) due to the hydrophilic nature of water as the extractant. The process of digestive media treatment resulted in a reduction of the content of antioxidant phenolics and finally to a decrease in antioxidant capacity.

The index of TAC of gastric medium-treated *A. frigida* herbal tea was 11% less (2593.56 μmol TE/100 mL) than that of the untreated sample, but intestinal medium treatment resulted in a further decrease of up to 2090.14 μmol TE/100 mL. The reduction of hydrophilic antioxidant content in the beverage after digestive media treatment played a key role in the loss of general antioxidant activity. Despite the reducing effect of digestion phases on the phenolic content and activity of *A. frigida* herbal tea, the final levels of antioxidant activity remained high because of the appropriate content of bioactive components. Earlier data about in vitro digestion of *Artemisia gorgonum* Webb infusion also demonstrated a reduction of the phenolic compound content, but FRAP, CUPRAC and other antioxidant parameters were high after gastric and intestinal media treatment [70]. Despite the limited data on the digestive stability of *Artemisia*, one can assume that the antioxidant capacity of *Artemisia* extracts remains high because of their high hydroxycinnamate content. 

## 4. Conclusions

In this work, *Artemisia frigida* as a new herbal source of hydroxycinnamates and flavonoids was studied, and its phenolic metabolic profile was analysed, focusing on caffeoylquinic acids, flavonoid glycosides and aglycones. The wide spectrum of phenolics was characterized, most of them being newly found in *A. frigida* and the *Artemisia* genus. The HPLC profile data on selected phenolics in *A. frigida* of Siberia origin exhibited high variability in the chemical patterns of the species, which requires consideration of how it might be reflected in the biological activity and practical use of a raw material. The new herbal beverage, *A. frigida* tea, was also investigated chemically, and most of the phenolics therein demonstrated high stability after in vitro digestion. The high antioxidant capacity of *A. frigida* herbal tea before and after digestive media treatment makes it a good candidate for use as a prophylactic and therapeutic remedy for various redox imbalances. Satisfactory sensory attributes of *A. frigida* tea and global interest in functional herbal beverages opens up new commercial avenues for *Artemisia* tea.

## Figures and Tables

**Table 1:** Detailed information of Artemisia frigida samples.

No	Collection Place	Collection Date	Coordinates	Height (m a.s.l.)	Voucher Specimens No
A-01	Altaiskii Krai, Smolenskii District, Belokurikha	20.VII.2017	51°58′12.5′′N, 84°57′23.5′′E	448	BU/AK-As-ar/h-0717-027
A-02	Altaiskii Krai, Novichikhinskii District, Mel’nikovo	27.VII.2017	52°10′47.0′′N, 81°12′34.1′′E	230	BU/AK-As-ar/h-0717-032
A-03	Buryatia Republic, Mukhorshibirskii District, Mukhorshibir’	21.VII.2018	51°01′45.3′′N, 107°47′49.9′′E	765	BU/BR-As-ar/h-0718-094
A-04	Buryatia Republic, Zakamenskii District, Tsakir	19.VII.2016	50°24′59.2′′N, 103°34′02.6′′E	1078	BU/BR-As-ar/h-0716-041
A-05	Buryatia Republic, Okinskii District, Orlik	22.VII.2017	52°29′35.3′′N, 99°47′08.2′′E	1857	BU/BR-As-ar/h-0717-026
A-06	Buryatia Republic, Bauntovskii District, Malovskii	29.VII.2015	54°22′45.1′′N, 113°32′16.3′′E	950	BU/BR-As-ar/h-0715-116
A-07	Buryatia Republic, Severobaikal’skii District, Kumora	31.VII.2017	55°52′02.7′′N, 111°10′58.4′′E	578	BU/BR-As-ar/h-0717-094
A-08	Chita Oblast, Akshinskii District, Ureisk	21.VII.2017	50°16′59.0′′N, 113°10′42.5′′E	902	BU/CO-As-ar/h-0717-012
A-09	Chita Oblast, Alexandrovo Zavodskii District, Klichka	24.VII.2017	50°28′07.9′′N, 118°06′30.0′′E	1085	BU/CO-As-ar/h-0717-029
A-10	Irkutsk Oblast, Shelokhovskii District, Olkha	18.VII.2017	52°09′10.4′′N, 104°06′46.2′′E	490	BU/IO-As-ar/h-0717-006
A-11	Irkutsk Oblast, Bratskii District, Vikhorevka	24.VII.2016	56°08′32.7′′N, 101°12′32.3′′E	381	BU/IO-As-ar/h-0716-011
A-12	Irkutsk Oblast, Katangskii District, Erbogachen	28.VII.2016	61°15′50.3′′N 108°00′50.5′′E	301	BU/IO-As-ar/h-0716-027
A-13	Krasnoyarskii Krai, Berezovskii District, Lopatino	17.VII.2015	55°56′39.1′′N, 93°17′33.1′′E	375	BU/KK-As-ar/h-0715-006
A-14	Krasnoyarskii Krai, Turukhanskii District, Kellog	22.VII.2015	62°26′38.5′′N, 86°18′16.1′′E	77	BU/KK-As-ar/h-0715-014
A-15	Tyva Republic, Kaa-Khemskii District, Saryg-Sep	25.VII.2018	51°28′48.5′′N, 95°29′26.5′′E	778	BU/TR-As-ar/h-0718-012
A-16	Tyva Republic, Barun-Khemchikskii District, Ak-Dovurak	27.VII.2018	51°08′28.0′′N, 90°37′38.1′′E	853	BU/TR-As-ar/h-0718-018
A-17	Yakutia (Sakha) Republic, Mirninskii Ulus, Almaznyi	25.VII.2017	62°28′01.9′′N, 113°50′58.1′′E	388	BU/YR-As-ar/h-0717-063
A-18	Yakutia (Sakha) Republic, Oimyakonskii Ulus, Ust’-Nera	30.VII.2017	64°31′50.4′′N, 142°59′42.1′′E	506	BU/YR-As-ar/h-0717-085
A-19	Yakutia (Sakha) Republic, Verkhekolymskii Ulus, Ugol’noye	1.VIII.2017	65°43′40.7′′N, 149°45′19.4′′E	134	BU/YR-As-ar/h-0817-106
A-20	Yakutia (Sakha) Republic, Srednekolymskii Ulus, Sylgy-Ytar	21.VII.2018	67°50′00.4′′N, 154°48′01.6′′E	31	BU/YR-As-ar/h-0718-064
A-21	Yakutia (Sakha) Republic, Nizhnekolymskii Ulus, Tymkino	27.VII.2018	68°28′46.8′′N, 160°07′56.7′′E	9	BU/YR-As-ar/h-0718-072

**Table 2:** Chromatographic (*t*_R_), ultraviolet data (UV), collision energy (CE) and mass-spectrometric data (ESI-MS/MS) of compounds 1–59 found in *A. frigida* herb.

No	*t*_R_, min	Compound	UV, λ_max_, nm	CE, eV	ESI-MS/MS, *m*/*z*		Ref.
					(M–H) ^−^	MS/MS Fragment Ions	
1	1.285	1-*O*-Caffeoylquinic acid ^a^	327	30	353	(353): 191, 179, 135	[42]
2	1.376	4-*O*-Caffeoylquinic acid ^a^	327	30	353	(353): 191, 179, 135	[42]
3	1.485	Friginoside B (Tent.) ^b^	270, 352	15	695	(695): 519, 343; [343]: 329, 301	[15]
4	1.614	5-*O*-Caffeoylquinic acid ^a^	327	30	353	(353): 191, 179, 135	[42]
5	1.627	3-*O*-Caffeoylquinic acid ^a^	327	30	353	(353): 191, 179, 135	[42]
6	1.687	1,3-Di-*O*-caffeoylquinic acid ^a^	328	35	515	(515): 353, 335, 191, 179	[42]
7	1.742	Vicenin-2 (Api-6,8-di-*C*-Glc) ^a^	255, 329	30	593	(593): 503, 473, 413; (473): 383, 353	[43]
8	1.871	Isoorientin (Lut-6-*C*-Glc) ^a^	255, 267, 348	25	447	(447): 357, 327	[44]
9	1.886	Isoschaftoside (Api-6-*C*-Ara-8-*C*-Glc) ^a^	273, 329	30	563	(563): 503, 473, 443, 413, 383, 353; (353): 325, 297	[44]
10	1.897	Orientin (Lut-8-*C*-Glc) ^a^	255, 267, 348	25	447	(447): 357, 327	[44]
11	1.938	Schaftoside (Api-6-*C*-Glc-8-*C*-Ara) ^a^	273, 329	30	563	(563): 503, 473, 443, 413, 383, 353; (353): 325, 297	[44]
12	1.942	Chrysoeriol-*O*-Ac-Hex ^b^	269, 336	15	503	(503): 461, 299	[43]
13	2.043	Vitexin (Api-8-*C*-Glc) ^a^	272, 330	25	431	(431): 341, 311	[44]
14	2.063	Apigenin-*C*-Hex-*O*-Hex ^b^	273, 329	30	593	(593): 431, 341, 311	[44]
15	1.121	Isovitexin (Api-6-*C*-Glc) ^a^	272, 330	20	431	(431): 341, 311	[44]
16	1.124	6-Hydroxyluteolin-7-*O*-Glc ^a^	251, 280, 345	10	463	(463): 301	[22]
17	1.129	Cynaroside (Lut-7-*O*-Glc) ^a^	256, 265, 347	10	447	(447): 285	[44]
18	1.132	Nepitrin (Nep-7-*O*-Glc) ^a^	271, 345	20	477	(477): 315, 301	[22]
19	2.188	3,4-Di-*O*-caffeoylquinic acid ^a^	328	40	515	(515): 353, 335, 191	[42]
20	2.210	Diosmetin-7-*O*-Glc ^a^	251, 268, 345	15	461	(461): 299, 285	[22]
21	2.251	3,5-Di-*O*-caffeoylquinic acid ^a^	327	40	515	(515): 353, 191, 179, 135	[42]
22	2.312	Thermopsoside (Chr-7-*O*-Glc) ^a^	253, 267, 346	15	461	(461): 299, 285	[44]
23	3.316	Rhaunoside F (Nep-3′-*O*-Glc) ^a^	268, 339	10	477	(477): 315, 301	[22]
24	2.372	4,5-Di-*O*-caffeoylquinic acid ^a^	328	40	515	(515): 353, 179	[42]
25	2.378	6-Hydroxyluteolin-dimethyl ether-*O*-Hex ^b^	253, 267, 343	25	491	(491): 329, 301	[22]
26	2.380	1,5-Di-*O*-caffeoylquinic acid ^a^	328	35	515	(515): 353, 191, 179, 135	[42]
27	2.437	Luteolin-4′-*O*-Glc ^a^	267, 337	10	447	(447): 285	[44]
28	2.497	Chrysoeriol-*O*-Ac-Hex ^b^	269, 336	15	503	(503): 461, 299	[43]
29	2.504	Apigenin-7-*O*-glucoside (cosmosiin) ^a^	265, 334	10	431	(431): 269	[44]
30	2.562	6-Hydroxyluteolin-3′-*O*-Glc (Tent.) ^b^	275, 339	10	463	(463): 301	[22]
31	2.626	1,3,5-Tri-*O*-caffeoylquinic acid ^a^	326	45	677	(677): 515, 353; (515): 353, 191, 179, 135	[42]
32	2.688	1,4,5-Tri-*O*-caffeoylquinic acid ^a^	326	45	677	(677): 515, 353; (515): 353, 191, 179	[42]
33	2.714	Chrysoeriol-4′-*O*-Glc ^a^	268, 337	15	461	(461): 299, 285	[44]
34	2.718	Nepetin-4′-*O*-Glc ^a^	268, 341	25	477	(477): 315, 301	[22]
35	2.722	6-Hydroxyluteolin-dimethyl ether-*O*-Hex ^b^	253, 267, 344	25	491	(491): 329, 301	[22]
36	2.756	3,4,5-Tri-*O*-caffeoylquinic acid ^a^	326	48	677	(677): 515; (515): 353, 179	[42]
37	2.810	Chrysoeriol-*O*-Ac-Hex ^b^	269, 336	15	503	(503): 461, 299	[43]
38	2.819	Rhaunoside C (6-hydroxyluteolin-4′-*O*-Glc) ^a^	286, 335	10	463	(463): 301	[22]
39	2.875	Chrysoeriol-5-*O*-Glc (Tent.) ^b^	261, 343	15	461	(461): 299, 285	[45]
40	2.934	6-Hydroxyluteolin-dimethyl ether-*O*-Hex ^b^	252, 267, 342	25	491	(491): 329, 301	[22]
41	3.120	Tricin ^a^	270, 345	35	329	(329): 315, 301	[44]
42	3.236	Apigenin ^a^	267, 334	10	269		[44]
43	3.251	Hispidulin ^a^	273, 333	10	299	(299): 285	[44]
44	3.259	5,7,3′-Trihydroxy-6,4′,5′-trimethoxyflavone ^b^	273, 331	35	359	(359): 345, 331, 317	[12]
45	3.312	Jaceosidine ^a^	273, 343	35	329	(329): 315, 301	[44]
46	3.375	5,7,3′,4′,5′-Pentahydroxy-6,8-dimethoxyflavone ^b^	269, 370	40	361	(361): 347, 333	[17]
47	3.482	Chrysoeriol ^a^	252, 272, 345	35	299	(299): 285	[44]
48	3.562	5,7,4′-Trihydroxy-6,3′,5′-trimethoxyflavone ^b^	272, 347	35	359	(359): 345, 331, 317	[12]
49	3.621	Desmethylcentaureidin ^b^	252, 273, 346	35	329	(329): 315, 301	[44]
50	3.638	Luteolin-3′,4′-dimethyl ether ^a^	251, 271, 342	40	313	(313): 299, 285	[44]
51	3.752	Eupatorin ^a^	252, 275, 343	40	343	(343): 329, 315, 301	[44]
52	3.812	5,7,3′,4′-Tetrahydroxy-6,5′-dimethoxyflavone ^b^	272, 350	40	345	(345): 331, 317	[13]
53	3.879	Quercetagetin-3,6,3′,4′-tetramethyl ether ^b^	256, 272, 344	40	373	(373): 359, 345, 331	[44]
54	3.894	Acacetin ^a^	270, 327	20	283	(283): 269	[44]
55	4.062	Cirsimaritin ^a^	274, 332	35	313	(313): 299, 285	[44]
56	4.124	Cirsilineol ^a^	273, 345	35	343	(343): 329, 315, 301	[44]
57	4.187	Velutin ^a^	251, 344	35	313	(313): 299, 285	[44]
58	4.251	Pilloin ^b^	251, 275, 340	35	313	(313): 299, 285	[44]
59	4.782	Genkwanin ^a^	268, 335	20	283	(283): 269	[44]

^a^ Compound identification was based on comparison with reference standard. ^b^ Compound identification was based on interpretation of UV and MS spectral data and comparison with literature data. Abbreviation used: Ac—acetyl, Api—apigenin, Ara—arabinosyl, Chr—chrysoeriol, Glc—glucosyl, Hex—hexosyl, Lut—luteolin, Nep—nepetin; Tent—tentatively; CE—collision energy.

**Table 3:** Content of selected caffeoylquinic acids and flavonoids in *A. frigida* herbal tea before and after in vitro treatment by the simulated gastric and intestinal media (mg/100 mL).

Compounds	*A. frigida* Herbal Tea, mg/100 mL		
	Non-Treated	After Gastric Phase	After Intestinal Phase
Caffeoylquinic acids			
4-*O*-Caffeoylquinic acid	0.45 ± 0.01	0.44 ± 0.01	0.39 ± 0.01
5-*O*-Caffeoylquinic acid	16.09 ± 0.45	15.12 ± 0.42	12.98 ± 0.33
3,4-Di-*O*-caffeoylquinic acid	0.82 ± 0.02	0.76 ± 0.02	0.57 ± 0.02
3,5-Di-*O*-caffeoylquinic acid	16.35 ± 0.43	14.02 ± 0.35	8.46 ± 0.22
4,5-Di-*O*-caffeoylquinic acid	2.87 ± 0.08	2.33 ± 0.06	1.29 ± 0.03
3,4,5-Tri-*O*-caffeoylquinic acid	*t*r.	*t*r.	*t*r.
Subtotal caffeoylquinic acids	36.58	32.67	23.69
Flavonoid glycosides			
Vicenin-2	1.34 ± 0.04	1.32 ± 0.04	1.31 ± 0.03
Isoorientin	2.41 ± 0.06	2.37 ± 0.06	2.04 ± 0.06
Cynaroside	*t*r.	*t*r.	*t*r.
Subtotal flavonoid glycosides	3.75	3.69	3.35
Flavonoid aglycones			
Apigenin	n.d.	n.d.	n.d.
Hispidulin	n.d.	n.d.	n.d.
Jaceosidine	*t*r.	*t*r.	*t*r.
Luteolin-3′,4′-dimethyl ether	n.d.	n.d.	n.d.
Eupatorin	n.d.	n.d.	n.d.
Acacetin	n.d.	n.d.	n.d.
Cirsimaritin	*t*r.	*t*r.	*t*r.
Subtotal flavonoid aglycones	*t*r.	*t*r.	*t*r.
Total flavonoids	3.75	3.69	3.35
Total phenolics	40.33	36.36	27.04

*t*r.—trace, n.d.—not detected.

## Supplementary Materials

The following are available online at https://www.mdpi.com/2076-3921/8/8/307/s1, Table S1. Ethnopharmacological use of *Artemisia frigida* (fringed sagewort) by the various nomadic people; Table S2. Phenolic compounds of *A. frigida* (literature data); Table S3. Regression equations, correlation coefficients (*r*^2^), standard deviation (*S*_YX_), limits of detection (LOD), limits of quantification (LOQ) and linear ranges for 16 compounds; Table S4. Intra- and inter-day precision, repeatability, stability and recovery for 16 compounds; Table S5. Content of selected caffeoylquinic acids and flavonoids in the 21 samples of *A. frigida* herb; Table S6. Macronutrients, free sugars, organic acids, amino acids and mineral composition of *A. frigida* herbal tea; Table S7. Demographic information of participants of tea sensory evaluation; Table S8. Sensory evaluation data of *A. frigida* herbal tea, green tea, black tea and *Artemisia absinthium* tea; Table S9. Phytochemical composition of *A. frigida* herbal tea; Figure S1. Mass spectra of compounds 1–59 found in *A. frigida*; Figure S2. Structures of reference standards used in present work; Figure S3. A series of three successive chromatograms of *A. frigida* extract demonstrating good reproducibility of the method used; Figure S4. HPLC-DAD chromatograms of *A. frigida* extract of leaves, flowers, stems and roots; Figure S5. HPLC-DAD chromatogram of the reference mixture of 16 pure compounds and internal standard; Figure S6. Calibration curves for 16 reference compounds used for quantitative HPLC-DAD assay; Figure S7. Sensory profiles of *A. frigida* herbal tea, green tea, black tea and *Artemisia absinthium* tea according to 30 participants estimation; Figure S8. HPLC-DAD chromatogram of *A. frigida* herbal tea.

## References

[1] Fabricant D.S., Farnsworth N.R. (2001). The value of plants used in traditional medicine for drug discovery. Environ. Health Perspect..

[2] Heinrich M., Liu H.W.B., Mander L. (2013). Ethnopharmacology and drug discovery. Comprehensive Natural Products II. Chemistry and Biology.

[3] Aziz-Ul-Ikram, Zahra N.B., Shinwari Z.K., Muhammad Q. (2015). Ethnomedicinal review of folklore medicinal plants belonging to family Apiaceae of Pakistan. Pak. J. Bot..

[4] Malyschev L.I. (2007). Flora of Siberia. Asteraceae (Compositae).

[5] Danzin P., Aseeva T.A. (2017). Perennial medicinal plants. Shel Pkxreng. Pure Crystal Necklace (Pharmacognosy of Tibetan Medicine).

[6] Sumati P., Aseeva T.A. (2008). Decoctions. Kunpan Dudzi. Useful for All Amrita Extract (Big Recipe Book of Aginsk Datsan).

[7] Desrid S.C., Aseeva T.A. (2014). Action of various medicinal remedies. Vaidur’ya Onbo. Blue Beryl Garland (Commentary to Bdud Rtsi, a Decoration of the Doctrine of the King of Medicine).

[8] Batorova S.M., Yakovlev G.P., Aseeva T.A. (2013). Reference-Book of Traditional Tibetan Medicine Herbs.

[9] Chirikova N.K. (2019). Phenolic and Terpenic Compounds of Plants from the Flora of Yakutia Republic. Ph.D. Thesis.

[10] Pizzino G., Irrera N., Cucinotta M., Pallio G., Mannino F., Arcoraci V., Squadrito F., Altavilla D., Bitto A. (2017). Oxidative stress: Harms and benefits for human health. Oxid. Med. Cell. Longev..

[11] Huyut Z., Beydemir S., Gülçin İ. (2017). Antioxidant and antiradical properties of selected flavonoids and phenolic compounds. Biochem. Res. Int..

[12] Liu Y.-L., Mabry T.J. (1981). Two methylated flavones from *Artemisia frigida*. Phytochemistry.

[13] Liu Y.-L., Mabry T.J. (1981). Flavonoids from *Artemisia frigida*. Phytochemistry.

[14] Wang Q.-H., Wang J.-H., Eerdunbagen, Tana (2009). Chemical constituents of *Artemisia frigida*. Chin. Trad. Herb. Drugs.

[15] Wang Q.-H., Ao W.-L.-J., Wang X.-L., Bao X.-H., Wang J.-H. (2010). Two new flavonoid glycosides from *Artemisia frigida* Willd. J. Asian Nat. Prod. Res..

[16] Wang Q.-H., Ao W.-L.-J., Tai W.-Q. (2012). Simultaneous determination of seven flavonoids in aerial parts of *Artemisia frigida* by HPLC. Chin. Herb. Med..

[17] Wang Q.-H., Ao W.-L., Dai N.-Y. (2013). Structural elucidation and HPLC analysis of six flavone glycosides from *Artemisia frigida* Willd. Chem. Res. Chin. Univ..

[18] Wang Q., Wu J., Wu X., Han N., Tai W., Dai N., Wu R., Ao W. (2015). Anti-inflammatory effects and structure elucidation of flavonoid and biflavonoid glycosides from *Artemisia frigida* Willd. Monatsh. Chem..

[19] Wang Q., Jin J., Dai N., Han N., Han J., Bao B. (2016). Anti-inflammatory effects, nuclear magnetic resonance identification, and high-performance liquid chromatography isolation of the total flavonoids from *Artemisia frigida*. J. Food Drug Anal..

[20] Belenovskaya L.M., Markova L.P., Kapranova G.I. (1980). Phenolic compounds of *Artemisia frigida*. Chem. Nat. Comp..

[21] Olennikov D.N., Chirikova N.K., Kashchenko N.I., Nikolaev V.M., Kim S.-W., Vennos C. (2018). Bioactive phenolics of the genus *Artemisia* (Asteraceae): HPLC-DAD-ESI-TQ-MS/MS profile of the Siberian species and their inhibitory potential against α-amylase and α-glucosidase. Front. Pharmacol..

[22] Olennikov D.N., Kashchenko N.I. (2019). New flavonoids and turkesterone-2-*O*-cinnamate from leaves of *Rhaponticum uniflorum*. Chem. Nat. Comp..

[23] Olennikov D.N., Kashchenko N.I. (2013). New isorhamnetin glucosides and other phenolic compounds from *Calendula officinalis*. Chem. Nat. Comp..

[24] Chirikova N.K., Olennikov D.N. (2018). Phenolic compounds from Siberian species *Thymus baicalensis* and *T. sibiricus*. Chem. Nat. Comp..

[25] (2013). Organoleptic Analysis of Herbal Ingredients.

[26] (2011). Quality Control Methods for Herbal Materials.

[27] Bradford M.M. (1976). A rapid and sensitive method for the quantification of microgram quantities of protein utilizing the principle of protein-dye binding. Anal. Biochem..

[28] Dubois M., Gilles K.A., Hamilton J.K., Rebers P.A., Smith F. (1956). Colorimetric method for determination of sugars and related substances. Anal. Chem..

[29] Olennikov D.N., Kashchenko N.I., Chirikova N.K. (2017). Meadowsweet teas as new functional beverages: Comparative analysis of nutrients, phytochemicals and biological effects of four *Filipendula* species. Molecules.

[30] Olennikov D.N., Kashchenko N.I., Chirikova N.K., Gornostai T.G., Selyutina I.Y., Zilfikarov I.N. (2017). Effect of low temperature cultivation on the phytochemical profile and bioactivity of Arctic plants: A case of *Dracocephalum palmatum*. Int. J. Molec. Sci..

[31] Chirikova N.K., Olennikov D.N., Tankhaeva L.M. (2010). Quantitative determination of flavonoid content in the aerial parts of Baikal skullcap (*Scutellaria baicalensis* Georgi). Russ. J. Bioorg. Chem..

[32] Olennikov D.N., Tankhaeva L.M. (2010). Quantitative determination of phenolic compounds in *Mentha piperita* leaves. Chem. Nat. Comp..

[33] Olennikov D.N., Fedorov I.A., Kashchenko N.I., Chirikova N.K., Vennos C. (2019). Khellactone derivatives and other phenolics of *Phlojodicarpus sibiricus* (Apiaceae): HPLC-DAD-ESI-QQQ-MS/MS and HPLC-UV profile, and antiobesity potential of dihydrosamidin. Molecules.

[34] Olennikov D.N., Kruglova M.Y. (2013). A new quercetin glycoside and other phenolic compounds from the genus *Filipendula*. Chem. Nat. Comp..

[35] Olennikov D.N., Kashchenko N.I., Chirikova N.K., Tankhaeva L.M. (2015). Iridoids and flavonoids of four Siberian gentians: Chemical profile and gastric stimulatory effect. Molecules.

[36] Olennikov D.N., Tankhaeva L.M., Samuelsen A.B. (2006). Quantitative analysis of polysaccharides from *Plantago major* using the Dreywood method. Chem. Nat. Comp..

[37] Fadhil S., Reza M.H., Rouhollah G., Reza V.R.M. (2007). Spectrophotometric determination of total alkaloids in *Peganum harmala* L. using bromocresol green. Res. J. Phytochem..

[38] Olennikov D.N., Chirikova N.K., Okhlopkova Z.M., Zulfugarov I.S. (2013). Chemical composition and antioxidant activity of *Tánara Ótó* (*Dracocephalum palmatum* Stephan), a medicinal plant used by the North-Yakutian nomads. Molecules.

[39] Prior R.L., Hoang H., Gu L., Wu X., Bacchiocca M., Howard L., Hampsch-Woodill M., Huang D., Ou B., Jacob R. (2003). Assays for hydrophilic and lipophilic antioxidant capacity (oxygen radical absorbance capacity (ORAC(FL)) of plasma and other biological and food samples. J. Agric. Food Chem..

[40] Wu X., Gu L., Holden J., Haytowitz D.B., Gebhardt S.E., Beecher G., Prior R.L. (2004). Development of a database for total antioxidant capacity in foods: A preliminary study. J. Food Comp. Anal..

[41] Olennikov D.N., Kashchenko N.I., Chirikova N.K. (2015). In Vitro bioaccessibility, human gut microbiota metabolites and hepatoprotective potential of chebulic ellagitannins: A case of Padma Hepaten^®^ formulation. Nutrients.

[42] Lin L.-Z., Hanley J.M. (2008). Identification of hydroxycinnamoylquinic acids of arnica flowers and burdock roots using a standardized LC-DAD-ESI/MS profiling method. J. Agric. Food Chem..

[43] Lu Y., Sun Y., Foo L.Y., McNabb W.C., Molan A.L. (2000). Phenolic glycosides of forage legume *Onobrychis viciifolia*. Phytochemistry.

[44] Azimova S.S., Vinogradova V.I. (2008). Natural Compounds. Flavonoids, Plant Sources, Structures and Properties.

[45] Li Q., Wang L., Dai P., Zeng X., Qi X., Zhu L., Yan T., Wang Y., Lu L., Hu M. (2015). A combined strategy of mass fragmentation, post-column cobalt complexation and shift in ultraviolet absorption spectra to determine the uridine 5′-diphospho-glucuronosyltransferase metabolism profiling of flavones after oral administration of a flavone mixture in rats. J. Chromatogr. A.

[46] Han J., Ye M., Qiao X., Xu M., Wang B., Guo D. (2008). Characterization of phenolic compounds in the Chinese herbal drug *Artemisia annua* by liquid chromatography coupled to electrospray ionization mass spectrometry. J. Pharm. Biomed. Anal..

[47] Alipieva K.I., Kostadinova E., Evstatieva L.N., Stefova M., Bankova V.S. (2009). An iridoid and a flavonoid from *Sideritis lanata*. Fitoterapia.

[48] Ali B., Imran M., Hussain R., Ahmed Z., Malik A. (2010). Structural determination of abutilins A and B, new flavonoids from *Abutilon pakistanicum*, by 1D and 2D NMR spectroscopy. Magn. Reson. Chem..

[49] Iwashina T., Kamenosono K., Ueno T. (1999). Hispidulin and nepetin 4′-glucosides from *Cirsium oligophyllum*. Phytochemistry.

[50] Yang H.-B., Wang Y.-C., Zhang Z.-T., Chang Y. (2018). Synthesis and crystal structure of pilloin. Turk. J. Chem..

[51] Valant-Vetschera K.M., Wollenweber E. (1995). Flavonoid aglycones from the leaf surfaces of some *Artemisia* spp. (Compositae-Anthemideae). Z. Naturforsch..

[52] Luo S.Q., Yuan L., Wu Y.K., Huang J.G. (2013). Effect of fertilization on phenolic components and antioxidant activities of *Artemisia annua*. China J. Chin. Mater. Med..

[53] Pereira C.G., Barreira L., Bijttebier S., Pieters L., Marques C., Santos T.F., Rodrigues M.J., Varela J., Custódio L. (2018). Health promoting potential of herbal teas and tinctures from *Artemisia campestris* subsp. *maritima*: From traditional remedies to prospective products. Sci. Rep..

[54] Melikoglu G., Cubukcu B., Ozhatay N. (2003). Flavonoids of *Artemisia caucasica*. J. Fac. Pharm. Istanbul Univ..

[55] Cubukcu B., Melikoglu G. (1995). Flavonoids of *Artemisia austriaca*. Planta Med..

[56] Belenovskaya L.M., Markova L.P., Kapranova G.I. (1982). Phenolic compounds of *Artemisia xerophytica*. Chem. Nat. Comp..

[57] Kikhanova Z.S., Iskakova Z.B., Dzhalmakhanbetova R.I., Seilkhanov T.M., Ross S.A., Suleimen E.M. (2013). Constituents of *Artemisia austriaca* and their biological activity. Chem. Nat. Comp..

[58] Al-Hazimi H.M.G., Basha R.M.Y. (1991). Phenolic compounds from various *Artemisia* species. J. Chem. Soc. Pak..

[59] Rivero R.M., Ruiz J.M., García P.C., López-Lefebre L.R., Sánchez E., Romero L. (2001). Resistance to cold and heat stress: Accumulation of phenolic compounds in tomato and watermelon plants. Plant Sci..

[60] Olennikov D.N., Kashchenko N.I., Chirikova N.K. (2015). Spinacetin, a new caffeoylglucoside, and other phenolic compounds from *Gnaphalium uliginosum*. Chem. Nat. Comp..

[61] Olennikov D.N., Chirikova N.K. (2016). Caffeoylglucaric acids and other phenylpropanoids of the Siberian *Leonurus* species. Chem. Nat. Comp..

[62] Olennikov D.N., Kashchenko N.I., Chirikova N.K., Koryakina L.P., Vladimirov L.N. (2015). Bitter gentian teas: Nutritional and phytochemical profiles, polysaccharide characterisation and bioactivity. Molecules.

[63] Dai J., Mumper R.J. (2010). Plant phenolics: Extraction, analysis and their antioxidant and anticancer properties. Molecules.

[64] Yu F., Qian H., Zhang J., Sun J., Ma Z. (2018). Simultaneous quantification of eight organic acid components in *Artemisia capillaris* Thunb (Yinchen) extract using high-performance liquid chromatography coupled with diode array detection and high-resolution mass spectrometry. J. Food Drug Anal..

[65] Farah A., Lima J.D.P. (2019). Consumption of chlorogenic acids through coffee and health implications. Beverages.

[66] Li X., Li K., Xie H., Xie Y., Li Y., Zhao X., Jiang X., Chen D. (2018). Antioxidant and cytoprotective effects of the di-*O*-caffeoylquinic acid family: The mechanism, structure—Activity relationship, and conformational effect. Molecules.

[67] Alminger M., Aura A.M., Bohn T., Dufour C., El S.N., Gomes A., Karakaya S., Martínez-Cuesta M.C., McDougall G.J., Requena T. (2014). In Vitro models for studying secondary plant metabolite digestion and bioaccessibility. Compr. Rev. Food Sci. Food Saf..

[68] Bohn T. (2014). Dietary factors affecting polyphenol bioavailability. Nutr Rev..

[69] Pandey A.K., Singh P. (2017). The genus *Artemisia*: A 2012–2017 literature review on chemical composition, antimicrobial, insecticidal and antioxidant activities of essential oils. Medicines.

[70] Lima K., Silva O., Figueira M.E., Pires C., Cruz D., Gomes S., Maurício E.M., Duarte M.P. (2019). Influence of the in vitro gastrointestinal digestion on the antioxidant activity of *Artemisia gorgonum* Webb and *Hyptis pectinata* (L.) Poit. infusions from Cape Verde. Food Res. Int..

