# Supplementary material for: Caffeoylquinic Acids and Flavonoids of Fringed Sagewort (Artemisia frigida Willd.): HPLC-DAD-ESI-QQQ-MS Profile, HPLC-DAD Quantification, in Vitro Digestion Stability, and Antioxidant Capacity
Source: Antioxidants (Basel). 2019 Aug 14;8(8):307. doi: 10.3390/antiox8080307 (PMC6720735; doi:10.3390/antiox8080307)
Supplement: Supplementary file 1 [file antioxidants-08-00307-s001.pdf]

antioxidants

MDPI

Supplementary materials

Caffeoylquinic Acids and Flavonoids of Fringed Sagewort (*Artemisia frigida* Willd.): HPLC-DAD-ESI-QQQ-MS Profile, HPLC-DAD Quantification, *in vitro* Digestion Stability, and Antioxidant Capacity

Daniil N. Olennikov [1,*], Nina I. Kashchenko [1], Nadezhda K. Chirikova [2], Aina G. Vasil'eva [2], Aydan I. Gadimli [3], Javanshir I. Isaev [3], and Cecile Vennos [4]

[1] Laboratory of Medical and Biological Research, Institute of General and Experimental Biology, Siberian Division, Russian Academy of Science, 6 Sakh'yanovoy Street, Ulan-Ude 670047, Russia; ninkk@mail.ru
[2] Department of Biochemistry and Biotechnology, North-Eastern Federal University, 58 Belinsky Street, Yakutsk 677027, Russia; hofnung@mail.ru (N.K.C.), ainagvas@mail.ru (A.G.V.)
[3] Department of Pharmacognosy, Azerbaijan Medical University, Anvar Gasimzade Street 14, Baku AZ1022, Azerbaijan; aydangadimli25@gmail.com (A.I.G.), isayev.cavanshir@amu.edu.az (J.I.I.)
[4] Regulatory and Medical Scientific Affairs, Padma AG, 1 Underfeldstrasse, Hinwil CH-8340, Switzerland; c.vennos@padma.ch
* Correspondence: olennikovdn@mail.ru; Tel.: +7-9021-600-627 (D.N.O.)

Received: date; Accepted: date; Published: date

Abstract: Fringed sagewort (*Artemisia frigida* Willd., Compositae family) is a well-known medicinal plant in Asian medical systems. Fifty-nine hydroxycinnamates and flavonoids have been found in *A. frigida* herbs of Siberian origin by high-performance liquid chromatography with diode array and electrospray triple quadrupole mass detection (HPLC-DAD-ESI-QQQ-MS). Their structures were determined after mass fragmentation analysis as caffeoylquinic acids, flavone *O*-/*C*-glycosides, flavones and flavonol aglycones. Most of the discovered components were described in *A. frigida* for the first time. It was shown that flavonoids with different types of substitution have chemotaxonomic significance for species of *Artemisia* subsection Frigidae (section Absinthium). After HPLC-DAD quantification of 16 major phenolics in 21 Siberian populations of *A. frigida* and subsequent principal component analysis, we found substantial variation in the selected compounds, suggesting the existence of two geographical groups of *A. frigida*. The antioxidant activity of *A. frigida* herbal tea was determined using 2,2-diphenyl-1-picrylhydrazyl free radical (DPPH•) and hydrophilic/lipophilic oxygen radical absorbance capacity (ORAC) assays and DPPH•-HPLC profiling, revealing it to be high. The effect of digestive media on the phenolic profile and antioxidant capacity of *A. frigida* herbal tea was assessed under simulated gastrointestinal digestion. We found a minor reduction in caffeoylquinic acid content and ORAC values, but remaining levels were satisfactory for antioxidant protection. These results suggest that *A. frigida* and its food derivate herbal tea could be recommended as new plant antioxidants rich in phenolics.

Keywords: *Artemisia frigida*; Compositae (Asteraceae); caffeoylquinic acids; flavonoids; HPLC; mass spectrometry; chemotaxonamy; antioxidant capacity; ORAC.

List of Supplementary Content

Table S1. Ethnopharmacological use of *Artemisia frigida* (fringed sagewort) by the various nomadic people.

Table S2. Phenolic compounds of *A. frigida* (literature data).

Table S3. Regression equations, correlation coefficients (r^2), standard deviation (S_{YX}), limits of detection (LOD), limits of quantification (LOQ) and linear ranges for 16 compounds.

Table S4. Intra- and inter-day precision, repeatability, stability and recovery for 16 compounds.

Table S5. Content of selected caffeoylquinic acids and flavonoids in the 21 samples of *A. frigida* herb.

Table S6. Macronutrients, free sugars, organic acids, amino acids and mineral composition of *A. frigida* herbal tea.

Table S7. Demographic information of participants of tea sensory evaluation.

Table S8. Sensory evaluation data of *A. frigida* herbal tea, green tea, black tea and *Artemisia absinthium* tea.

Table S9. Phytochemical composition of *A. frigida* herbal tea.

Figure S1. Mass spectra of compounds **1–59**, found in *A. frigida*.

Figure S2. Structures of reference standards used in present work.

Figure S3. A series of three successive chromatograms of *A. frigida* extract demonstrating good reproducibility of the method used.

Figure S4. HPLC-DAD chromatograms of *A. frigida* extract of leaves, flowers, stems and roots.

Figure S5. HPLC-DAD chromatogram of the reference mixture of 16 pure compounds and internal standard.

Figure S6. Calibration curves for 16 reference compounds used for quantitative HPLC-DAD assay.

Figure S7. Sensory profiles of *A. frigida* herbal tea, green tea, black tea and *Artemisia absinthium* tea according to 30 participants estimation.

Figure S8. HPLC-DAD chromatogram of *A. frigida* herbal tea.

Table S1. Ethnopharmacological use of *Artemisia frigida* (fringed sagewort) by the various nomadic people.

Description of use	Citation [ref.]
	Buryats, Tuvans, Soyots
Wound healing, kidney and lung diseases	"About white *mkhan pa* [a] called *a krong*: *A krong* grows at the base of gravelly placers. The leaves are white, short, bushy. It height is about span or as an index finger. The flowers are yellow. The smell is sweet. The name is *little mkhan pa with golden head*. It can pacify *skrans*, and used to treat *bras* and wounds, it takes out diseases from the lung and useful for kidneys." [5]
Nosebleeds	"The decoction of barberry wood, cold sagebrush and bear bile best of all stops nosebleeds" (recipe No 113) [6]
Kidney and blood diseases, diabetes, edemas	"The decoction from the buckthorn, ferula, ephedra, cold sagebrush, rhododendron, true cardamom, emblica, phlomis, valerian and juniper berries used for the treatment of kidney diseases, edemas caused by fight of *heat* and *cold*, and blood diseases in general." (recipe No 113) [6]
Kidney diseases, diabetes, diabetes, digestive	"White *a krong* is useful for kidney diseases, and it is a good remedy improves digestion of food juices (also called *bchud len*)." [7]
Hypertension, diabetes, hemorrhage, swollen joints	Verbal information [8].
	Yakuts
Digestive, diabetes, hemorrhage, wound healing	Personal communication (based on the verbal information) [9]
	Mongols
Wound healing, ulcers	Verbal information [8].

[a] "*Mkhan pa*" is the general term for four varieties of *Artemisia* (white, whitish, red, black) described in *Shel Pkhreng*.

Table S2. Phenolic compounds of *A. frigida* (literature data).

Compound	Origin of herb				Content, mg/g	Ref.
	North America	China	Inner Mongolia	Russia		
Flavones						
Apigenin	+				0.0004	[13]
Schaftoside (api-6-*C*-Glc-8-*C*-Ara)				+		[20]
Isoschaftoside (api-6-*C*-Ara-8-*C*-Glc)				+		[20]
Vicenin-2 (api-6,8-di-*C*-Glc)				+		[20]
Hispidulin (dinatin)	+				0.02	[13]
Luteolin	+	+			0.04	[13,14]
Cynaroside (lut-7-*O*-Glc)	+				0.02	[13]
Chrysoeriol	+					[13]
Chrysoeriol-4′-*O*-Glc		+			0.001–12.97	[15,16]
Diosmetin		+			1.11–6.81	[14,16]
Luteolin-3′,4′-dimethyl ether	+					[13]
Luteolin-3′,4′-dimethyl ether-7-*O*-GlcA		+	+		0.0007–1.56	[15,16,19]
5,7,3′,4′,5′-Pentahydroxyflavone-7-*O*-GlcA (friginoside C*)			+			[18]
Tricin	+				0.001	[13]
Tricin-7-*O*-(2″-*O*-GlcA)GlcA-4′-*O*-Glc (friginoside D*)		+			0.38–0.66	[17]
5,7-Dihydroxy-3′,4′,5′-trimethoxyflavone-7-*O*-GlcA (friginoside A)		+	+		0.001–1.09	[15,16,19]
5,7-Dihydroxy-3′,4′,5′-trimethoxyflavone-7-*O*-(2″-*O*-GlcA)GlcA (friginoside B)		+			0.001–3.23	[15,16]
5,7-Dihydroxy-3′,4′,5′-trimethoxyflavone-5-*O*-GlcA-7-*O*-(2″-*O*-GlcA)GlcA (friginoside C*)		+			0.43–0.81	[17]
Nepetin (eupafolin)	+				0.001	[13]
Jaceosidin	+			+	0.009	[13,20]
Desmethylcentaureidin		+	+		0.56–4.40	[15,16,19]
Eupatorin		+	+		0.98–3.83	[15,16,19]
Cirsilineol				+		[20]
Eupatilin	+				0.04	[13]
5,7,3′,4′-Tetrahydroxy-6,5′-dimethoxyflavone	+				0.001	[13]
5,7,3′-Trihydroxy-6,4′,5′-trimethoxyflavone	+					[12]
5,7,4′-Trihydroxy-6,3′,5′-trimethoxyflavone	+					[12]
5,7,3′,4′,5′-Pentahydroxy-6,8-dimethoxyflavone-7-*O*-GlcA-4′-*O*-Glc (friginoside E)		+			0.34–0.49	[17]
Flavonols						
Quercetin		+				[14]
5,3′-Dihydroxy-3,6,7,4′-tetramethoxyflavone		+	+			[14,19]
Quercetagetin-3,6,3′,4′-tetramethyl ether	+				0.001	[13]
Biflavones						
8-*O*-8‴-Biluteolin-7,7‴-*O*-GlcA (friginoside D*)			+			[18]
Coumarins						
Umbelliferone		+				[14]
Herniarin		+				[14]
Esculetin		+				[14]
5,6-Dihydroxy-7-methoxycoumarin		+				[14]
Phenylpropanoids						
Caffeic acid		+				[14]

* This name is also used for compound with another structure. Abbreviation used: api – apigenin, Ara – arabinosyl, Glc – glucosyl, lut – luteolin. The presence of a compound indicated as «+».

Table S3. Regression equations, correlation coefficients (r^2), standard deviation (S_{YX}), limits of detection (LOD), limits of quantification (LOQ) and linear ranges for 16 compounds.

Compound [a]	Regression equation	r^2	S_{YX}	LOD (µg/mL)	LOQ (µg/mL)	Linear range (µg/mL)
4-CQA	$y = 0.046 \cdot x - 0.045$	0.9999	$9.26 \cdot 10^{-3}$	0.68	2.06	2.5–500.0
5-CQA	$y = 0.058 \cdot x - 0.043$	0.9999	$1.53 \cdot 10^{-3}$	0.12	0.36	1.0–500.0
3,4-dCQA	$y = 0.061 \cdot x - 0.011$	0.9999	$2.64 \cdot 10^{-3}$	0.79	2.39	2.5–500.0
3,5-dCQA	$y = 0.063 \cdot x - 0.046$	0.9999	$9.20 \cdot 10^{-3}$	0.66	2.00	2.5–500.0
4,5-dCQA	$y = 0.062 \cdot x - 0.026$	0.9999	$6.61 \cdot 10^{-3}$	0.83	2.51	3.0–500.0
3,4,5-tCQA	$y = 0.072 \cdot x - 0.061$	0.9999	$8.14 \cdot 10^{-3}$	0.84	2.54	3.0–500.0
Vicenin-2	$y = 0.047 \cdot x - 0.035$	0.9999	$4.01 \cdot 10^{-3}$	0.49	1.48	2.0–500.0
Isoorientin	$y = 0.059 \cdot x - 0.018$	0.9999	$6.11 \cdot 10^{-3}$	0.34	1.04	1.5–500.0
Cynaroside	$y = 0.092 \cdot x - 0.012$	0.9999	$9.02 \cdot 10^{-3}$	0.32	0.98	1.0–500.0
Apigenin	$y = 0.037 \cdot x - 0.021$	0.9999	$2.35 \cdot 10^{-3}$	0.21	0.63	1.0–500.0
Hispidulin	$y = 0.025 \cdot x - 0.019$	0.9999	$3.14 \cdot 10^{-3}$	0.41	1.25	1.5–500.0
Jaceosidin	$y = 0.037 \cdot x - 0.039$	0.9999	$4.19 \cdot 10^{-3}$	0.37	1.14	1.5–500.0
LDME	$y = 0.094 \cdot x - 0.060$	0.9999	$8.45 \cdot 10^{-3}$	0.30	0.90	1.0–500.0
Eupatorin	$y = 0.054 \cdot x - 0.075$	0.9999	$6.82 \cdot 10^{-3}$	0.42	1.26	1.5–500.0
Acacetin	$y = 0.065 \cdot x - 0.008$	0.9999	$7.59 \cdot 10^{-3}$	0.39	1.17	1.5–500.0
Cirsimaritin	$y = 0.027 \cdot x - 0.034$	0.9999	$7.82 \cdot 10^{-3}$	0.96	2.89	3.0–500.0

[a] Compounds: CQA – caffeoylquinic acid; dCQA – dicaffeoylquinic acid; tCQA – tricaffeoylquinic acid; LDME – luteolin-3′,4′-dimethyl ether.

Table S4. Intra- and inter-day precision, repeatability, stability and recovery for 16 compounds.

Compound [a]	Precision intra-day (RSD%) n=5	Precision inter-day (RSD%) n=4	Repeatability (RSD%) n=7	Stability (RSD%) n=7	Recovery (%) n=5
4-CQA	1.38	1.09	2.11	2.21	97.73
5-CQA	1.44	1.22	2.34	1.63	100.25
3,4-dCQA	1.72	1.70	1.88	1.84	101.40
3,5-dCQA	2.01	2.16	1.94	1.96	99.78
4,5-dCQA	2.2	2.31	1.77	1.57	96.32
3,4,5-tCQA	1.96	1.89	1.12	1.44	101.14
Vicenin-2	1.83	1.90	1.20	2.02	103.33
Isoorientin	1.67	1.76	2.42	2.14	102.17
Cynaroside	1.71	1.34	2.40	2.10	101.21
Apigenin	1.90	1.55	1.53	2.33	98.16
Hispidulin	2.44	1.68	1.18	1.52	99.45
Jaceosidin	2.09	1.72	2.09	1.63	97.89
LDME	2.11	1.62	2.03	2.09	104.01
Eupatorin	1.23	1.26	2.44	2.37	103.12
Acacetin	1.78	1.29	1.65	1.98	104.14
Cirsimaritin	1.65	2.04	1.79	1.97	95.88

[a] Compounds: CQA – caffeoylquinic acid; dCQA – dicaffeoylquinic acid; tCQA – tricaffeoylquinic acid; LDME – luteolin-3′,4′-dimethyl ether.

antioxidants

MDPI

Table S5. Content of selected caffeoylquinic acids and flavonoids in the 21 samples of *A. frigida* herb, mg/g dry plant weight

Compound	Altai Krai		Buryatia Republic					Chita Oblast		Irkutsk Oblast			Krasnoyarsk Krai		Tyva Republic		Yakutia Republic				
	A-01	A-02	A-03	A-04	A-05	A-06	A-07	A-08	A-09	A-10	A-11	A-12	A-13	A-14	A-15	A-16	A-17	A-18	A-19	A-20	A-21
Caffeoylquinic acids																					
4-*O*-Caffeoylquinic acid	0.33	0.26	0.27	0.29	0.35	0.42	0.44	0.51	0.22	0.39	0.11	0.10	0.25	0.20	1.16	1.84	0.20	0.12	0.18	0.10	0.10
5-*O*-Caffeoylquinic acid	16.29	17.22	12.25	11.37	12.39	16.84	16.52	6.19	5.25	12.46	12.03	11.17	15.24	14.67	15.37	15.94	9.27	6.12	6.97	5.06	2.11
3,4-Di-*O*-caffeoylquinic acid	0.94	0.67	0.60	0.52	0.63	0.79	0.82	1.64	1.53	0.60	0.68	1.63	1.11	2.54	0.92	0.52	3.84	3.60	2.15	2.09	1.04
3,5-Di-*O*-caffeoylquinic acid	14.37	12.61	17.15	16.22	19.14	23.10	24.20	17.44	15.27	18.26	21.12	22.57	14.06	16.37	12.63	10.08	23.41	24.55	22.91	21.50	14.25
4,5-Di-*O*-caffeoylquinic acid	1.75	1.70	1.71	0.94	1.95	2.86	2.73	2.28	1.92	2.63	2.54	3.11	1.63	2.11	0.94	0.95	3.52	4.63	4.58	3.11	2.39
3,4,5-Tri-*O*-caffeoylquinic acid	2.18	2.39	3.06	3.11	4.16	5.63	5.92	5.74	5.06	5.11	5.56	6.16	2.01	2.53	0.97	0.87	7.14	9.83	11.68	12.35	12.09
Subtotal caffeoylquinic acids	35.86	34.85	35.04	32.45	38.62	49.64	50.63	33.80	29.25	39.45	42.04	44.74	34.30	38.42	31.99	30.20	47.38	48.85	48.47	44.21	31.98
Flavonoid glycosides																					
Vicenin-2	2.11	1.63	1.26	1.20	1.06	2.25	1.85	1.09	0.63	1.27	1.09	0.54	2.00	1.52	1.93	2.39	0.37	0.21	0.20	0.15	0.10
Isoorientin	1.92	1.54	2.34	2.14	3.14	1.82	1.82	2.33	2.16	3.54	2.11	2.08	1.63	1.40	2.16	2.92	2.11	2.14	1.75	1.09	0.70
Cynaroside	0.37	0.32	0.21	0.10	0.27	0.25	0.24	0.55	0.29	0.36	0.32	0.35	0.35	0.26	0.54	0.69	0.21	0.27	0.14	0.10	0.12
Subtotal flavonoid glycosides	4.40	3.49	3.81	3.44	4.47	4.32	3.91	3.97	3.08	5.17	3.52	2.97	3.98	3.18	4.63	6.00	2.69	2.63	2.09	1.34	0.92
Flavonoid aglycones																					
Apigenin	0.10	0.12	0.25	0.11	0.31	0.25	0.20	0.05	0.09	0.30	0.11	0.10	0.12	0.10	0.29	0.34	0.10	0.12	0.10	0.10	0.14
Hispidulin	0.11	0.10	0.28	0.14	0.30	0.32	0.40	0.58	0.63	0.42	0.54	0.82	0.10	0.12	0.32	0.31	0.99	1.16	1.22	1.18	0.94
Jaceosidine	0.84	0.86	1.54	1.27	1.87	2.84	3.52	2.59	2.67	1.67	2.63	3.64	0.85	1.34	0.74	0.51	3.29	5.15	4.74	6.69	6.67
Luteolin-3′,4′-dimethyl ether	0.24	0.11	0.57	0.50	0.63	1.12	1.06	0.26	0.14	0.79	1.35	1.84	0.64	0.72	0.14	0.10	2.04	2.63	3.11	3.52	4.18
Eupatorin	0.33	0.35	0.87	0.56	0.74	0.84	0.80	0.85	0.73	0.84	1.12	1.52	0.35	0.47	0.35	0.16	1.62	1.14	2.84	2.80	3.18
Acacetin	0.11	0.10	0.25	0.34	0.20	0.36	0.54	0.10	0.11	0.25	0.42	0.49	0.10	0.14	0.10	0.11	0.30	0.35	0.46	0.54	0.50
Cirsimaritin	0.71	0.74	2.99	2.36	3.26	4.73	5.13	1.26	1.38	4.34	4.84	7.22	1.06	2.18	0.97	0.34	6.39	7.89	9.18	12.31	17.71
Subtotal flavonoid aglycones	2.44	2.38	6.75	5.28	7.31	10.46	16.65	5.69	5.75	8.61	11.01	15.63	3.22	5.07	2.91	1.87	14.73	18.44	21.65	27.14	33.32
Total flavonoids	6.84	5.87	10.56	8.72	11.78	14.78	20.56	9.66	8.83	13.78	14.53	18.60	7.20	8.25	7.54	7.87	17.42	21.07	23.74	28.48	34.24
Total phenolics	42.70	40.72	45.60	41.17	50.40	64.42	66.19	43.46	38.08	53.23	56.57	63.34	41.50	46.67	39.47	38.07	64.80	69.91	72.21	72.69	66.22

antioxidants

MDPI

Table S6. Macronutrients, free sugars, organic acids, amino acids and mineral composition of *A. frigida* herbal tea.

Parameter	Sagaan aya tea
Macronutrients	
	mg/100 mL
Carbohydrates	127.63 ± 2.55
Protein	31.12 ± 0.62
Lipids	8.60 ± 0.17
Ash	5.39 ± 0.21
Free sugars	
	mg/100 mL
Glucose	22.64 ± 0.45
Fructose	7.54 ± 0.14
Sucrose	28.25 ± 0.52
Total free sugars	58.43
Organic acids	
	mg/100 mL
Citric acid	8.16 ± 0.15
Malic acid	14.02 ± 0.24
Oxalic acid	0.16 ± 0.00
Succinic acid	traces
Tartaric acid	traces
Total organic acids	22.34
Amino acids	
Essential proteinogenic amino acids	
	mg/100 mL
Histidine	0.21 ± 0.00
Isoleucine	0.26 ± 0.00
Leucine	0.08 ± 0.00
Lysine	0.14 ± 0.00
Metionine	0.51 ± 0.01
Phenylalanine	0.22 ± 0.00
Treonine	0.34 ± 0.00
Tryptophane	0.09 ± 0.00
Valine	0.61 ± 0.01
Subtotal	2.46
Conditionally essential proteinogenic amino acids	
Arginine	0.47 ± 0.01
Cysteine	0.04 ± 0.00
Glutamine	0.07 ± 0.00
Glycine	0.12 ± 0.00
Proline	10.63 ± 0.21
Tyrosine	0.24 ± 0.00
Subtotal	11.57
Non-essential proteinogenic amino acids	
Alanine	0.52 ± 0.01
Aspartic acid	0.27 ± 0.00
Asparagine	2.87 ± 0.05
Glutamic acid	5.14 ± 0.11
Serine	0.53 ± 0.01
Subtotal	9.33
Non-essential non-proteinogenic amino acids	
γ-Aminobutyric acid	0.60 ± 0.01
4-Hydroxyproline	0.11 ± 0.00
Subtotal	0.72
Total amino acids	24.08
Minerals	
	µg/100 mL
Calcium	627.18
Chromium	0.02
Cobalt	0.01
Copper	52.37
Iron	124.23
Magnesium	163.10
Manganese	6.25
Molybdenum	traces
Nickel	traces
Potassium	2359.37
Selenium	0.11
Sodium	68.27
Zinc	1.42

Table S7. Demographic information of participants of tea sensory evaluation.

**Index**	**Percentage (*n*)**
Gender	
male	40 (12)
female	60 (18)
Age	
18–30	63 (19)
30–40	30 (9)
> 40	7 (2)

Table S8. Sensory evaluation data of *A. frigida* herbal tea, green tea, black tea and *Artemisia absinthium* tea.

**Index**	***A. frigida* herbal tea**	**Green tea**	**Black tea**	***Artemisia absinthium* tea**
Color acceptability	7.5 ± 1.2	7.2 ± 1.0	8.6 ± 0.9	7.1 ± 0.4
Flavor acceptability	7.4 ± 1.4	7.6 ± 1.0	9.1 ± 1.0	3.6 ± 0.3
Saltiness	5.3 ± 0.8	5.1 ± 0.7	5.4 ± 0.6	5.1 ± 0.4
Bitterness	6.3 ± 0.9	5.8 ± 0.5	6.1 ± 0.4	2.0 ± 0.0
Sourness	4.6 ± 0.9	5.1 ± 0.6	5.6 ± 0.6	4.8 ± 0.4
Astringency	6.2 ± 1.1	7.3 ± 0.8	8.1 ± 0.8	3.6 ± 0.5
Sweetness	5.1 ± 0.4	5.1 ± 0.7	5.3 ± 0.4	4.9 ± 0.4
Overall preferences	6.9 ± 0.8	7.1 ± 1.0	7.3 ± 0.8	2.1 ± 0.1

Preference scale ranged from 0 (greatest imaginary dislike) to 10 (greatest imaginary like).

Table S9. Phytochemical composition of *A. frigida* herbal tea.

**Parameter**	**Sagaan aya tea, mg/100 mL**
Phenylpropanoids	42.18 ± 0.84
Flavonoids	4.63 ± 0.09
Coumarins	traces
Anthocyanidins	not detected
Tannins	not detected
Iridoids	not detected
Essential oil	traces
Water soluble polysaccharides	24.11 ± 0.48
Alkaloids	not detected

Figure S1. Mass spectra of compounds **1–59**, found in *A. frigida*.

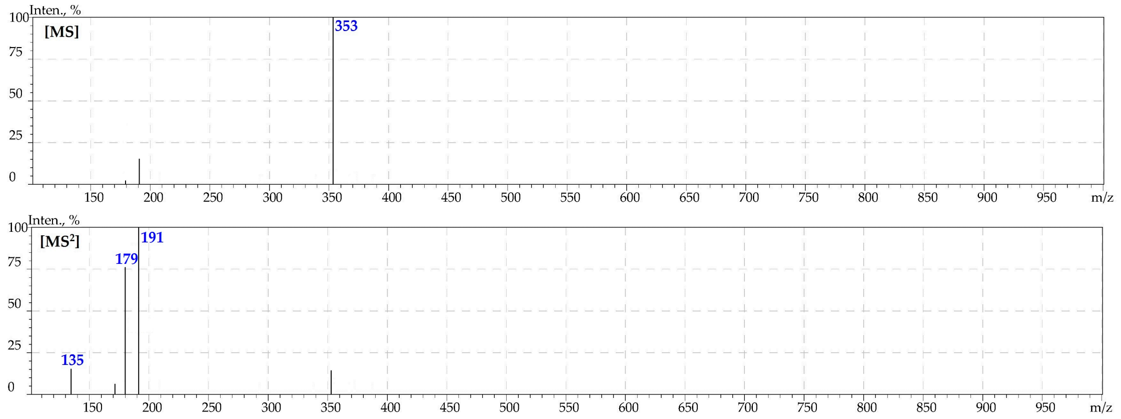

Figure S1-01. MS and MS² spectra of 1-*O*-caffeoylquinic acid (compound **1**).

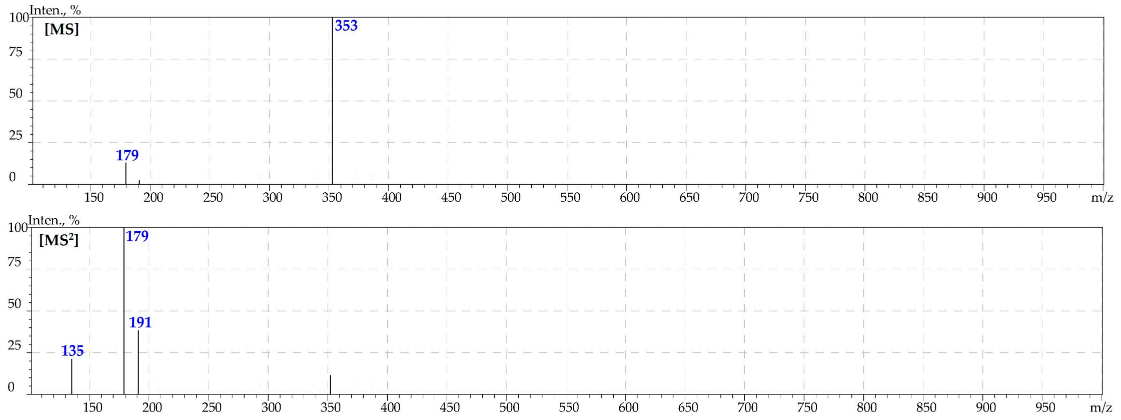

Figure S1-02. MS and MS² spectra of 4-*O*-caffeoylquinic acid (compound **2**).

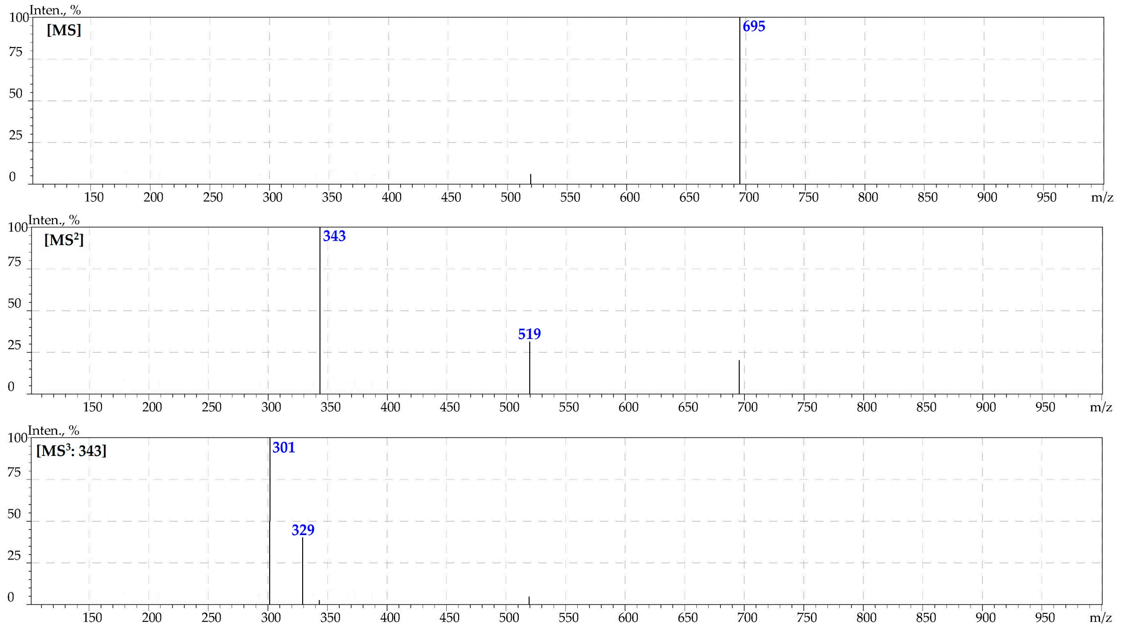

Figure S1-03. MS, MS² and MS³ spectra of friginoside B (compound **3**; tentatively 5,7-dihydroxy-3′,4′,5′-trimethoxyflavone-7-*O*-(2″-*O*-glucuronyl)glucuronide).

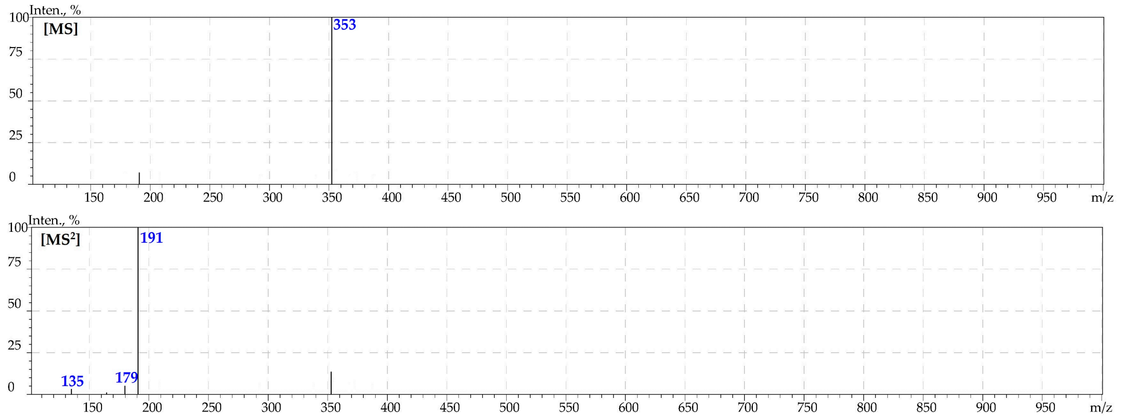

Figure S1-04. MS and MS² spectra of 5-*O*-caffeoylquinic acid (compound **4**).

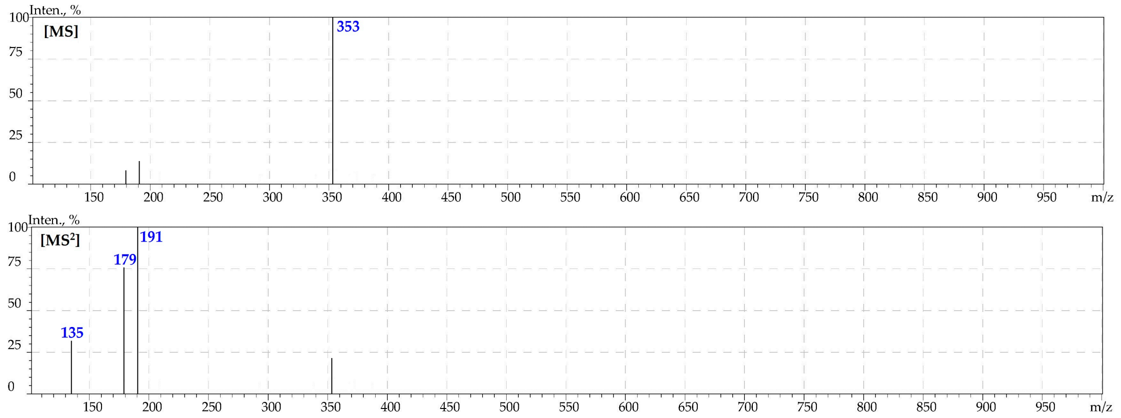

Figure S1-05. MS and MS² spectra of 3-*O*-caffeoylquinic acid (compound **5**).

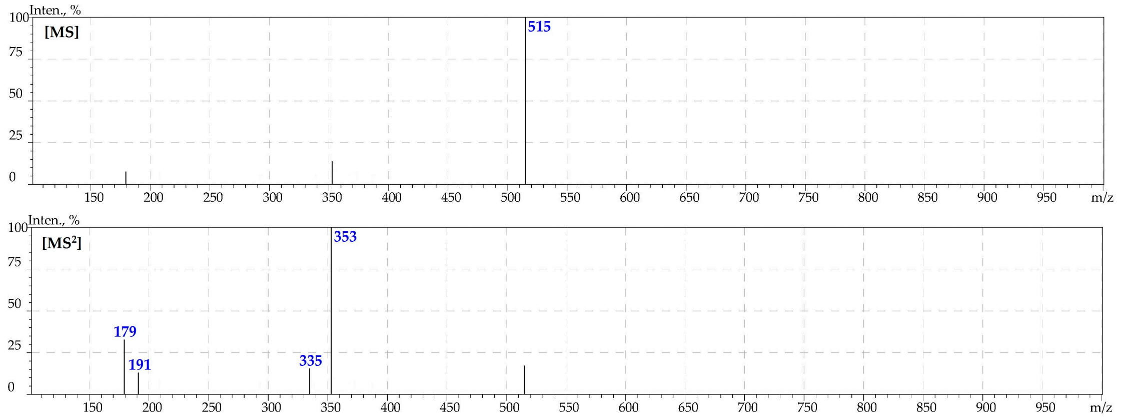

Figure S1-06. MS and MS² spectra of 1,3-di-*O*-caffeoylquinic acid (compound **6**).

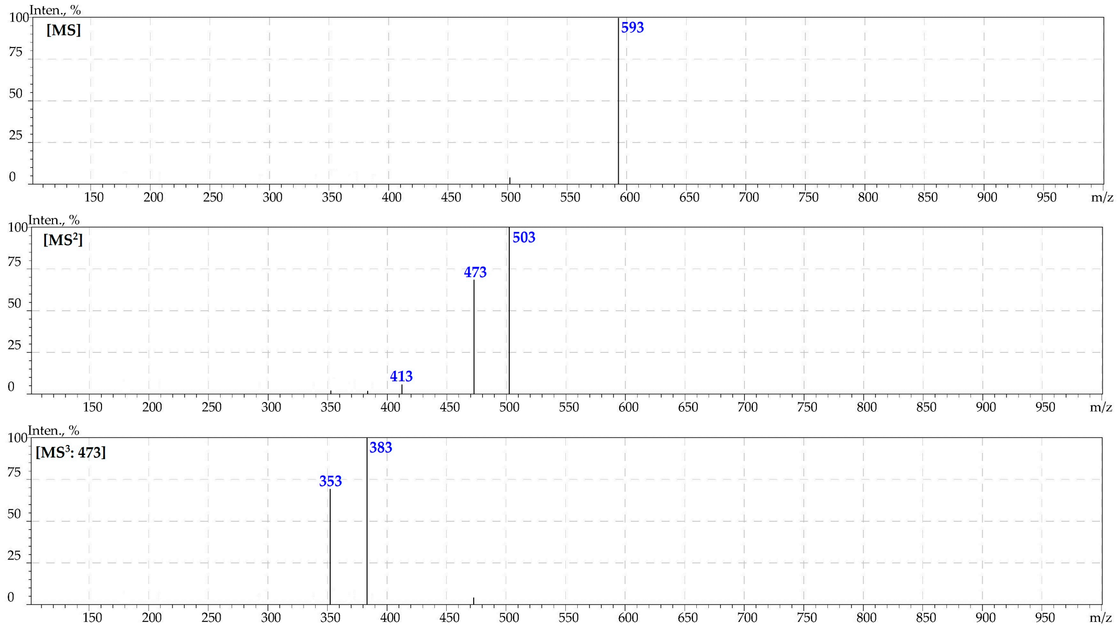

Figure S1-07. MS, MS^2 and MS^3 spectra of vicenin-2 (compound **7**; apigenin-6,8-di-*C*-glucoside).

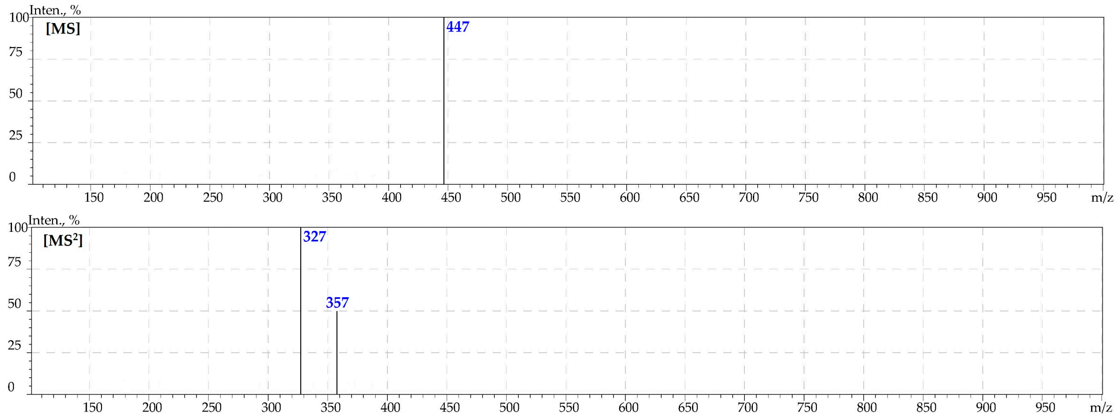

Figure S1-08. MS and MS^2 spectra of isoorientin (compound **8**; luteolin-6-*C*-glucoside).

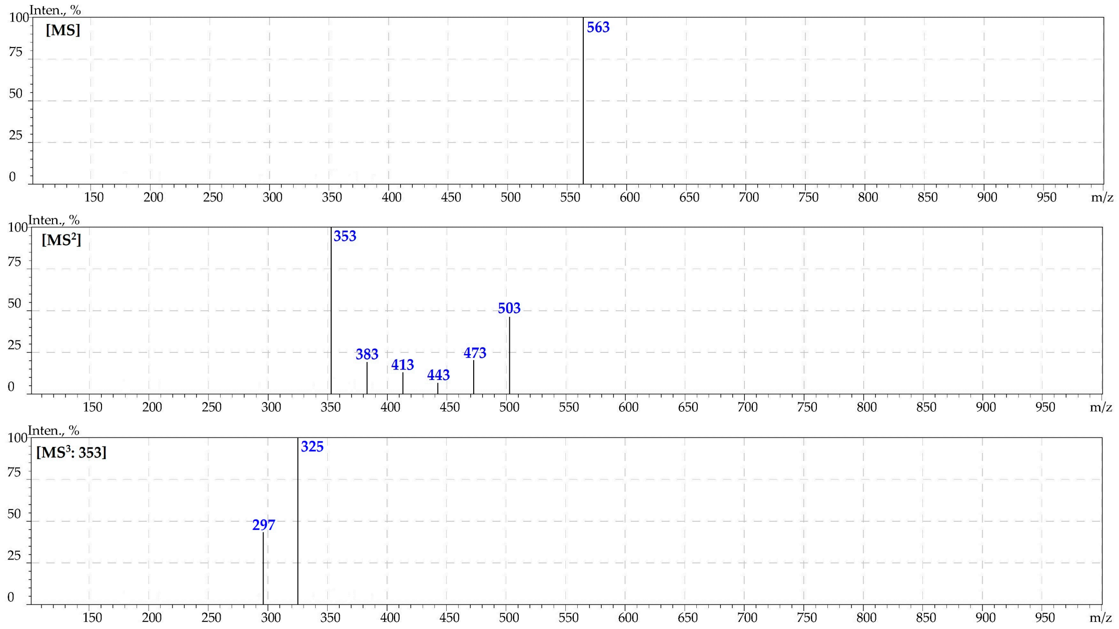

Figure S1-09. MS, MS² and MS³ spectra of isoschaftoside (compound **9**; apigenin-6-*C*-arabinoside-8-*C*-glucoside).

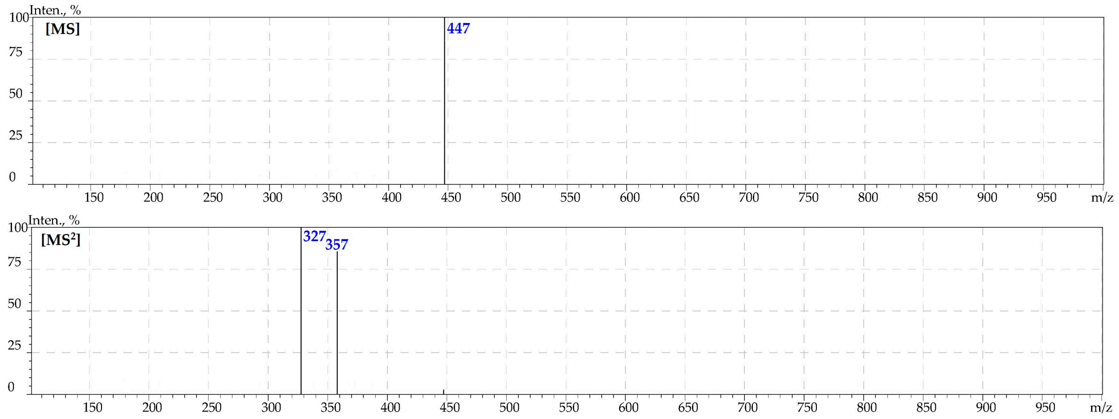

Figure S1-10. MS and MS² spectra of orientin (compound **10**; luteolin-8-*C*-glucoside).

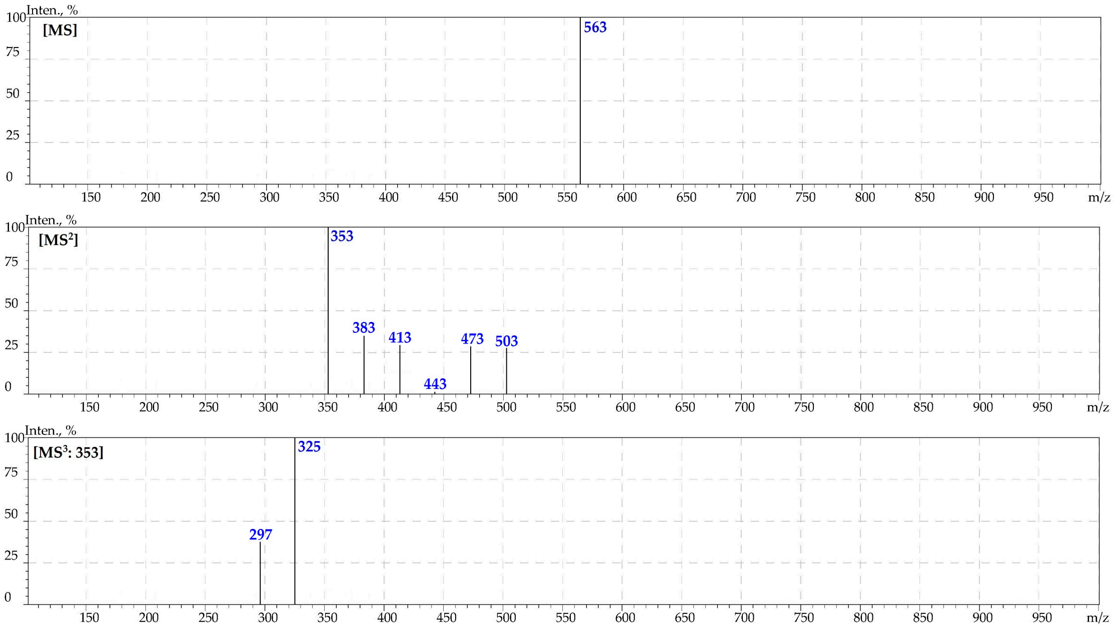

Figure S1-11. MS, MS² and MS³ spectra of schaftoside (compound **11**; apigenin-6-*C*-glucoside-8-*C*-arabinoside).

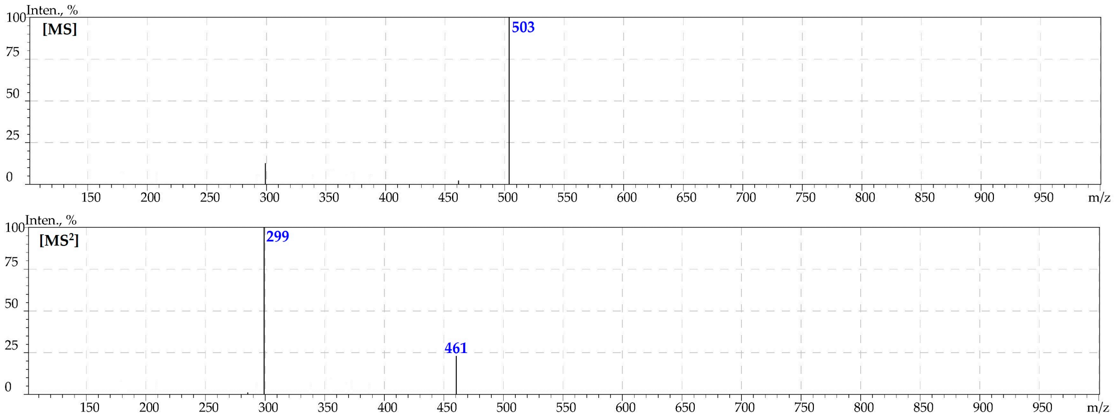

Figure S1-12. MS and MS² spectra of chrysoeriol-*O*-acetyl-hexoside (compound **12**).

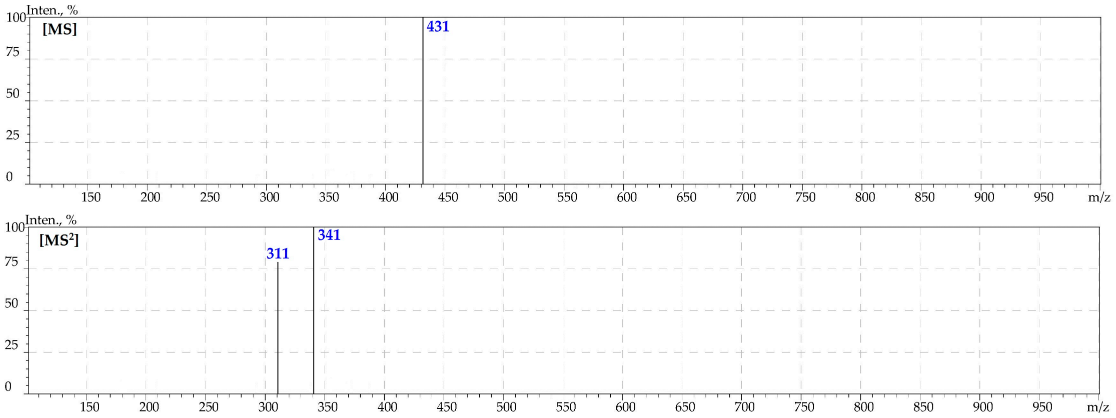

Figure S1-13. MS and MS² spectra of vitexin (compound **13**; apigenin-8-*C*-glucoside).

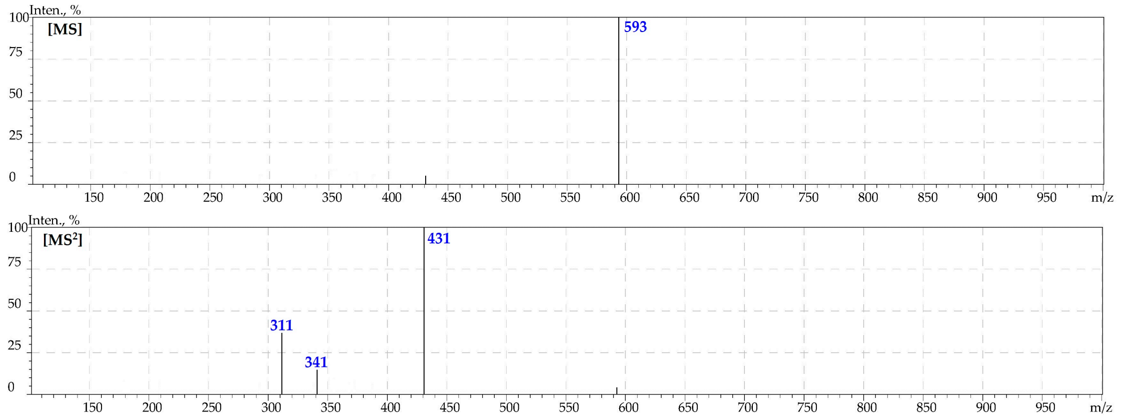

Figure S1-14. MS and MS² spectra of apigenin-*C*-hexoside-*O*-hexoside (compound **14**).

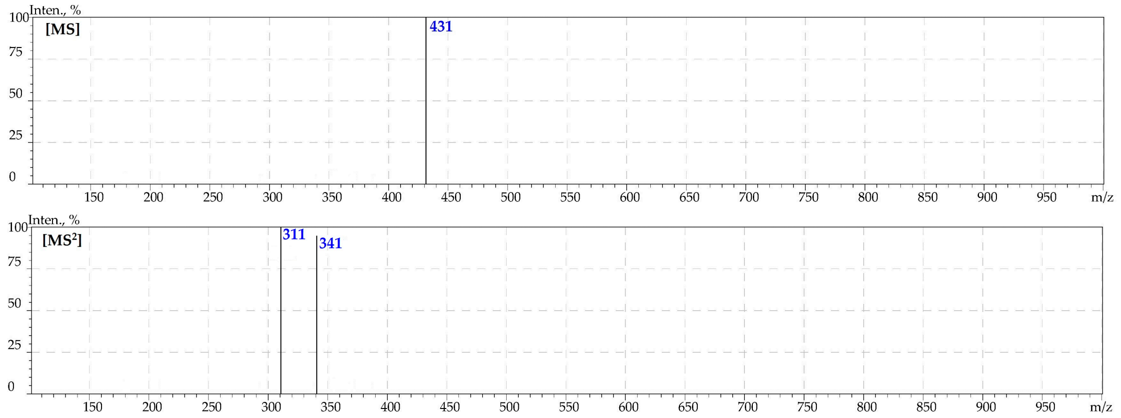

Figure S1-15. MS and MS² spectra of isovitexin (compound **15**; apigenin-6-*C*-glucoside).

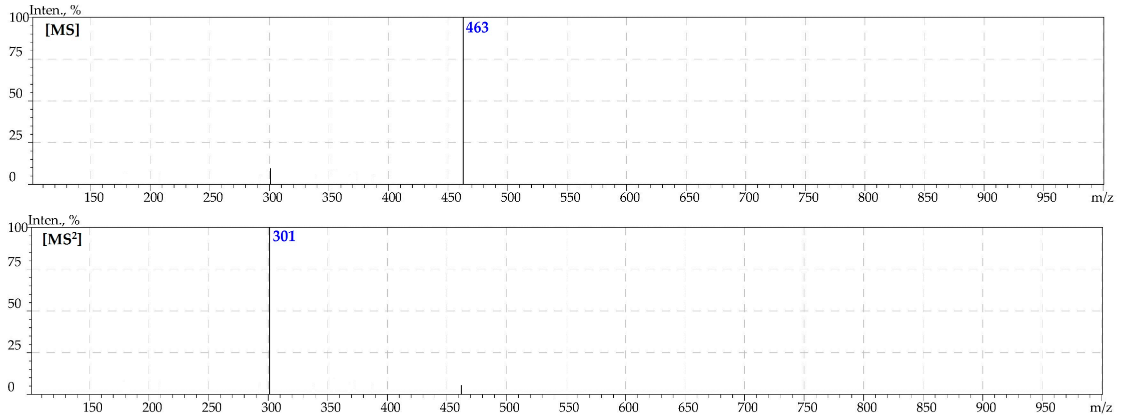

Figure S1-16. MS and MS² spectra of 6-hydroxyluteolin-7-*O*-glucoside (compound **16**).

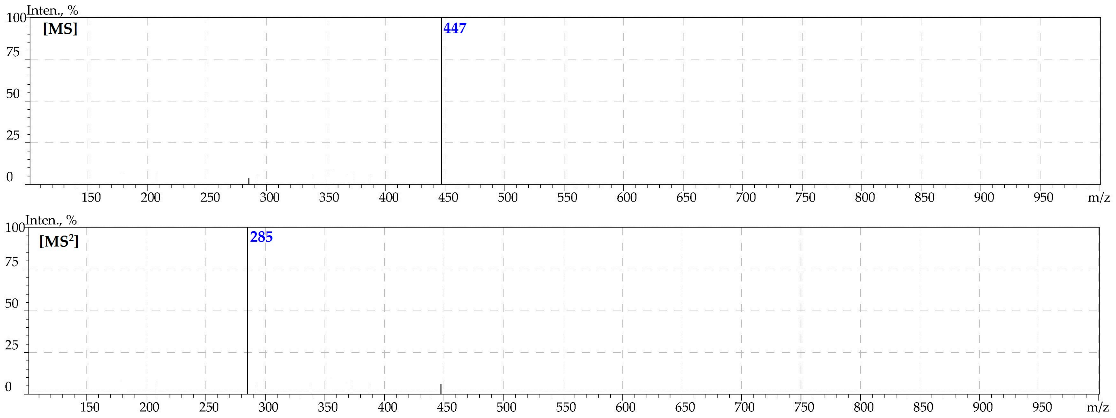

Figure S1-17. MS and MS² spectra of cynaroside (compound **17**; luteolin-7-*O*-glucoside).

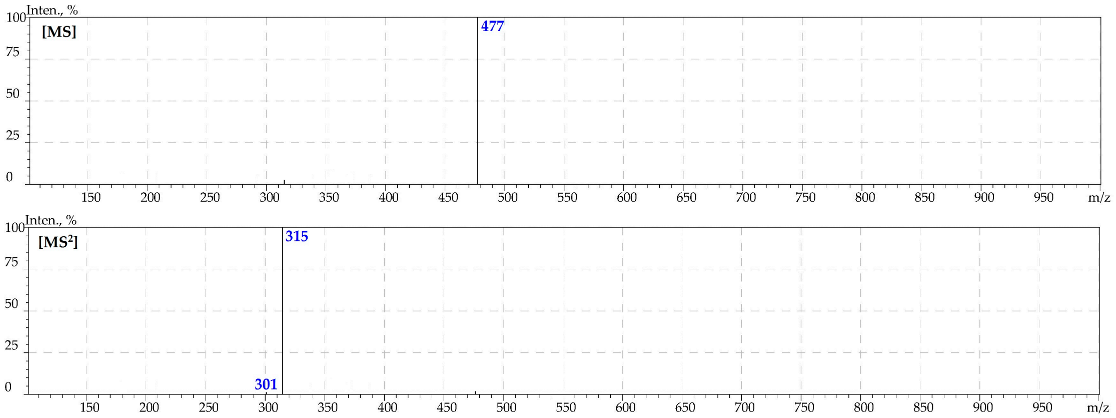

Figure S1-18. MS and MS² spectra of nepitrin (compound **18**; nepetin-7-*O*-glucoside).

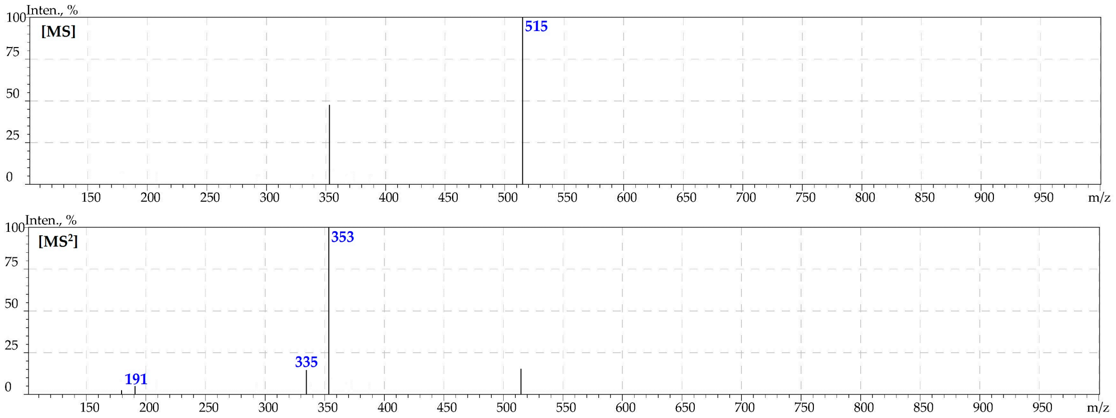

Figure S1-19. MS and MS² spectra of 3,4-di-*O*-caffeoylquinic acid (compound **19**).

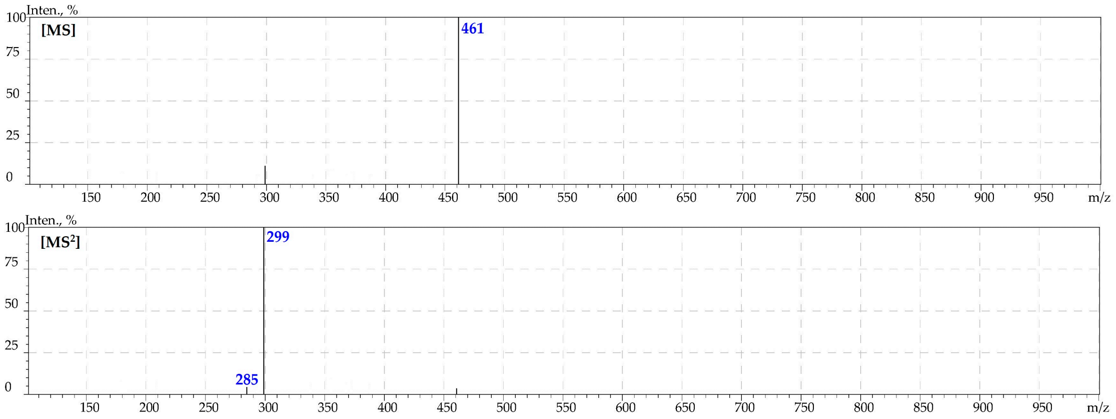

Figure S1-20. MS and MS² spectra of diosmetin-7-*O*-glucoside (compound **20**).

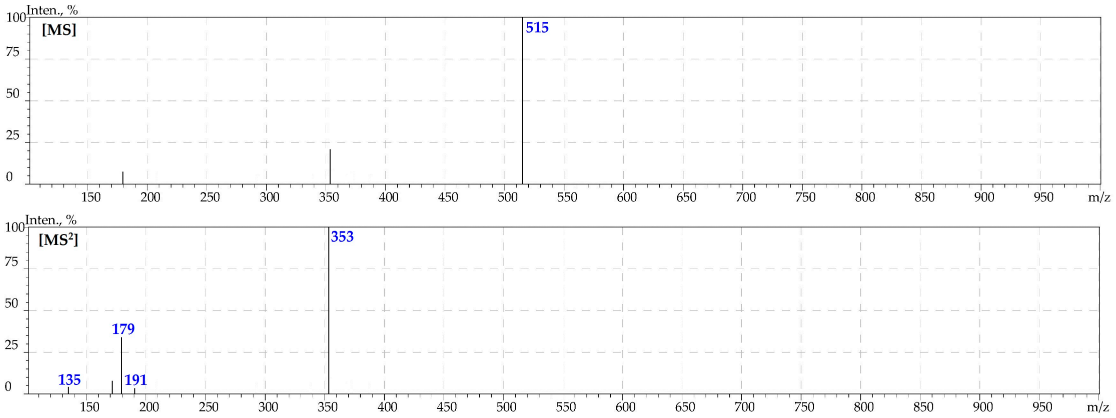

Figure S1-21. MS and MS² spectra of 3,5-di-*O*-caffeoylquinic acid (compound **21**).

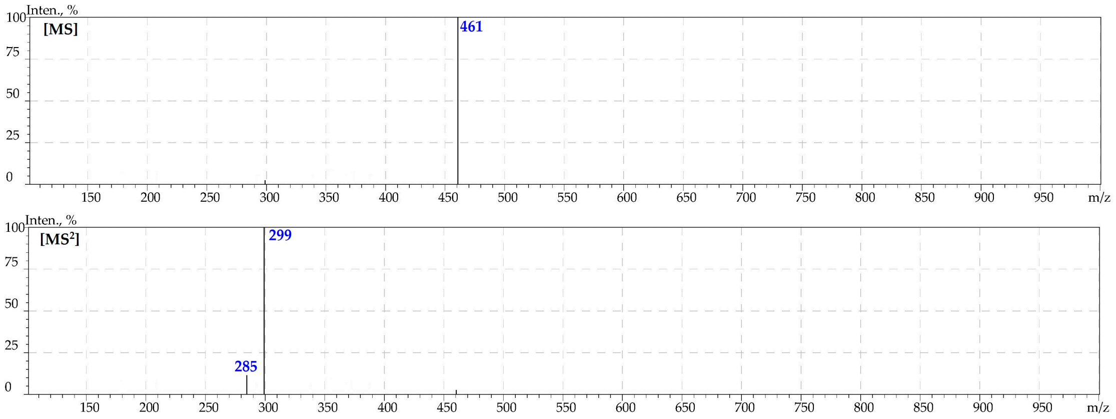

Figure S1-22. MS and MS² spectra of thermopsoside (compound **22**; chrysoeriol-7-*O*-glucoside).

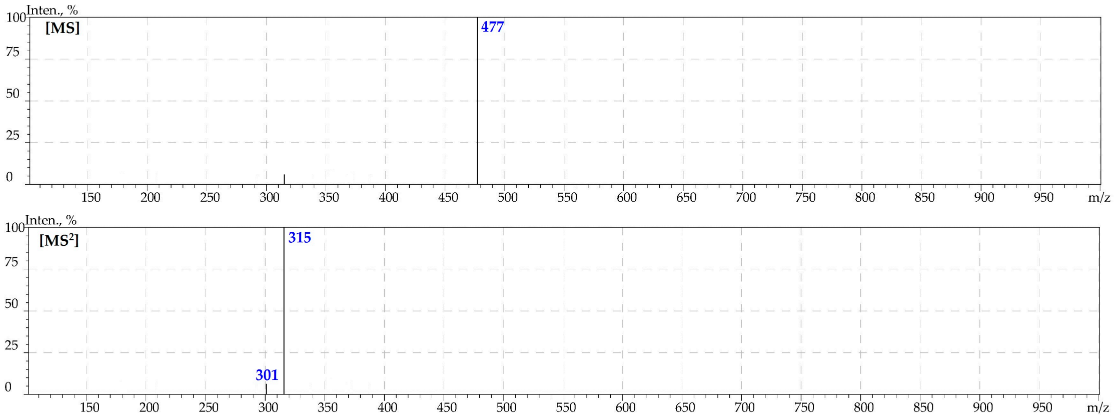

Figure S1-23. MS and MS² spectra of rhaunoside F (compound **23**; nepetin-3′-*O*-glucoside).

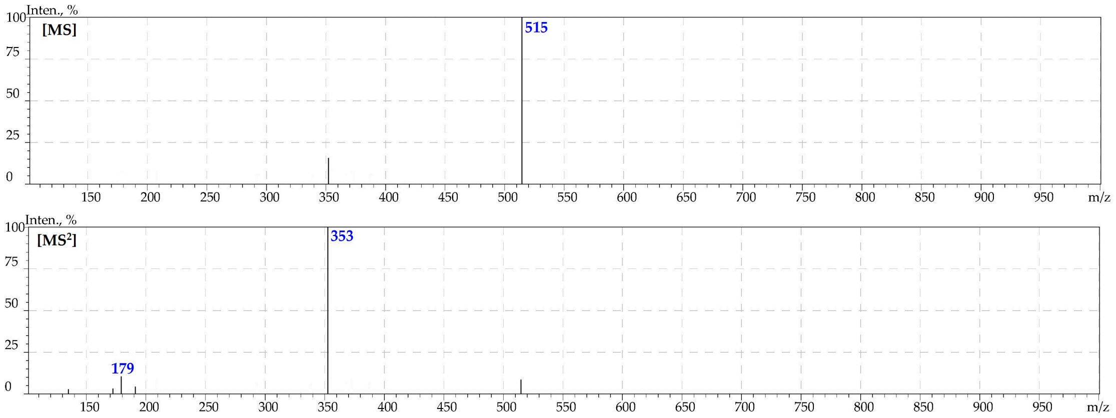

Figure S1-24. MS and MS² spectra of 4,5-di-*O*-caffeoylquinic acid (compound **24**).

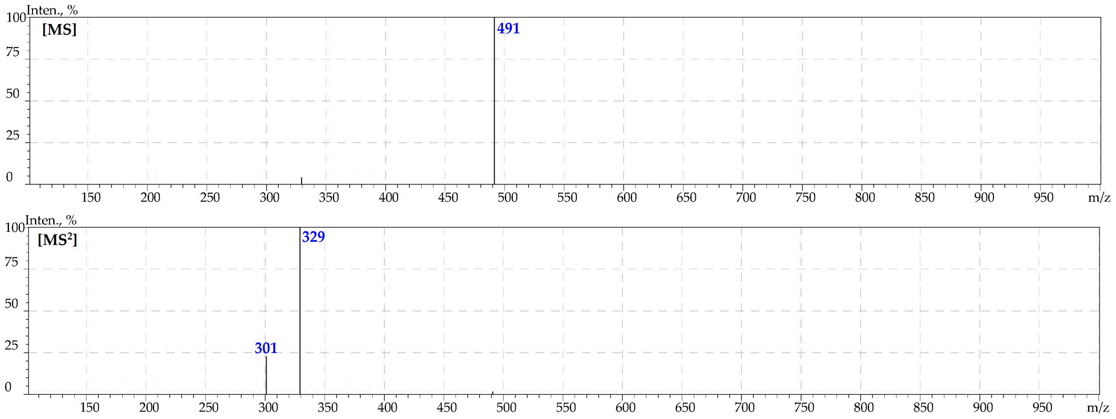

Figure S1-25. MS and MS² spectra of 6-hydroxyluteolin-dimethyl ether-*O*-hexoside (compound **25**).

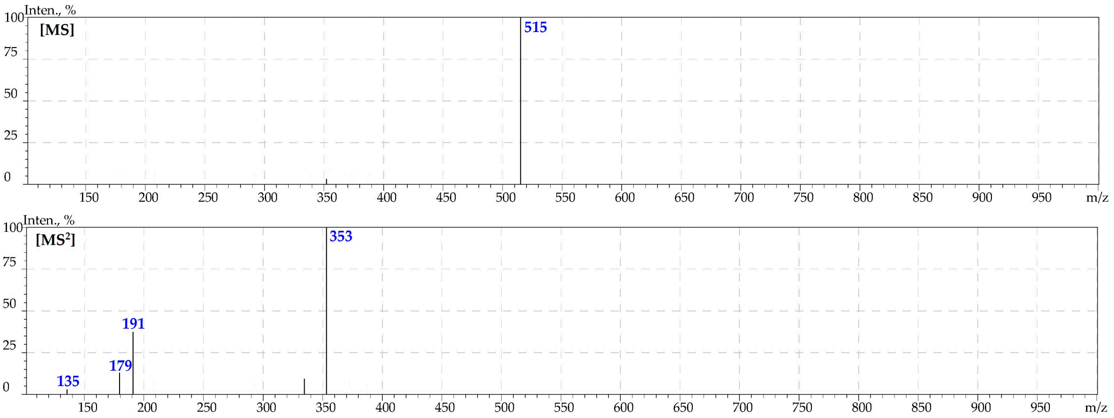

Figure S1-26. MS and MS² spectra of 1,5-di-*O*-caffeoylquinic acid (compound **26**).

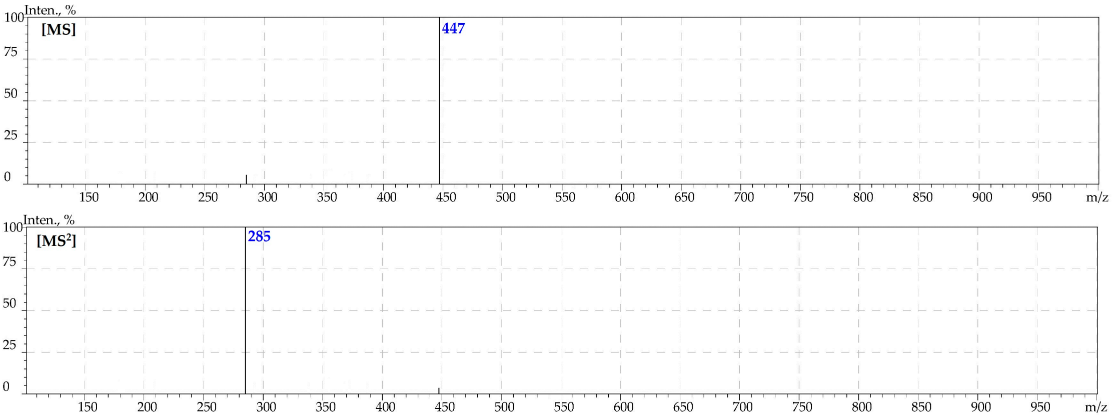

Figure S1-27. MS and MS² spectra of luteolin-4′-*O*-glucoside (compound **27**).

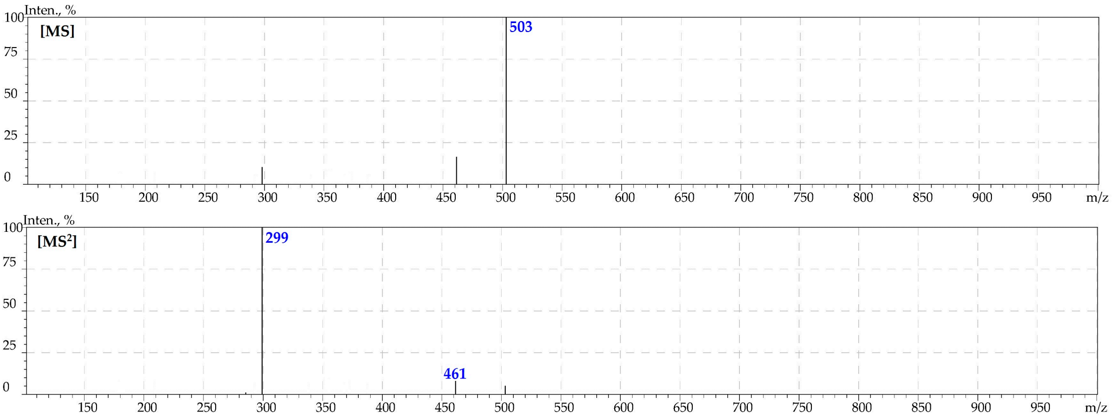

Figure S1-28. MS and MS² spectra of chrysoeriol-*O*-acetyl-hexoside (compound **28**).

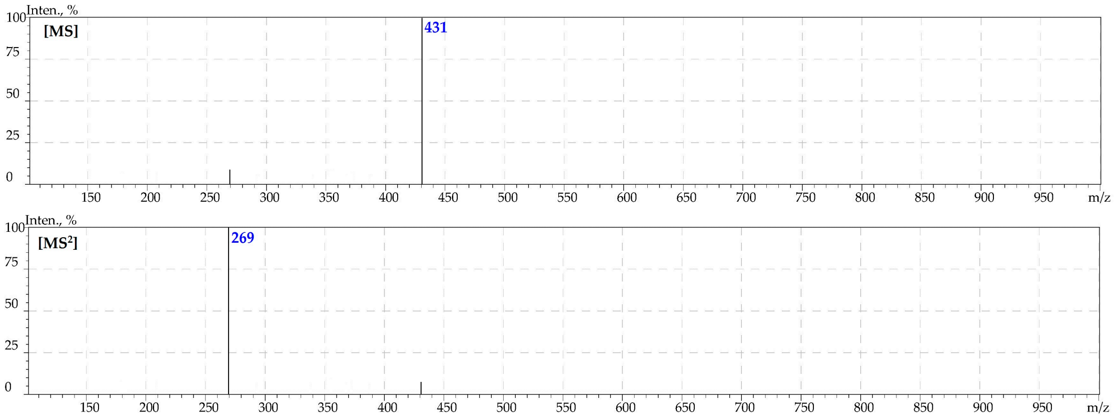

Figure S1-29. MS and MS^2 spectra of cosmosiin (compound **29**; apigenin-7-*O*-glucoside).

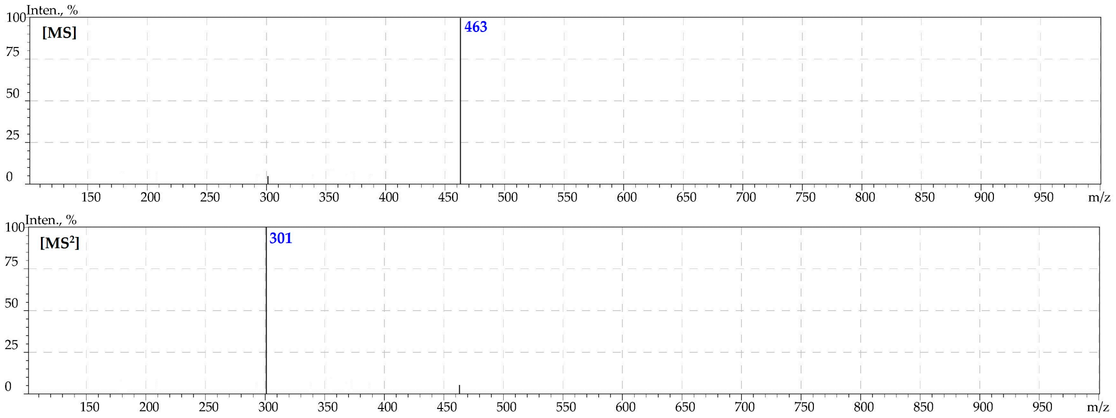

Figure S1-30. MS and MS^2 spectra of 6-hydroxuluteolin-3′-*O*-glucoside (compound **30**; tentative).

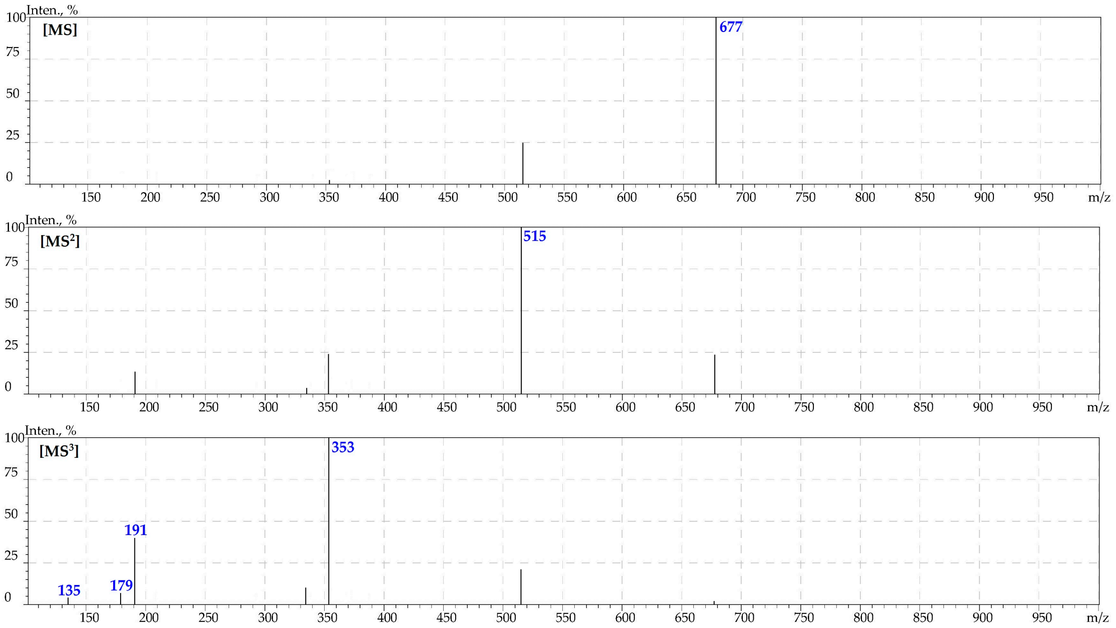

Figure S1-31. MS, MS^2 and MS^3 spectra of 1,3,5-tri-*O*-caffeoylquinic acid (compound **31**).

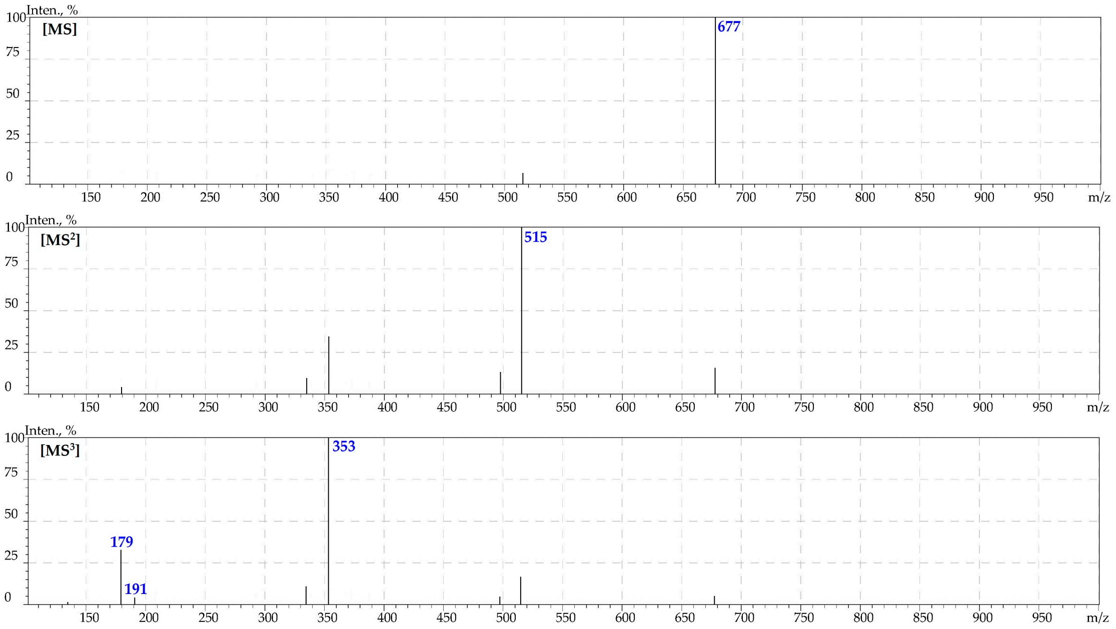

Figure S1-32. MS, MS^2 and MS^3 spectra of 1,4,5-tri-*O*-caffeoylquinic acid (compound **32**).

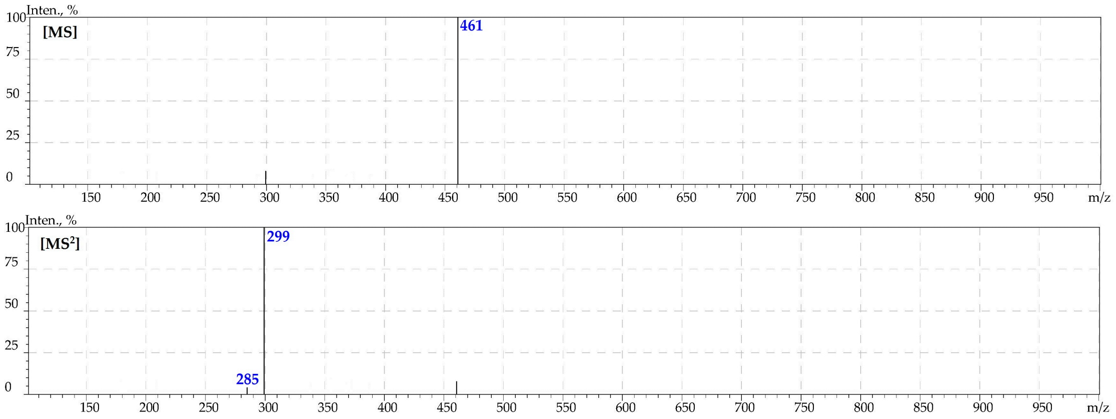

Figure S1-33. MS and MS^2 spectra of chrysoeriol-4′-*O*-glucoside (compound **33**).

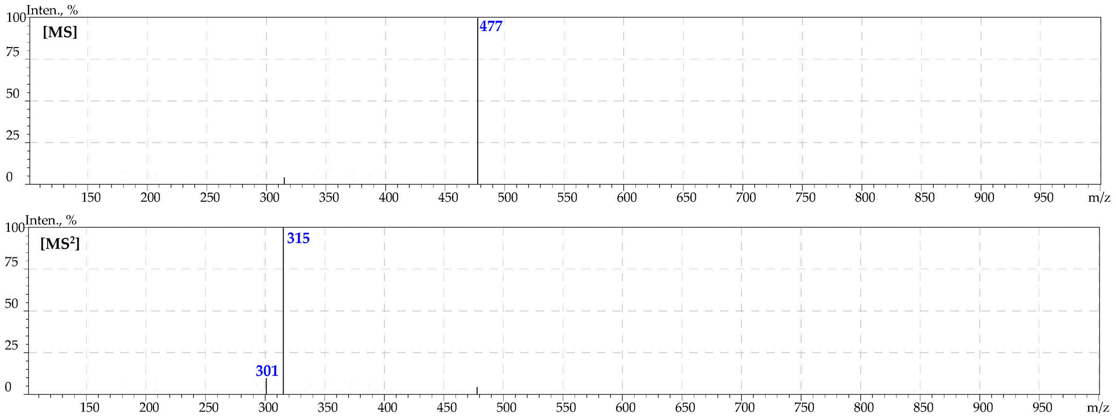

Figure S1-34. MS and MS^2 spectra of nepetin-4′-*O*-glucoside (compound **34**).

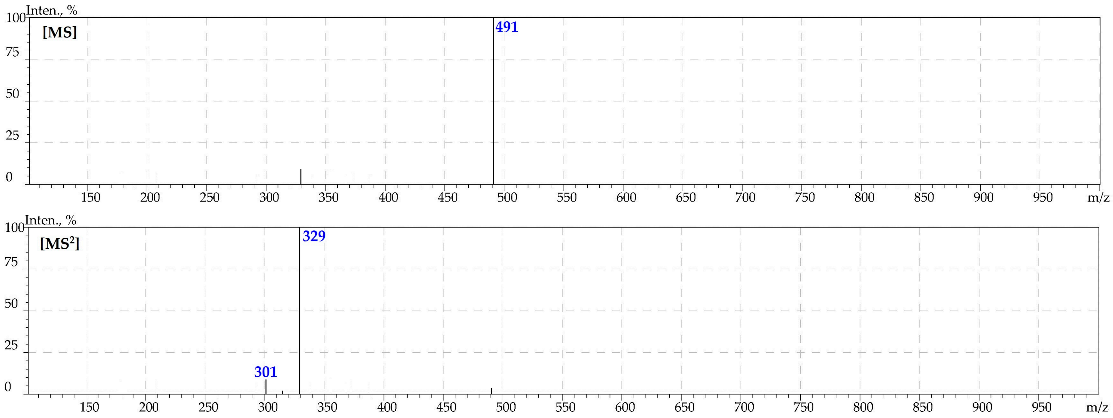

Figure S1-35. MS and MS² spectra of 6-hydroxyluteolin-dimethyl ether-*O*-hexoside (compound **35**).

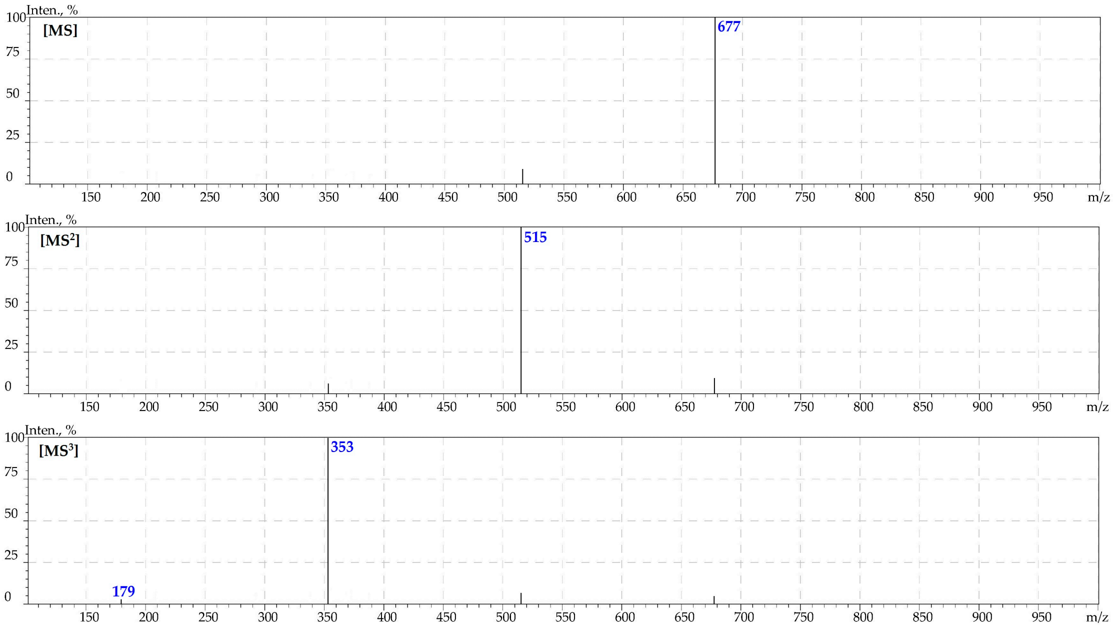

Figure S1-36. MS, MS² and MS³ spectra of 3,4,5-tri-*O*-caffeoylquinic acid (compound **36**).

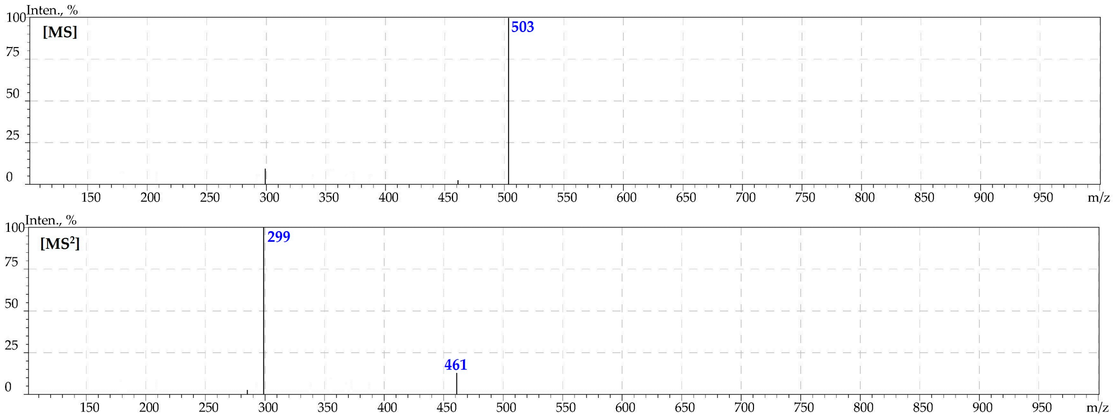

Figure S1-37. MS and MS² spectra of chrysoeriol-*O*-acetyl-hexoside (compound **37**).

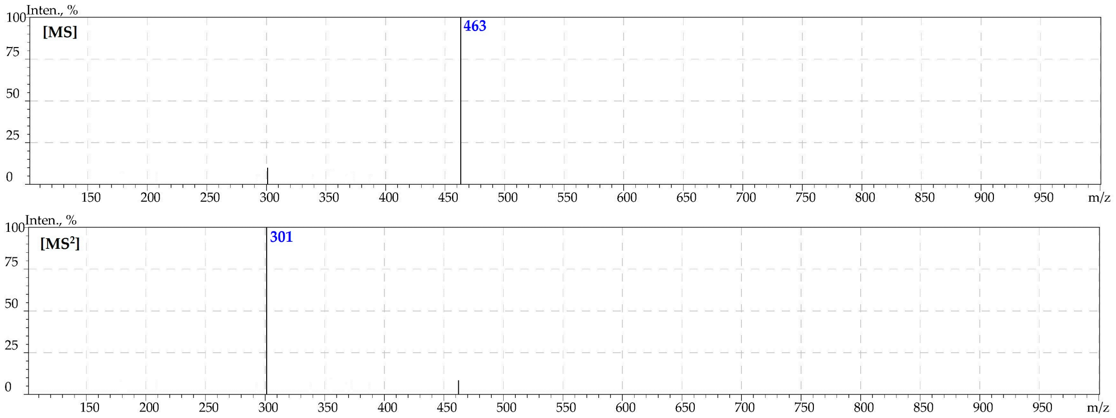

Figure S1-38. MS and MS² spectra of rhaunoside C (compound **38**; 6-hydroxuluteolin-4′-*O*-glucoside).

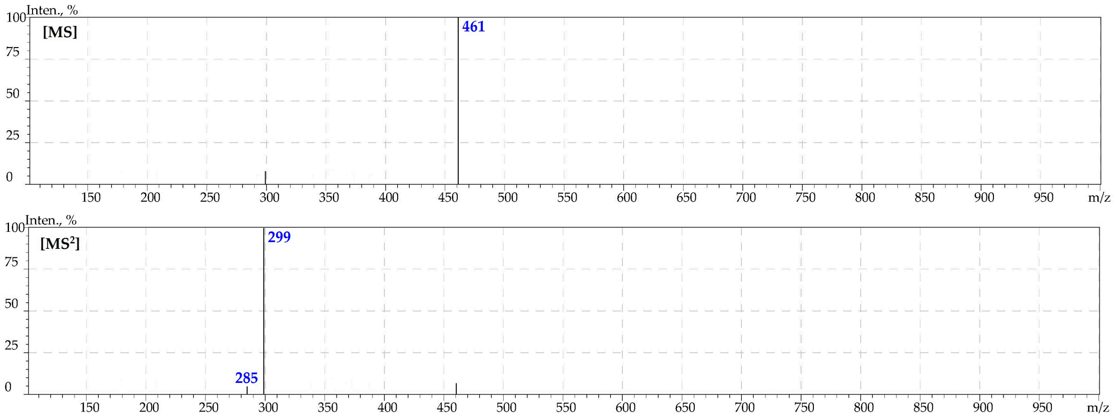

Figure S1-39. MS and MS² spectra of chrysoeriol-5-*O*-glucoside (compound **39**).

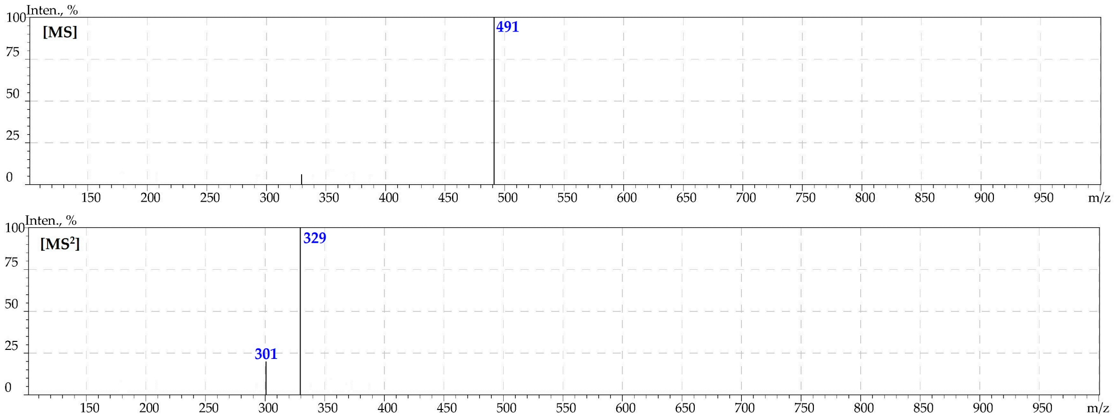

Figure S1-40. MS and MS² spectra of 6-hydroxyluteolin-dimethyl ether-*O*-hexoside (compound **40**).

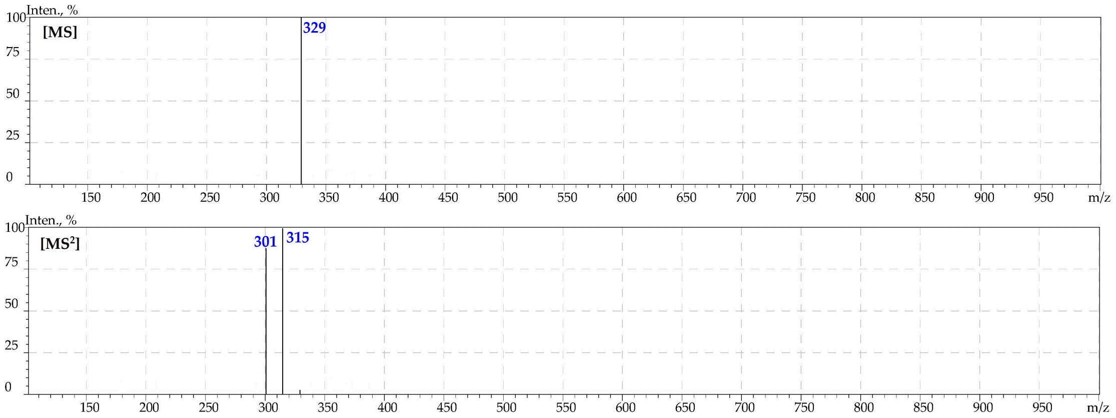

Figure S1-41. MS and MS² spectra of tricin (compound **41**).

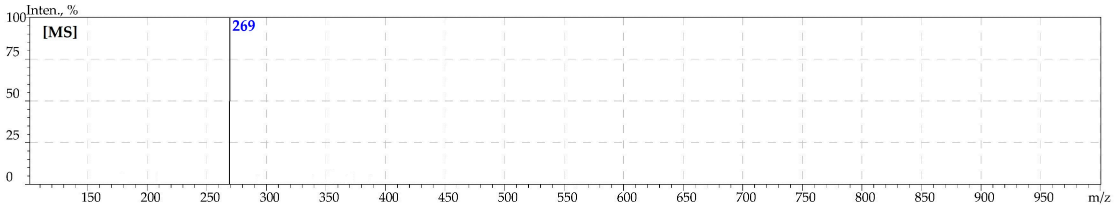

Figure S1-42. MS spectrum of apigenin (compound **42**).

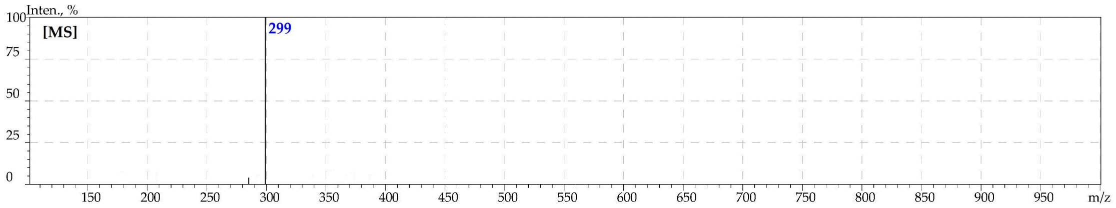

Figure S1-43. MS spectrum of hispidulin (compound **43**).

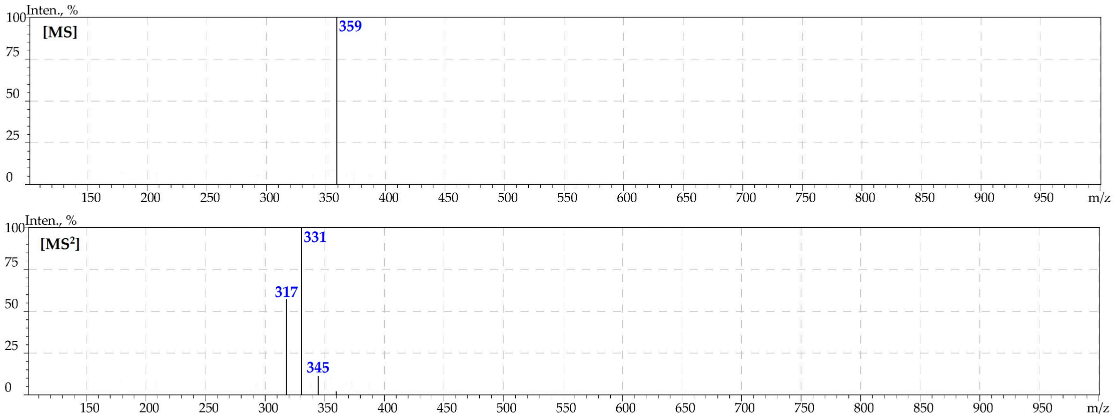

Figure S1-44. MS and MS² spectra of 5,7,3′-trihydroxy-6,4′,5′-trimethoxyflavone (compound **44**; tentative).

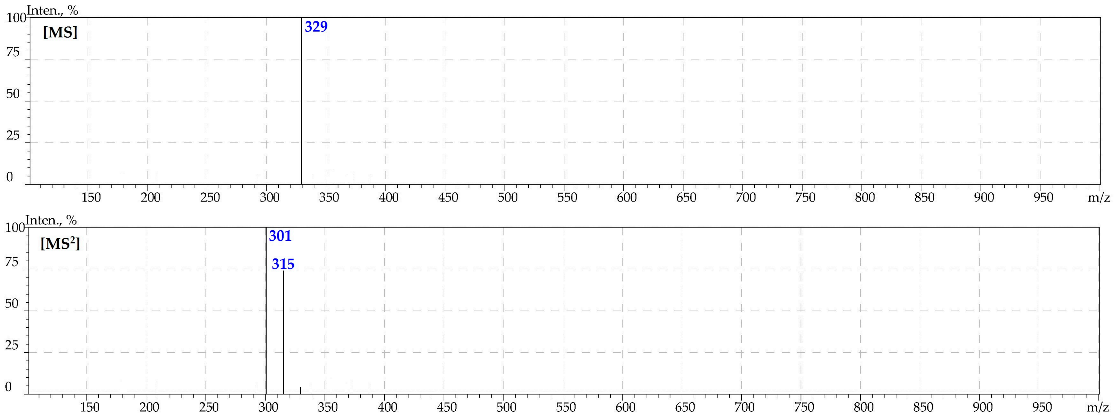

Figure S1-45. MS and MS2 spectra of jaceosidin (compound **45**).

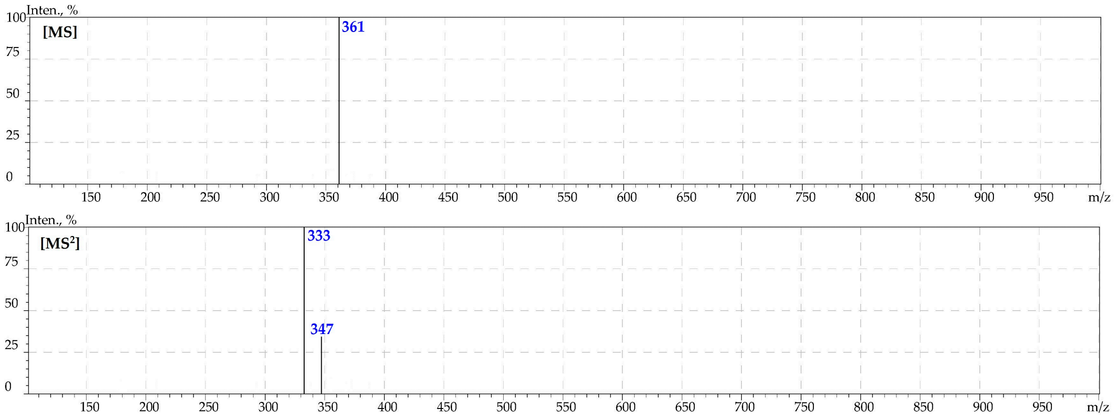

Figure S1-46. MS and MS2 spectra of 5,7,3′,4′,5′-pentahydroxy-6,8-dimethoxyflavone (compound **46**; tentative).

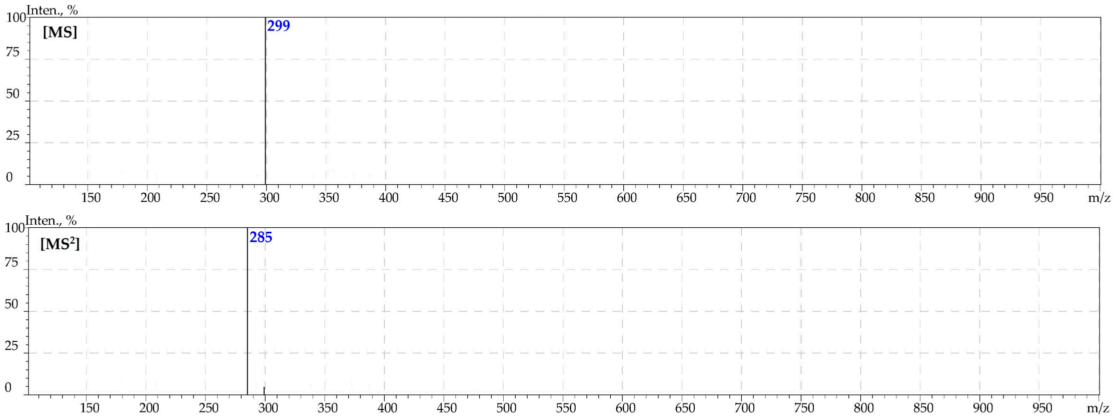

Figure S1-47. MS and MS2 spectra of chrysoeriol (compound **47**).

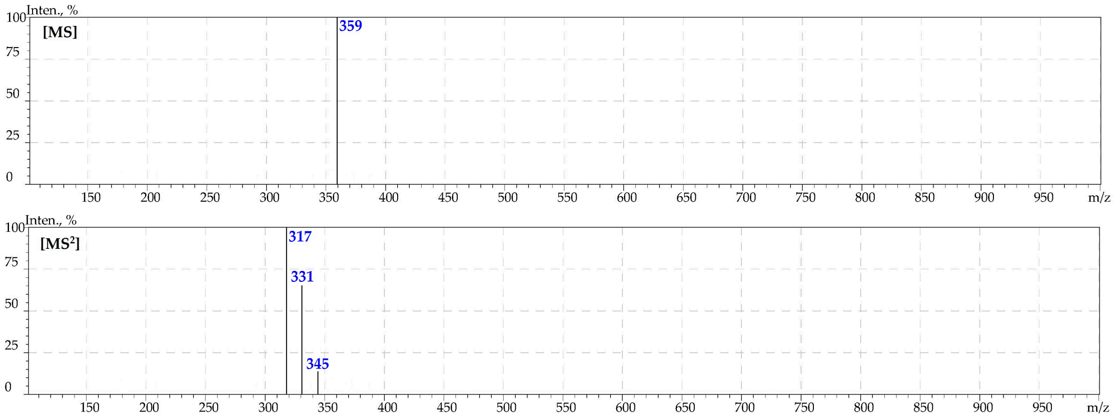

Figure S1-48. MS and MS^2 spectra of 5,7,4′-trihydroxy-6,3′,5′-trimethoxyflavone (compound **48**; tentative).

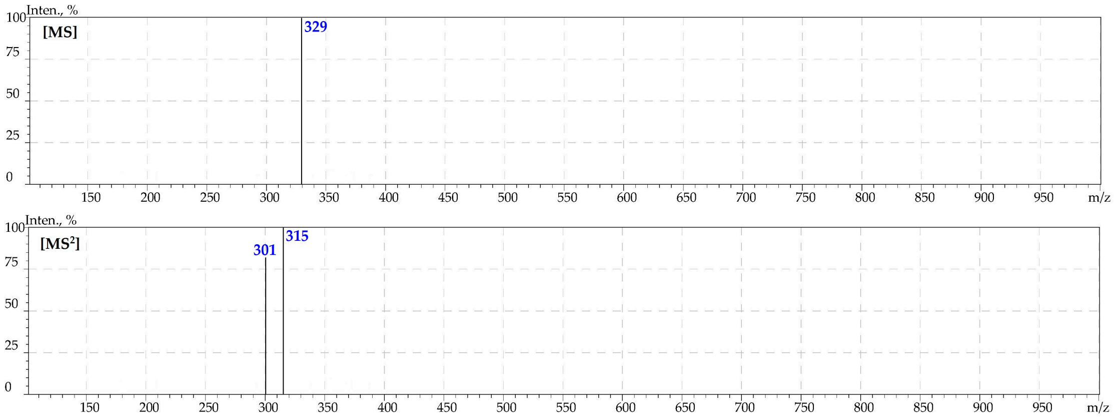

Figure S1-49. MS and MS^2 spectra of desmethylcentaureidin (compound **49**).

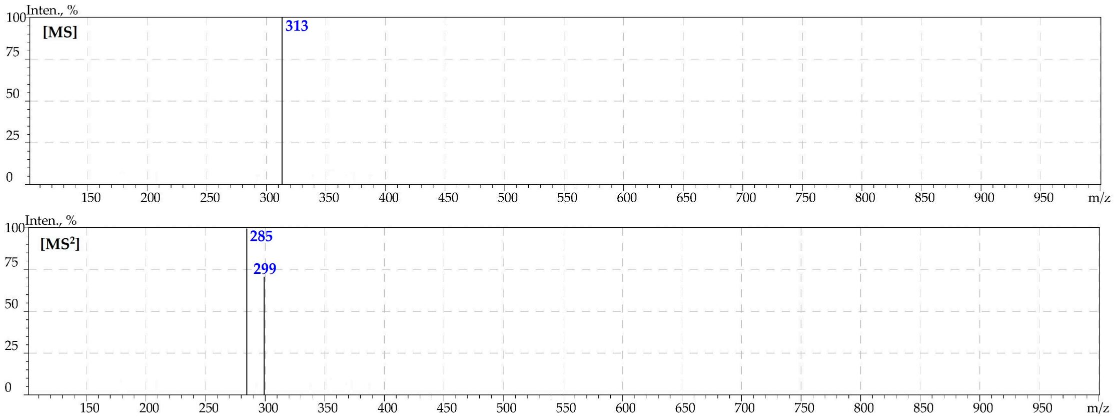

Figure S1-50. MS and MS^2 spectra of luteolin-3′,4′-dimethyl ether (compound **50**).

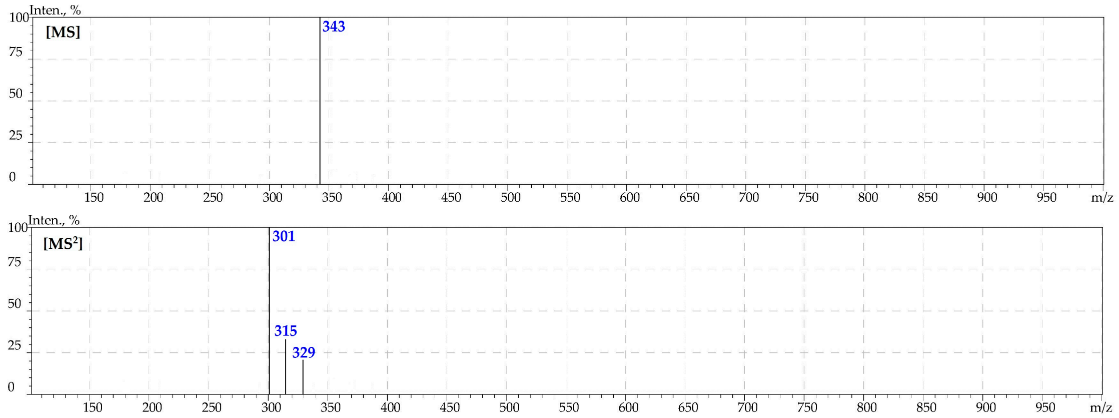

Figure S1-51. MS and MS² spectra of eupatorin (compound **51**).

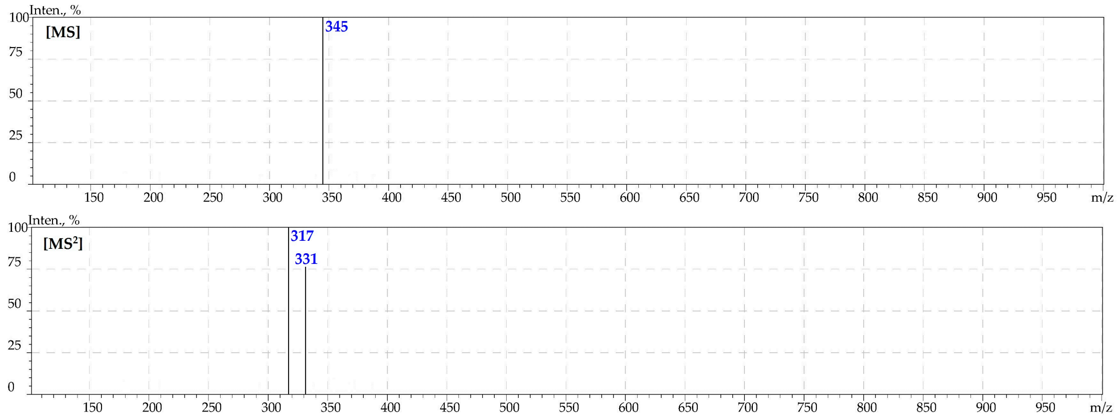

Figure S1-52. MS and MS² spectra of 5,7,3′,4′-tetrahydroxy-6,5′-dimethoxyflavone (compound **52**).

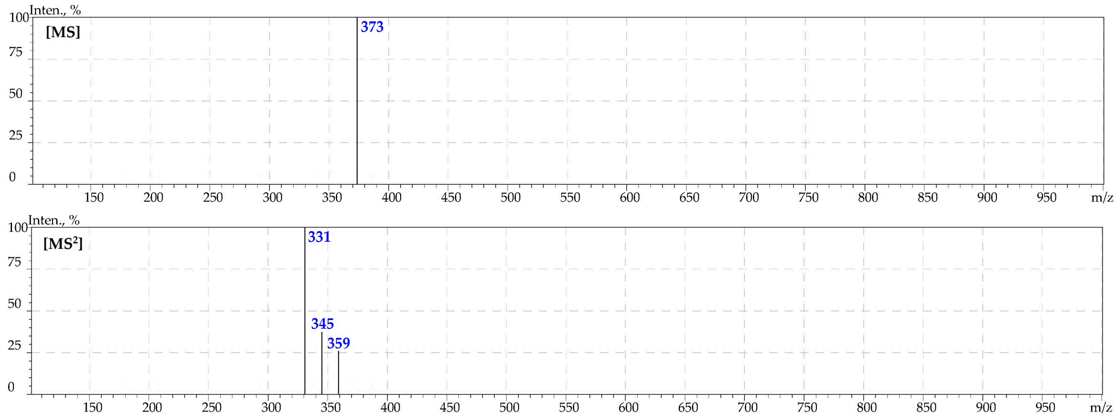

Figure S1-53. MS and MS² spectra of quercetagetin-3,6,3′,4′-tetramethyl ether (compound **53**).

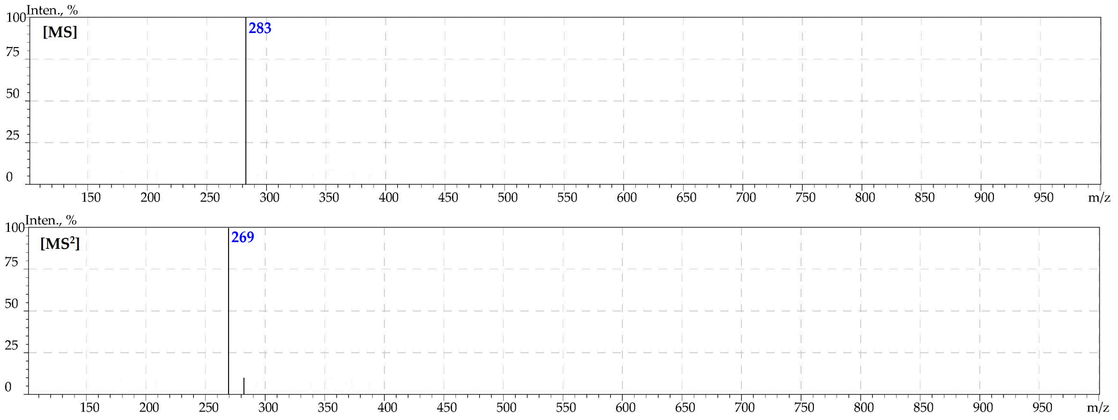

Figure S1-54. MS and MS^2 spectra of acacetin (compound **54**).

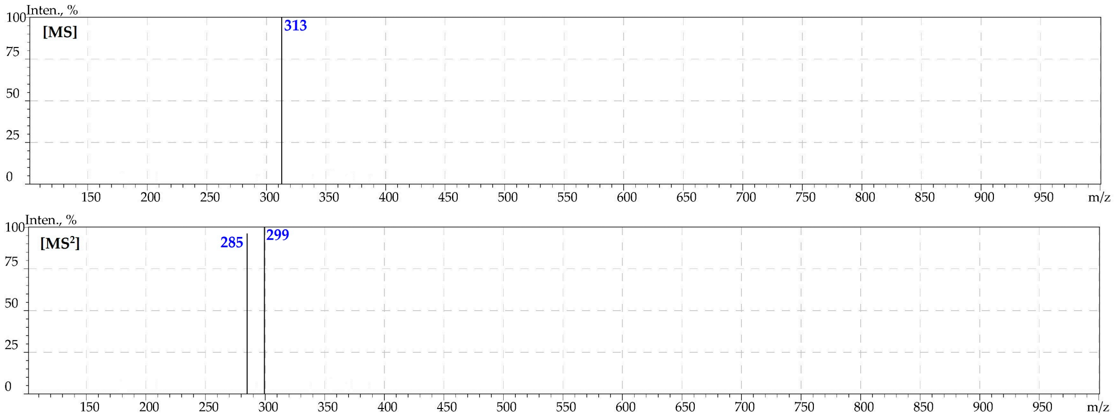

Figure S1-55. MS and MS^2 spectra of cirsimaritin (compound **55**).

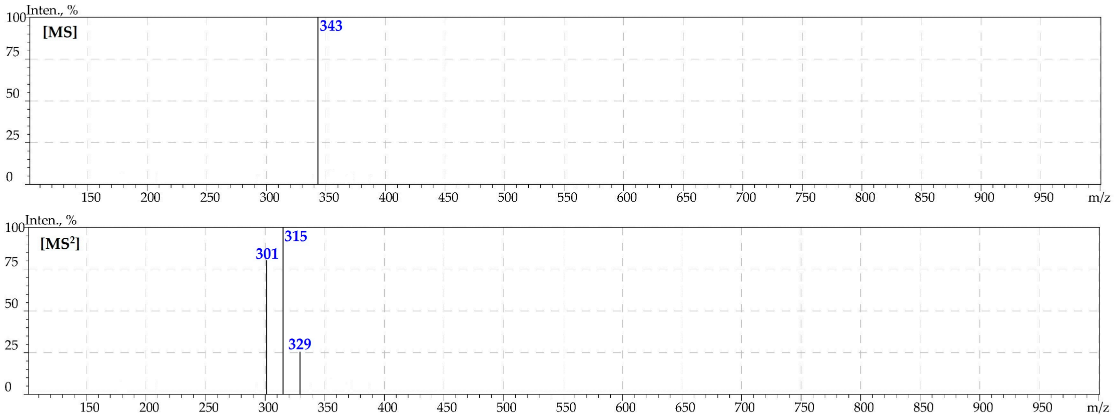

Figure S1-56. MS and MS^2 spectra of cirsilineol (compound **56**).

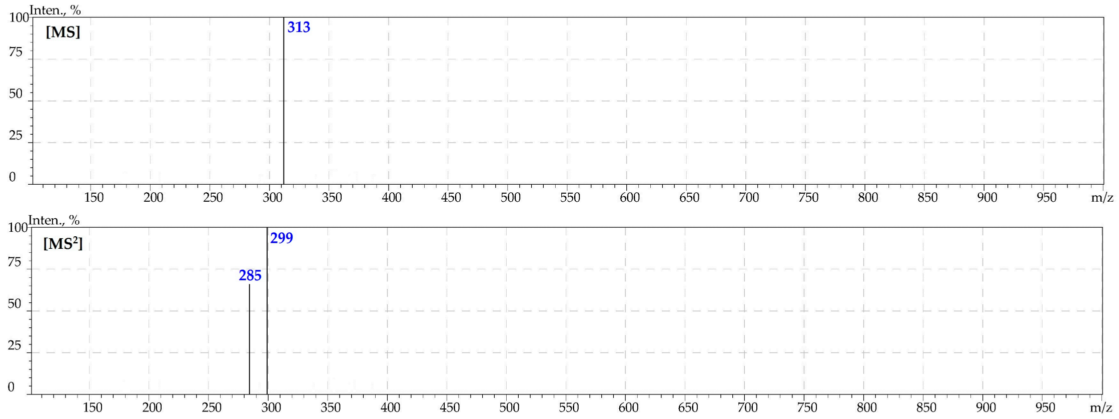

Figure S1-57. MS and MS^2 spectra of velutin (compound **57**).

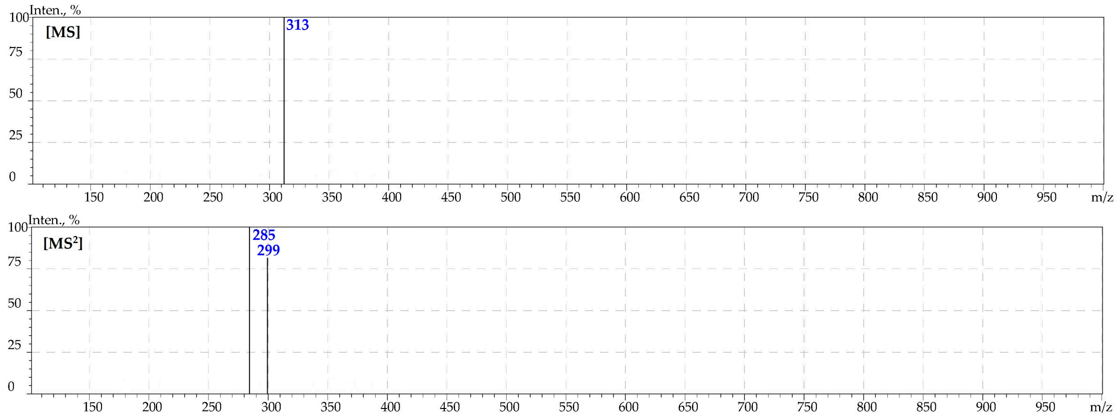

Figure S1-58. MS and MS^2 spectra of pilloin (compound **58**).

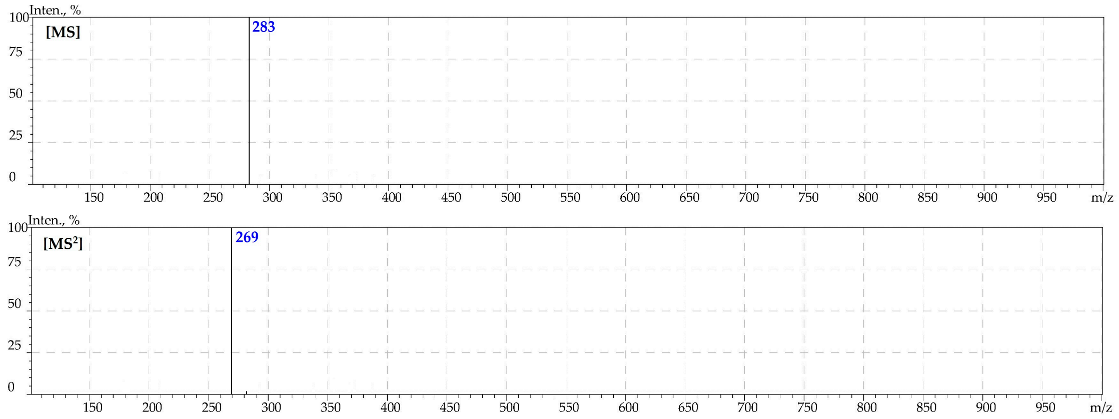

Figure S1-59. MS and MS^2 spectra of genkwanin (compound **59**)

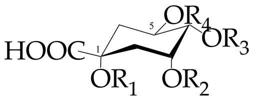

1: R_1 = Caf; R_2 = R_3 = R_4 = H
2: R_1 = R_2 = R_4 = H; R_3 = Caf
4: R_1 = R_2 = R_3 = H; R_4 = Caf
5: R_1 = R_3 = R_4 = H; R_2 = Caf
6: R_1 = R_2 = Caf; R_3 = R_4 = H
19: R_1 = R_4 = H; R_2 = R_3 = Caf
21: R_1 = R_3 = H; R_2 = R_4 = Caf
24: R_1 = R_2 = H; R_3 = R_4 = Caf
26: R_1 = R_4 = Caf; R_2 = R_3 = H
31: R_1 = R_2 = R_4 = Caf; R_3 = H
32: R_1 = R_3 = R_4 = Caf; R_2 = H
36: R_1 = H; R_2 = R_3 = R_4 = Caf

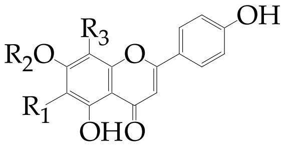

7: R_1 = R_3 = β-D-Glc*p*; R_2 = H
9: R_1 = α-L-Ara*p*; R_2 = H; R_3 = β-D-Glc*p*
11: R_1 = β-D-Glc*p*; R_2 = H; R_3 = α-L-Ara*p*
13: R_1 = R_2 =H; R_3 = β-D-Glc*p*
15: R_1 = β-D-Glc*p*; R_2 = R_3 =H
29: R_1 = R_3 =H; R_2 = β-D-Glc*p*
42: R_1 = R_2 = R_3 =H

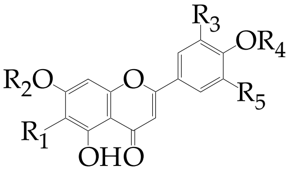

41: R_1 = R_2 = R_4 = H; R_3 = R_5 = OCH_3
43: R_1 = OCH_3; R_2 = R_3 = R_4 = R_5 = H
45: R_1 = R_3 = OCH_3; R_2 = R_4 = R_5 = H
50: R_1 = R_2 = R_5 = H; R_3 = OCH_3; R_4 = CH_3
51: R_1 = OCH_3; R_2 = R_4 = CH_3; R_3 = OH; R_5 = H
54: R_1 = R_2 = R_3 = R_5 = H; R_4 = CH_3
55: R_1 = OCH_3; R_2 = CH_3; R_3 = R_4 = R_5 = H
56: R_1 = R_3 = OCH_3; R_2 = CH_3; R_4 = R_5 = H
57: R_1 = R_4 = R_5 = H; R_2 = CH_3; R_3 = OCH_3
59: R_1 = R_3 = R_5 = R_5 = H; R_2 = CH_3

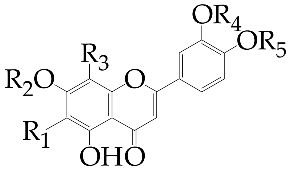

8: R_1 = β-D-Glc*p*; R_2 = R_3 = R_4 = R_5 =H
10: R_1 = R_2 = R_4 = R_5 = H; R_3 = β-D-Glc*p*
16: R_1 = OH; R_2 = β-D-Glc*p*; R_3 = R_4 = R_5 = H
17: R_1 = R_3 = R_4 = R_5 = H; R_2 = β-D-Glc*p*
18: R_1 = OCH_3; R_2 = β-D-Glc*p*; R_3 = R_4 = R_5 = H
20: R_1 = R_3 = R_4 = H; R_2 = β-D-Glc*p*; R_5 = CH_3
22: R_1 = R_3 = R_5 = H; R_2 = β-D-Glc*p*; R_4 = CH_3
23: R_1 = OCH_3; R_2 = R_3 = R_5 = H; R_4 = β-D-Glc*p*
27: R_1 = R_2 = R_3 = R_4 = H; R_5 = β-D-Glc*p*
33: R_1 = R_2 = R_3 = H; R_5 = β-D-Glc*p*; R_4 = CH_3
38: R_1 = OCH_3; R_2 = R_3 = R_4 = H; R_5 = β-D-Glc*p*
38: R_1 = OH; R_2 = R_3 = R_4 = H; R_5 = β-D-Glc*p*
47: R_1 = R_2 = R_3 = R_5 = H; R_4 = CH_3

Figure S2. Structures of reference standards used in present work. Abbreviations structures showed below.

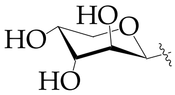

Ara*p* (arabinopyranosyl)

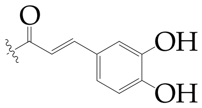

Caf (caffeoyl)

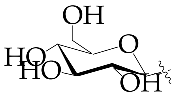

Glc*p* (glucopyranosyl)

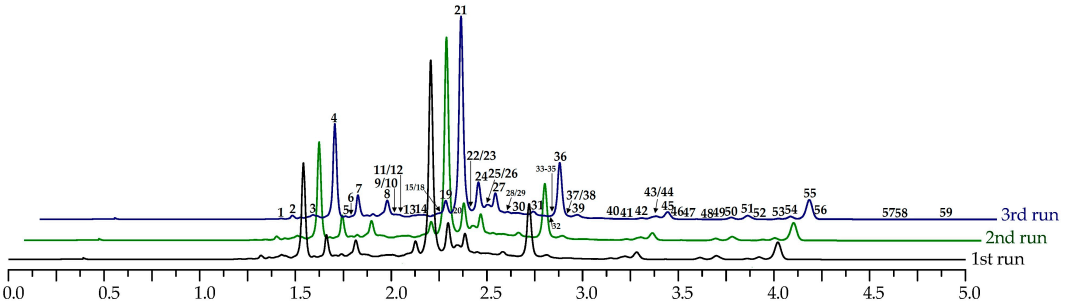

Figure S3. A series of three successive chromatograms of *A. frigida* extract demonstrating good reproducibility of the method used. Compounds are numbered as listed in Table 2.

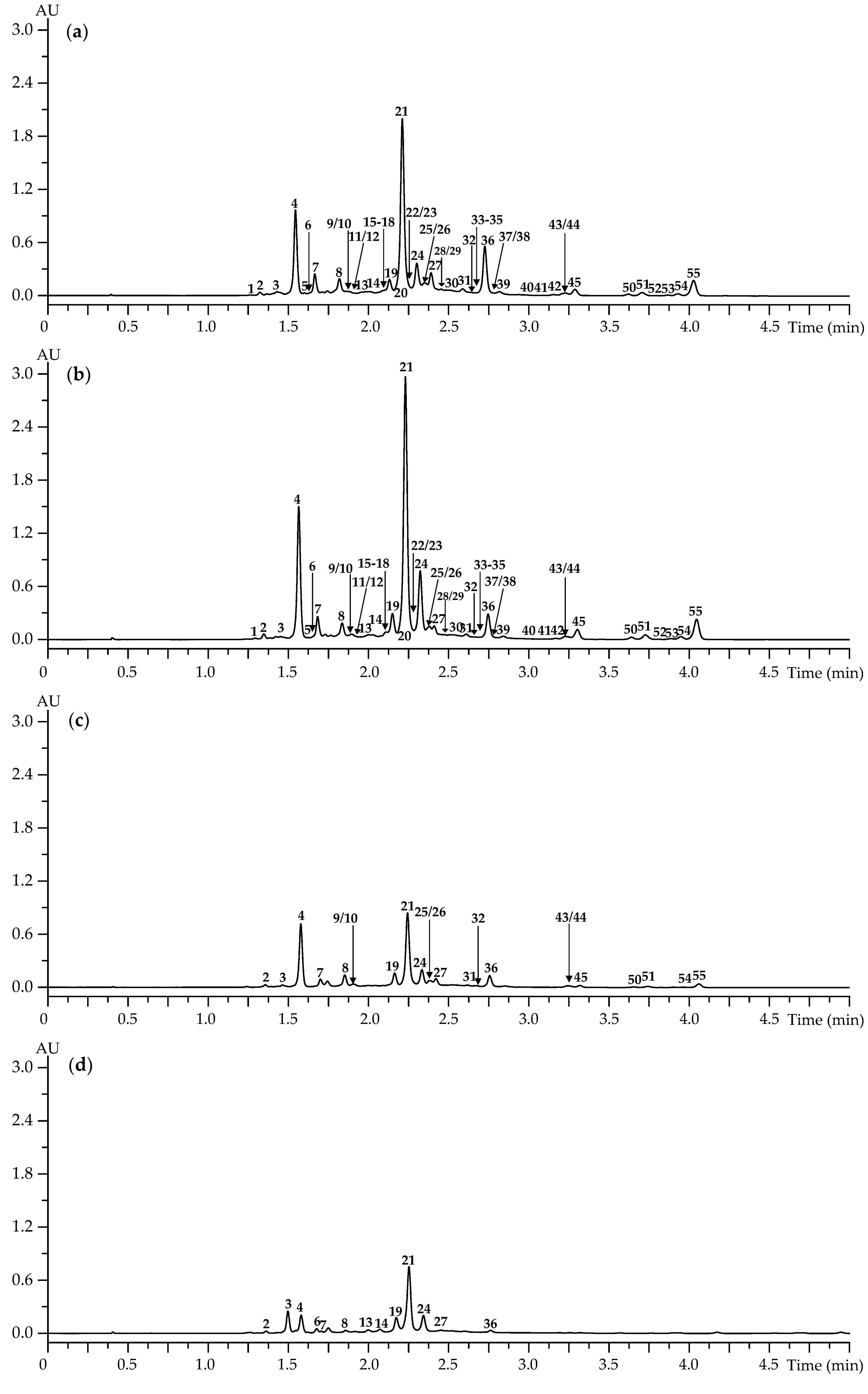

Figure S4. HPLC-DAD chromatograms (330 nm) of *A. frigida* extract of leaves (**a**), flowers (**b**), stems (**c**), and roots (**d**). Compounds are numbered as listed in Table 2.

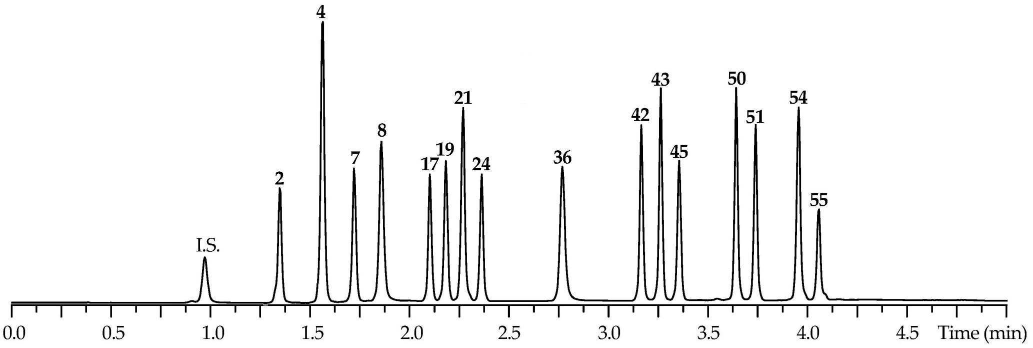

Figure S5. HPLC-DAD chromatogram of the reference mixture of 16 pure compounds and internal standard (I.S., scopoletin-7-*O*-neohesperidoside). Compounds: **2**—4-*O*-caffeoylquinic acid; **4**—5-*O*-caffeoylquinic acid; **7**—vicenin-2; **8**—isoorientin; **17**—cynaroside; **19**—3,4-di-*O*-caffeoylquinic acid; **21**—3,5-di-*O*-caffeoylquinic acid; **24**—4,5-di-*O*-caffeoylquinic acid; **36**—3,4,5-tri-*O*-caffeoylquinic acid; **42**—apigenin; **43**—hispidulin; **45**—jaceosidin; **50**—luteolin-3′,4′-dimethyl ester; **51**—eupatorin; **54**—acacetin; **55**—cirsimaritin.

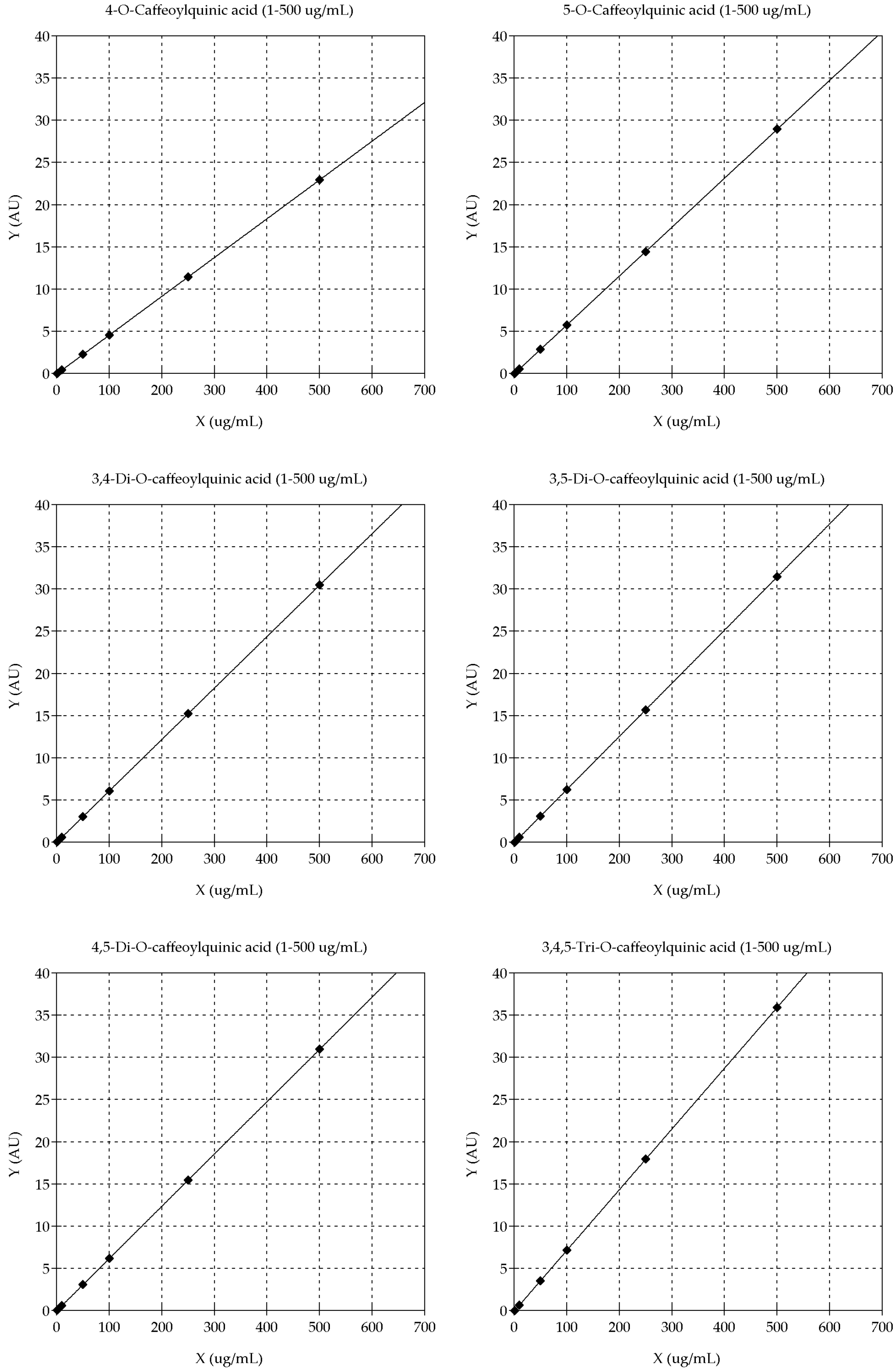

Figure S6. Calibration curves for 16 reference compounds used for quantitative HPLC-DAD assay.

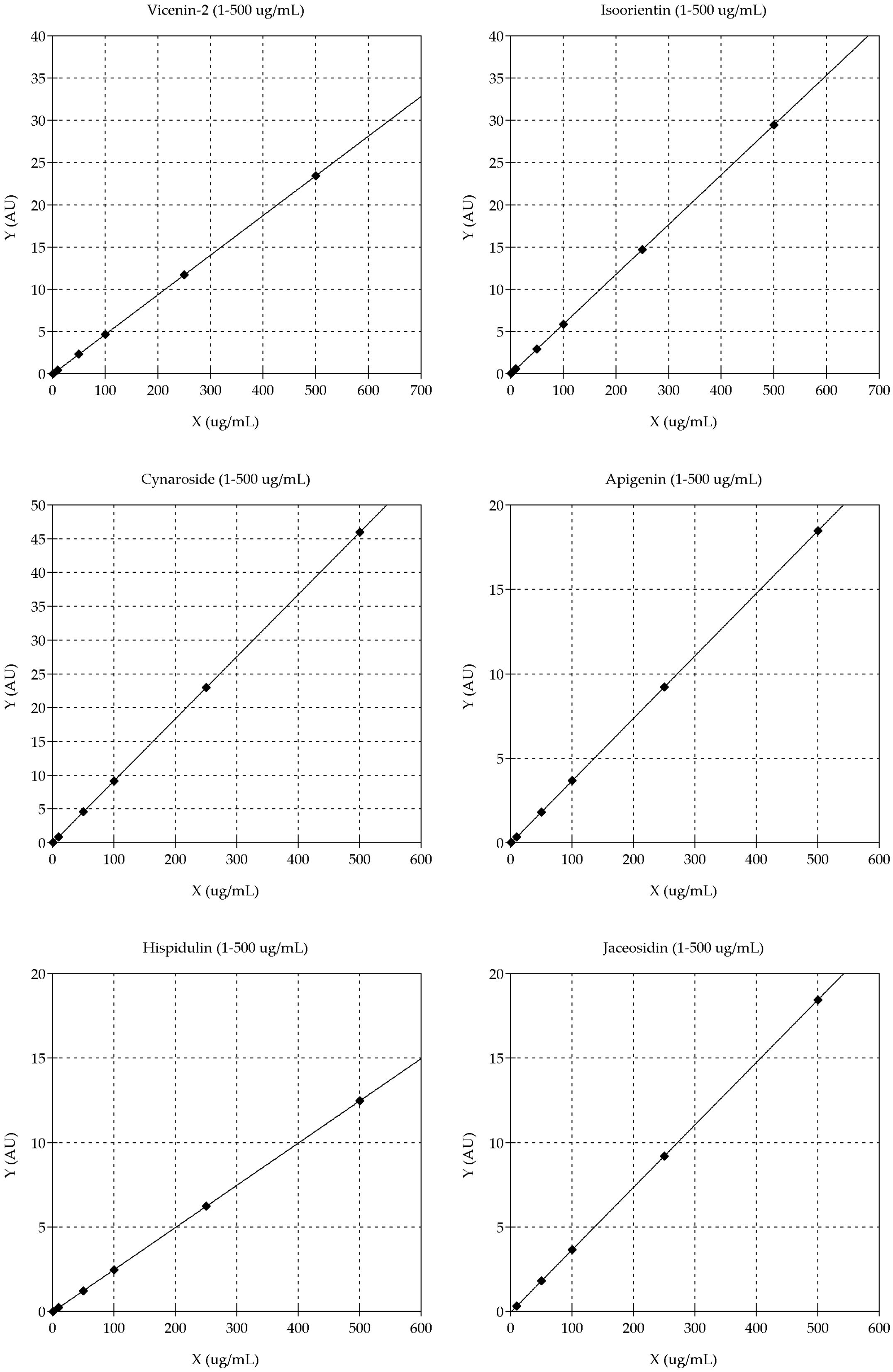

Figure S6. *Continuation.*

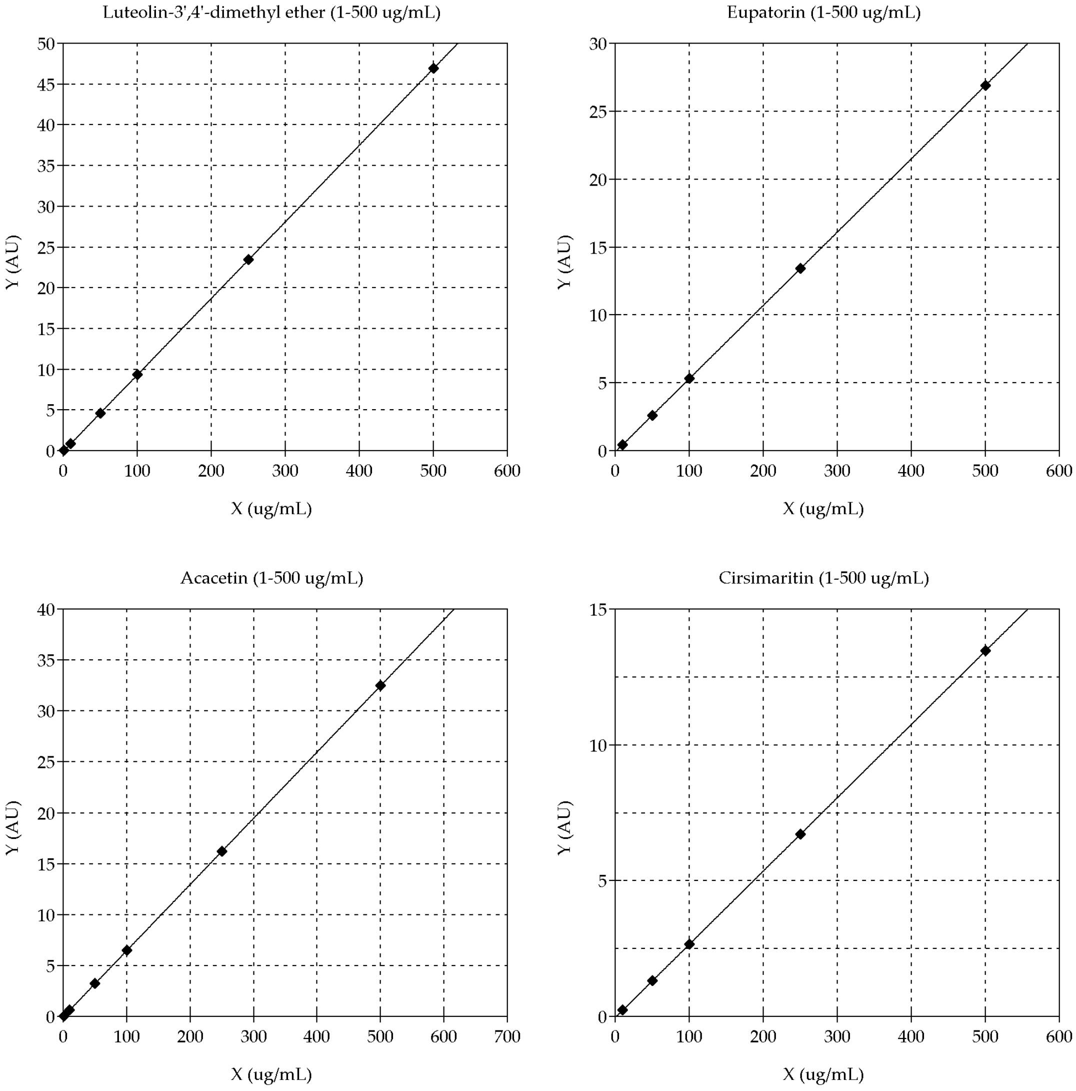

Figure S6. *Continuation.*

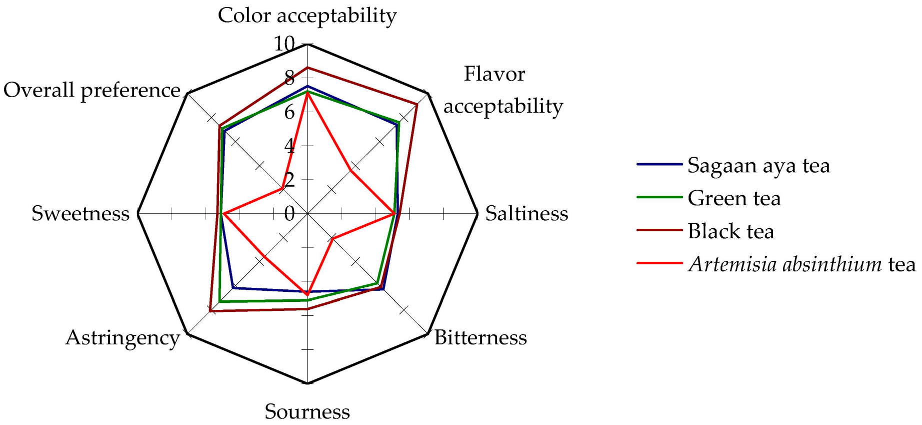

Figure S7. Sensory profiles of *A. frigida* herbal tea, green tea, black tea and *Artemisia absinthium* tea according to 30 participants estimation. Preference scale ranged from 0 (greatest imaginary dislike) to 10 (greatest imaginary like).

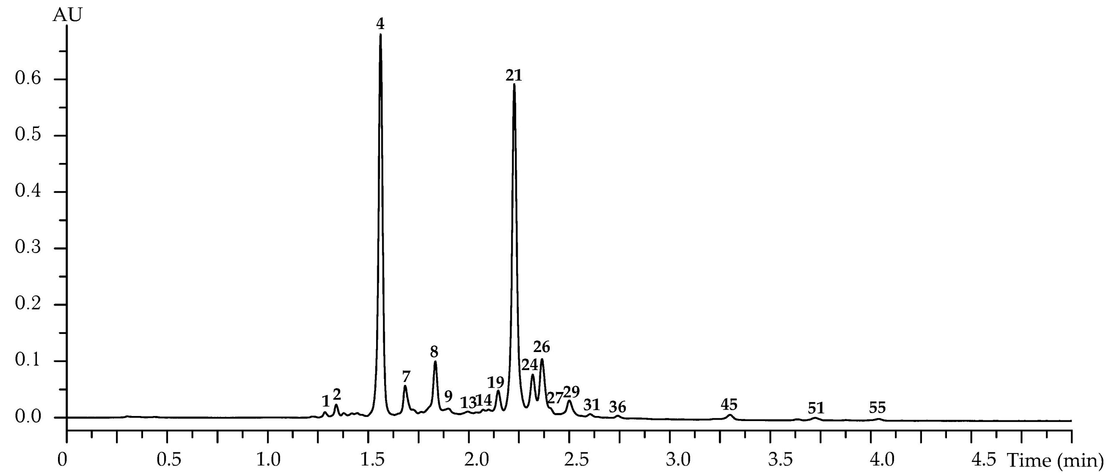

Figure S8. HPLC-DAD chromatogram of *A. frigida* herbal tea. Compounds are numbered as listed in Table 2.

© 2019 by the authors. Submitted for possible open access publication under the terms and conditions of the Creative Commons Attribution (CC BY) license (http://creativecommons.org/licenses/by/4.0/).
